# Uniform shape monodisperse single chain nanocrystals by living aqueous catalytic polymerization

**DOI:** 10.1038/s41467-019-10692-1

**Published:** 2019-06-13

**Authors:** Manuel Schnitte, Anne Staiger, Larissa A. Casper, Stefan Mecking

**Affiliations:** Chair of Chemical Materials Science, Department of Chemistry, University of Konstanz, Konstanz, 78457 Germany

**Keywords:** Polymer synthesis, Polymers

## Abstract

The preparation of polymer nanoparticles with a uniform size and shape, beyond spheres, is an unresolved problem. Here we report a living aqueous catalytic polymerization, resulting in particles grown by a single active site and composed of a single ultra high molecular weight polyethylene (UHMWPE) chain. The control on a molecular level (*M*_w_/*M*_n_ = 1.1–1.2) and at the same time on a particle level (PDI < 0.05) together with the immediate deposition of the growing chain on the growing nanocrystal results in a distinct evolution of the particle morphology over time. These uniform nanocrystals are obtained as concentrated aqueous dispersions of > 10 wt-% (*N* ≈ 10^19^ particles L^−1^) polymer content. Key to this robust procedure to single chain nanoparticles are long-lived water-stable Ni(II) catalysts that do not undergo any chain transfer. These findings are a relevant step towards polymer materials based on nanoparticle assembly.

## Abstract (web summary)

The formation of polymer nanoparticles with a uniform size and shape, beyond spheres, is an unresolved problem. Here the authors show a living aqueous catalytic polymerization forming single crystal particles grown by a single active site and composed of a single ultra high molecular weight polyethylene chain.

## Introduction

A key to nanoparticle-based materials is the ability to access nanoparticles with uniform shapes and sizes. For inorganic nanoparticles, this is a solved problem by and large. Metal or metal oxide nanoparticles with dimensions as low as a few nanometers can be generated in a variety of shapes in high quality, which allows for their further assembly.^1–5^ By strong contrast, routes to polymer nanoparticles with a uniform shape beyond spheres are lacking.

For the particular important and challenging size regime of only a few nanometers to a few tens of nanometers, single-chain particles composed of uniform chains are a straightforward theoretical concept to achieve a uniform particle size (or, more precisely, volume). Single-chain particles have been much studied, and the field continues to expand.^6–9^ They are commonly prepared by a post-polymerization collapse or assembly from solutions of separately synthesized chains. Although a shape control is recognized as one of the major potentials of single-chain nanoparticles, this has rarely been achieved.^10–13^

**Fig. 1:**
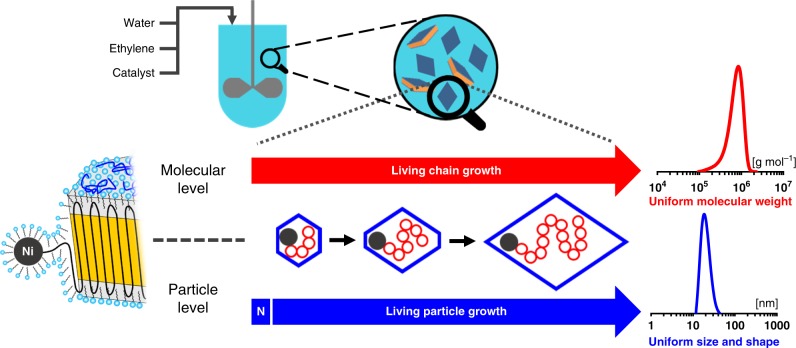
Concept to obtain uniform size and shape particles by controlled polymerization on a molecular as well as particle level. An effective nucleation (N) ensures that particles contain only one active site, and start to grow virtually at the same time. Due to the living character of polymerization, all particles continue to grow for the entire duration of the experiment, to yield particles each composed of a chain of identical length. As the growing chains are immediately deposited on the growing single-crystal particle during this process, particle shape evolves uniformly over time during polymerization

We now show a different approach, namely direct polymerization to single-chain uniform-shape monodisperse nanocrystals (Fig. 1). We demonstrate this for polyethylene, the largest and most important synthetic polymer material.

## Results

### Catalyst design

This approach poses several challenges. To generate single-chain particles rather than larger aggregates, an efficient compartmentalization is required. Water as a medium offers itself, in being a polar medium that allows for an efficient colloidal stabilization of particles, and a non-solvent for most polymers. However, this requires well-designed polymerization methods.

Traditional insertion polymerization catalysts are extremely sensitive to traces of water. This can be overcome by less oxophilic late-transition metal catalysts. With state-of-the-art catalysts, based on *N*-terphenyl salicylaldiminato Ni(II) complexes, ultra high-molecular-weight polyethylene can be generated in aqueous polymerizations.^14,15^ This is formed as nanocrystals with an unusually high degree of order that arises from the immediate deposition of the growing chain on the crystal growth front.^16^

**Fig. 2:**
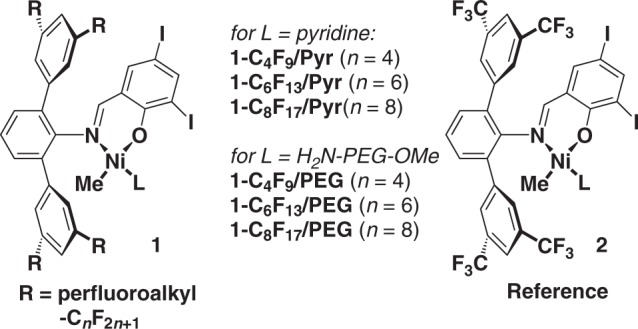
Catalysts precursors studied. Compared to the known reference system (right), the novel catalysts reported here feature perfluoroalkyl chains of variable length in the remote positions of the *N*-terphenyl moiety (left). The coordinated labile ligand L renders the catalyst precusors lipophilic (L = py), or for aqueous polymerizations hydrophilic (L = H_2_N-PEG-OMe)

Based on recent insights how the ligand environment of the active site controls chain growth and chain transfer^17^, and how active sites are terminated by water^18^, we targeted the perfluoro-substituted motifs **1** (Fig. 2). This choice is based on the argument that perfluoro groups are highly electron withdrawing, which suppresses specific chain transfer pathways, and due to their hydrophobicity^19^ possibly hinder an access of water to the active sites (for details of synthesis and characterization of all catalyst precursors, cf. Supplementary Methods). The major, yet unmet challenge toward the concept pursued here is to achieve a living nature of aqueous ethylene polymerization. This requires a strict suppression of chain transfer reactions as well as termination.

**Fig. 3:**
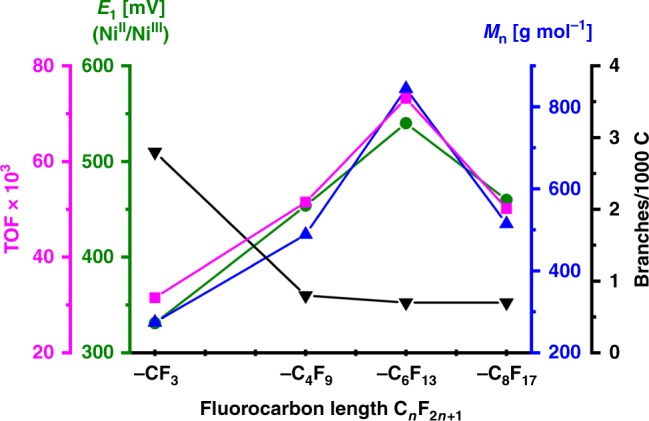
Comparison of the experimental data from polymerization experiments with different catalyst precursors. Catalytic activity (TOF, magenta line), forward peak potential determined via cyclic voltammetry of precatalysts (*E*1, green line), molecular weight (blue line) and branching values (black line) of formed polyethylenes and fluorocarbon chain length in precatalysts’ structures (**1/Pyr**) given. Experimental data from experiments at 30 °C in toluene (entries 1, 4, 7, 10, Table 1)

Pressure reactor studies of ethylene polymerization in toluene as a reaction medium with the perfluoro-substituted complexes **1/Pyr** revealed these to be precursors to highly active catalysts (Table 1). Compared with the benchmark catalyst precursor **2**, with electron-withdrawing trifluoromethyl remote substituents, catalyst activities (16 TO h^−1^ vs. 761 TO h^−1^, entries 3 and 9, Table 1 and Fig. 3, magenta line) and molecular weights (27 × 10³ g mol^−1^ vs. 308 × 10³ g mol^−1^, entries 2 and 8, Table 1 and Fig. 3, blue line) are substantially increased. More than 400.000 mole ethylene can be polymerized per mole of catalyst precursor per hour. At the same time, the amount of branches in the polymer is decreased to less than one branch per 1000 carbons, which is desirable to facilitate crystallization (vide infra). Notably, narrow molecular weight distributions indicative of a living polymerization are observed in some cases, as low as *M*_w_/*M*_n_ < 1.3.

**Table 1:** Ethylene polymerization results in toluene^a^

Entry (cond.)	Precatalyst	T [C°]	Yield [g]	TOF^b^	*M*_n_^c^ [10^3^ g mol^−1^]	*M*_w_/*M*_n_^c^	*T*_m_^d^ [°C]	Crystallinity^d^ [%]	Branches/1000 C^e^
1^A^	2/Pyr	30	2.21	31.5	275	1.5	133	48	2.8
2^B^	2/Pyr	50	2.87	136.4	27	1.7	121	47	9.1
3^C^	2/Pyr	70	0.17	16.2	8	2.0	114	47	14.0^f^
4^A^	1-C_4_F_9_/Pyr	30	3.60	51.4	489	1.5	133	58	0.8
5^B^	1-C_4_F_9_/Pyr	50	6.43	305.6	305	1.3	131	49	2.2
6^D^	1-C_6_F_13_/Pyr	15	5.26	6.3	1190	1.3	136	49	n.d.
7^A^	1-C_6_F_13_/Pyr	30	5.13	73.2	844	1.5	134	56	0.7
8^B^	1-C_6_F_13_/Pyr	50	8.17	388.4	308	1.4	131	46	1.9
9^C^	1-C_6_F_13_/Pyr	70	8.00	760.6	33	1.6	125	50	6.5
10^A^	1-C_8_F_17_/Pyr	30	3.52	50.2	515	1.5	135	60	0.7
11^B^	1-C_8_F_17_/Pyr	50	3.83	182.1	218	1.5	132	46	1.7

^a^Polymerization conditions: (A) 5 µmol precatalyst, 30 -min reaction time, (B) 3 µmol precatalyst, 15 min reaction time, (C) 3 µmol precatalyst, 7.5 min reaction time, (D) 5 µmol precatalyst, 5 h reaction time; all experiments performed in 100 mL of toluene with 40 bar ethylene pressure; ^b^given in 10^3^ x mol [C_2_H_4_] x mol^−1^ [Ni] x h^−1^_._^c^Determined by GPC at 160 °C in trichlorobenzene. ^d^Determined by DSC with 10 K min^−1^ heating rate (data from second heating cycle). ^e^Determined by ^13^C NMR spectroscopy. ^f^Including 2.3 ethyl branches

Both chain transfer, which limits molecular weights and broadens molecular weight distributions, and branch formation proceed through ß-H elimination as an underlying reaction step. Correspondingly, an increase in molecular weight goes along with a reduction of branching, both desirable features. The pathway to ß-H elimination is known to be suppressed by electron-withdrawing substituents.^17^ Cyclic voltammetry studies on the catalyst precursor of the oxidation and reduction transitions for the Ni(II)/Ni(III) pair show that compared with the established trifluoromethyl substitution, indeed the electron density at the metal is lowered significantly in the perfluoroalkyl complexes (Fig. 3, green line), and this correlates qualitatively with the observed catalytic properties. The origin of the lower potential for *n* = 8 vs. *n* = 6 remains unclear at this point.

### Aqueous polymerization and particles

Due to their superior performance, perfluorohexyl substituents were used in the further studies of aqueous polymerization. For polymerizations in aqueous systems, hydrophilic catalyst precursors with a weakly coordinating amino-polyethylene glycol ligand were generated (**1/PEG**). A space of various experimental parameters was optimized in pressure reactor experiments with regard to a uniform nature of the formed particles and catalyst performance, among others surfactant type and concentration, pH, reaction temperature, and sonication (cf. Supplementary Methods for comprehensive data). Other than **2/PEG**, the perfluorinated catalyst precursors **1/PEG** do not form homogenous molecular solutions, and a small amount of hydrophobic organic solvent (0.2–0.5 vol-%) is required to obtain a highly dispersed system. From a range of solvents, mesitylene evolved as the solvent of choice. This enabled very narrow distributed sub-100-nm particle sizes (cf. Supplementary Figs. 56, 57 for dynamic light scattering (DLS) traces of PE dispersions). We attribute this to an undesired agglomeration of the catalyst precursor molecules via their very hydrophobic portions in the aqueous system in the absence of the small organic solvent volume.

Under the identified reaction conditions, catalytic activity was retained for > 4 h. Experiments conducted with different reaction times provided detailed insights into the chain and particle growth process (Table 2). For all experiments, stable and transparent to slightly opaque PE dispersions were obtained.

**Fig. 4:**
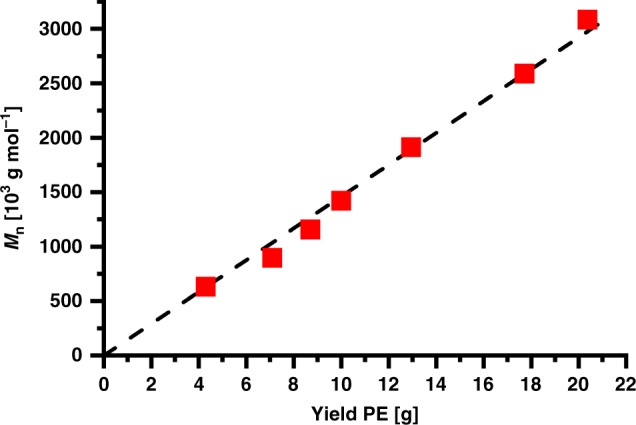
Molecular weights of the polyethylenes formed versus yields. The data for aqueous polymerization experiments, with reaction times varying from 0.5 to 4 h (data from Table 2)

**Fig. 5:**
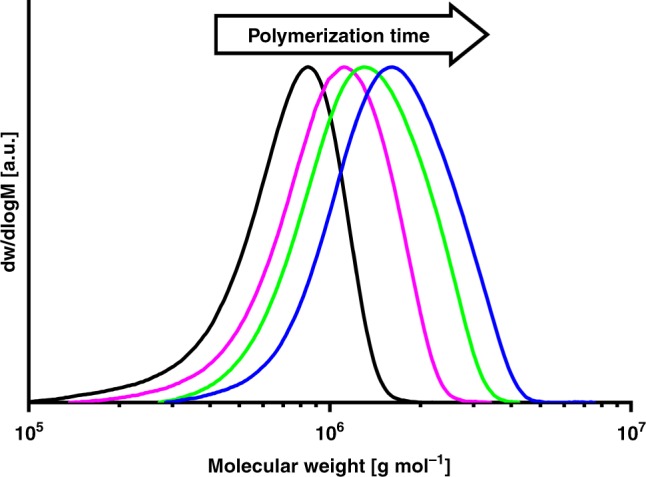
GPC traces of narrow distributed UHMWPEs obtained in aqueous polymerization after different reactions times. Black curve: 0.5 hours reaction time, magenta curve: 1 h reaction time, green curve: 2 h reaction time, blue curve: 4 h reaction time (data from Table 2, entries 1–4)

Remarkably, this aqueous catalytic polymerization is truly living in nature, as evidenced by (1) a linear relationship between yields and molecular weights (Fig. 4); (2) very narrow molecular weight distributions of *M*_w_/*M*_n_ = 1.1–1.2; and (3) chains to Ni ratios of unity. As a given nickel center grows a single chain over the entire reaction time, linear narrow distributed UHMWPE with *M*_n_ > 10^6^ g mol^−1^ (*M*_w_/*M*_n_ = 1.2, cf. Fig. 5) can be produced (Table 2, entry 4). Even molecular weights in excess of *M*_n_ 3 × 10^6^ g mol^−1^ are accessible (Table 2, entry 7). DSC studies of isolated polymers indicate a non-entangled nature of the formed UHMWPE, as indicated by a high first-cycle melting temperature of > 140 °C, which is not observed for slow heating rates in all cases.^20^ The polymer microstructure is virtually devoid of any branches (< 0.7 branches per 1000 carbons) as revealed by ^13^C NMR analysis, in accordance with the observed melting properties. The aqueous polymerization procedure yields the UHMWPE efficiently with high particle number densities (up to 13 wt-%, corresponding to *N* = 10^19^ particles L^−1^), precisely tunable by surfactant content and catalyst loading. This compares favorably to the state-of-the-art systems of high concentration single-chain nanoparticle dispersions^21,22^ and differs from many post-polymerization procedures, which yield only low particle concentrations.

**Table 2:** Ethylene polymerization results of precatalyst 1-C_6_F_13_/PEG in aqueous surfactant solution^a^

Entry (cond.)	Time [hours]	Yield [g]	TON^b^	*M*_n_^c^ [10^3^ g mol^−1^]	*M*_w_/*M*_n_^c^	Chains/[Ni]	*T*_m_^d^ [°C]	Cryst.^d^ [%]	Particle size [nm] (DLS)^e^	Particle size [nm] (TEM)^f^	Chains/particle
1^A^	0.5	4.29	20.4	631	1.2	0.9	142/136	66/41	18	23.6 ± 1.6	1.2 ± 0.3
2^A^	1	7.09	33.7	896	1.2	1.1	143/135	64/43	20	28.9 ± 2.2	1.2 ± 0.3
3^A^	2	8.67	41.2	1154	1.2	1.0	142/135	66/43	23	33.6 ± 2.3	1.3 ± 0.3
4^A^	4	9.95	47.3	1421	1.2	0.9	143/136	64/42	24	36.8 ± 2.8	1.2 ± 0.3
5^B^	1	12.94	61.5	1912	1.2	0.9	143/134	60/37	32	46.0 ± 6.1	1.4 ± 0.3
6^B^	2	17.72	84.2	2588	1.2	0.9	144/135	62/39	36	53.9 ± 9.1	1.5 ± 0.4
7^B^	4	20.37	96.1	3084	1.3	0.9	143/135	64/43	42	64.4 ± 12.3	1.7 ± 0.4

^a^Polymerization conditions: (A) 7.5 µmol precatalyst, 40 bar ethylene pressure, 6.0 g of sodium dodecyl sulfate, 1.5 g of cesium hydroxide, 0.75 mL of mesitylene, in 150 mL of water, 15 °C reaction temperature; (B) 7.5 µmol precatalyst, 40 bar ethylene pressure, 12.0 g of sodium dodecyl sulfate, 3.0 g of cesium hydroxide, 1.5 mL of mesitylene, in 300 mL water, 15 °C reaction temperature; the catalyst solution was ultrasonicated prior to pressurization with ethylene under all conditions; ^b^given in 10^3^ x mol [C_2_H_4_] x  mol^−1^ [Ni] ^c^Determined by GPC at 160 °C in trichlorobenzene. ^d^Determined by DSC on nascent polymer powder, isolated by precipitation from the nanocrystal dispersion, measured with 10 K min^−1^ heating rate, first and second heating cycle reported. ^e^Volume mean given. ^f^Lateral particle size determined from TEM statistics (equivalent diameter and standard deviation reported)

**Fig. 6:**
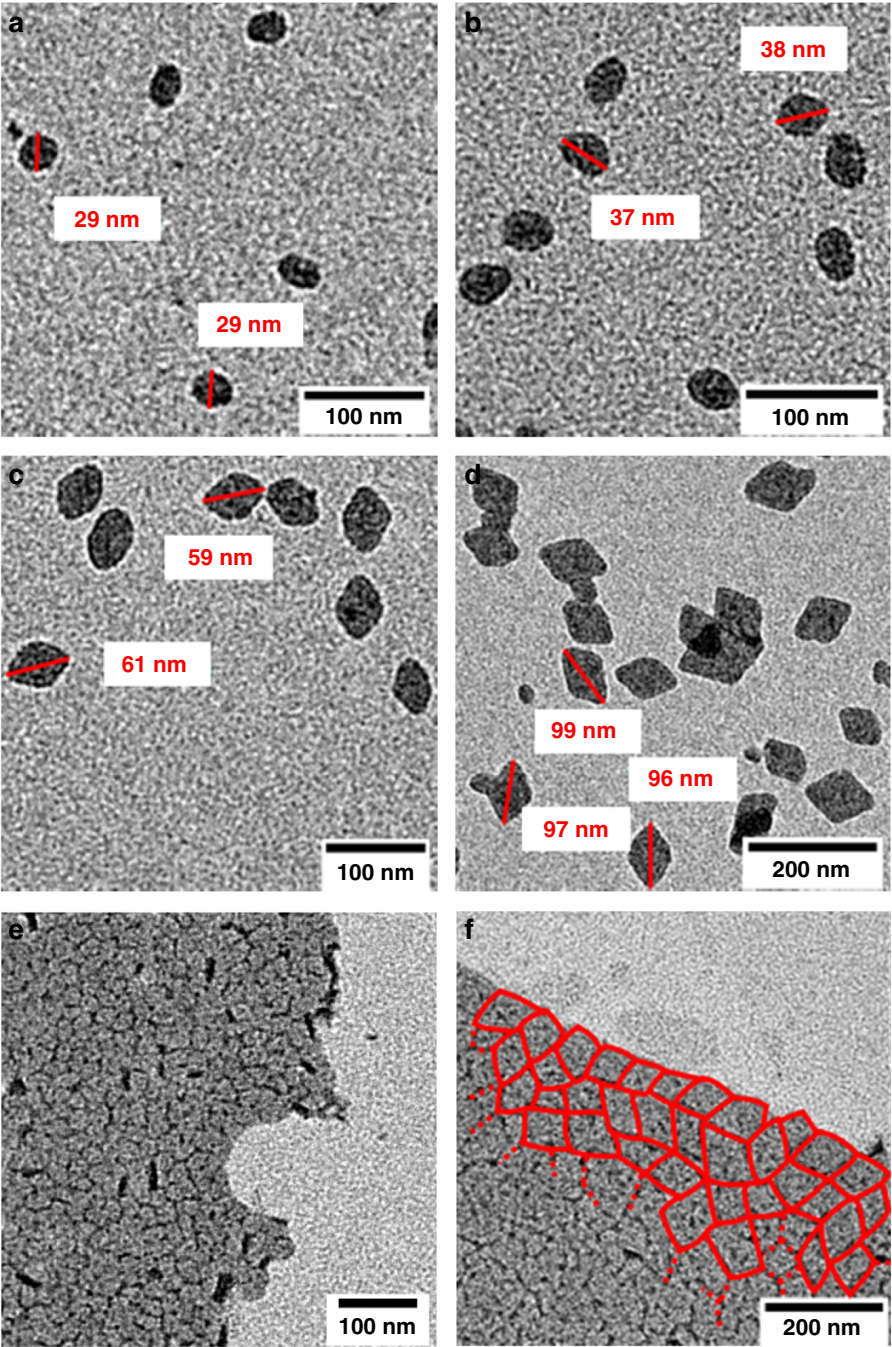
TEM images of UHMWPE nanocrystals. **a**–**d** Nanocrystals obtained from aqueous polymerization after different reaction times showing the evolution of size and shape (entries 1 (**a**), 3 (**b**), 5 (**c**), and 7 (**d**), Table 2); **e**, **f** layered structures with short-range order formed by drying of uniform particle dispersions with different sizes (entries 2 (**e**) and 7 (**f**), Table 2), particle boundaries marked in red. For full set of original TEM images cf. Supplementary Figs. 19–27

**Fig. 7:**
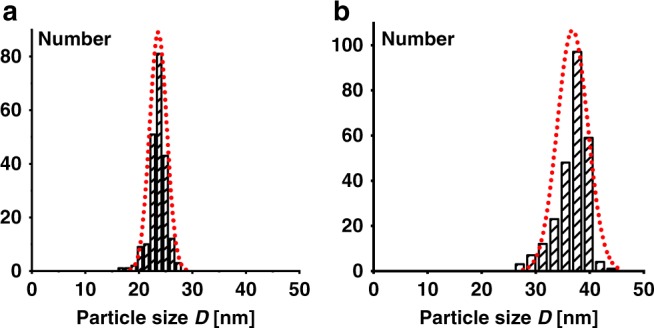
Histograms of particle sizes (equivalent diameter *D*) based on TEM statistics from obtained polyethylene dispersions. **a** After 30 min reaction time (entry 1, Table 2); **b** after 4 h reaction time (entry 4, Table 2). The dotted lines are calculated normal distribution curves

Transmission electron microscopy (TEM) reveals that the particle shape evolves from truncated lozenges to lozenges (due to a preferred deposition on the {110} crystalline growth front). A uniform shape and size-distribution and a non-aggregated nature of the particles is observed in all cases (cf. Fig. 6a–d). The aforementioned very narrow size distributions from DLS are further underlined by TEM statistical data (Fig. 7).

A comparison of the average mass of a particle (cf. Supplementary Methods for detailed size analysis from TEM and AFM data) with the molecular weight of the formed polymer shows the particles to be composed of a single chain (Table 2). That is, a given particle is grown by one active Ni(II) site, and the final particle size and morphology are determined by the time available for growth, given by the duration of the polymerization experiment.

Concerning the potential of these anisotropic particles for assembly, samples that were dried without any additional manipulations (e.g., patterned surface, spin coating, etc.) to enable the particles to arrange, show a preferred orientation of adjacent particles (Fig. 6e, f).

For anisotropic inorganic particles, like gold nanorods, micron scale highly ordered assemblies can be achieved in some cases.^23,24^ These evolved from extensive studies and consequent careful choice of suitable preparative routes, aspect ratios, and assembly conditions.^25,26^ While the above assemblies of our particles do not fully match the extensively optimized assemblies of inorganic nanoparticles, they appear promising. A direct comparison with anisotropic polymeric materials is difficult, since there are only few reports available. Recently, several examples (e.g., rod-like) were presented and show assemblies in comparable quality to ours.^27–29^

## Discussion

Anisotropic polymer nanoparticles with a uniform shape and size are accessible by aqueous catalytic polymerization. The key to this is a truly living catalytic polymerization that retains its living character for hours and up to very high-molecular-weights, providing single-chain nanocrystals of ultra high-molecular-weight polyethylene. This is enabled by advanced catalysts with highly hydrophobic perfluoroalkyl substituents, and a robust polymerization procedure that allows for a homogeneous non-aggregated solution of the catalyst precursors in the initial reaction mixture, despite their highly hydrophobic portions. Preliminary observations indicate the particles’ ability to assemble. This concept, demonstrated for polyethylenes as an important synthetic material, paves the way for particle-based materials.

## Methods

### Materials

Materials and general considerations are available in Supplementary Notes 1 (Supplementary Information).

### Ligand and complex synthesis

Detailed synthetic preparation procedures and characterization data of ligands and complexes are available in Supplementary Methods (Supplementary Information).

### Polymerization experiments and particle analysis

General procedures for ethylene polymerizations in toluene and water, process design procedures for aqueous polymerizations and statistical particle size calculations for PE nanocrystal dispersions are available in Supplementary Methods (Supplementary Information).

## Supplementary information

Supplementary Information

## Data Availability

The authors declare that data supporting the findings of this study are available within the paper and its Supplementary Information files. Supplementary Information contains detailed experimental procedures and compound characterization data, cyclic voltammograms (Supplementary Fig. 8), AFM images (Supplementary Figs. 16, 17), histograms from particle size statistics (Supplementary Fig. 18), PE nanocrystal TEM images (Supplementary Figs. 19–27), NMR spectra of complexes (Supplementary Figs. 28–36), GPC traces (Supplementary Figs. 37–48), DSC traces (Supplementary Figs. 49–55), DLS data (Supplementary Figs. 56, 57), and NMR spectra of polyethylenes (Supplementary Figs. 58–62). All data are available from the corresponding author upon reasonable request.
