# Supplementary material for: Uniform shape monodisperse single chain nanocrystals by living aqueous catalytic polymerization
Source: Nat Commun. 2019 Jun 13;10:2592. doi: 10.1038/s41467-019-10692-1 (PMC6565736; doi:10.1038/s41467-019-10692-1)
Supplement: Supplementary file 1 — Supplementary Information [file 41467_2019_10692_MOESM1_ESM.pdf]

Supplementary Information

Uniform Shape Monodisperse Single Chain Nanocrystals by Living Aqueous Catalytic Polymerization

Schnitte et al.

Contents

1. Supplementary Note 1……………………………………………………..…………………..S3

2. Supplementary Methods……………………………………………………………………….S5

2.3 Ligand synthesis…………………………………………………………………………...S5

2.4 Synthesis of complexes………………………………………………………………… S16

2.5 Cyclic voltammetry of complexes……………………………………………………………S23

2.6 Polymerization procedures……………………………………………………………………S25

2.7 Process design for polymerization in aqueous media……………………………………S27

2.8 Particle size statistics of PE nanocrystal dispersions………………………………………S34

2.9 AFM measurements……………………………………………………………………...S38

3. Supplementary Figures……………………………………………………………………….S39

3.1 Histograms from particle size TEM statistics……………………………………………..S39

3.2 TEM images of PE nanocrystals…………………………………………………………..S40

3.3 NMR spectra of complexes……………………………………………………………………S46

3.4 GPC traces of synthesized polyethylenes………………………………………………..S51

3.5 DSC traces of polyethylenes obtained in aqueous polymerization……………………… S57

3.6 DLS data of nanocrystal dispersions………………………………………………………S64

3.7 NMR spectra of synthesized polyethylenes…………………………………………………S66

4. Supplementary References…………………………………………………………………..S69

1. Supplementary Note 1

All manipulations involving air- and/or moisture-sensitive substances were carried out under inert atmosphere using standard Schlenk and glovebox techniques. Solvents were dried and degassed using standard laboratory techniques. Pentane, diethyl ether, toluene and dichloromethane were dried and freed from oxygen by passing over columns with BASF R3-11 catalyst and molecular sieves. Heptane was distilled over calcium hydride. Dioxane, m-xylene and benzene, purchased from Merck, were distilled over sodium. Pyridine, purchased from Merck, was destilled from potassium hydroxide. Mesitylene, purchased from sigma-aldrich, was distilled under nitrogen (first fraction of 10 % discarded). Water was deoxygenated by distillation under a constant nitrogen stream. Ethylene 3.5 was purchased from Air Liquide and used as obtained. 1,3-Dibromobenze and [Pd(dba)$_2$] were purchased from TCI chemicals and used as obtained. Copper powder, 4,4'-di-tert-butyl-2,2'-dipyridyl, methanol, p-toluene sulfonic acid, 1,3-bis(trifluoro)methylbenzene, 1,3-difluorobenzene, 1,3,5-triisopropylbenzene were purchased from sigma-aldrich and used as obtained (liquids were dried over molecular sieves). Perfluorobutyliodide and perfluorooctyliodide, purchased from Apollo Scientific, were degassed and dried over molecular sieves. Perfluorohexyliodide and perfluoroheptane, purchased from fluorochem, were degassed and dried over molecular sieves. Bis(pinacolato)diboron, purchased from Activate Scientific, was used as obtained. 2,6-Dibromoaniline, cesium fluoride, 3,5-diiodosalicylaldehyde and hexafluorobenzene, purchased from abcr, were used as obtained (liquids were dried over molecular sieves). Triphenylphosphine, purchased from acros, was used as obtained. [(tmeda)NiMe$_2$], purchased from MCat, was stored at -30 °C prior to use. Sodium sulfate and sodium chloride, purchased from Fisher Scientific, were used as obtained. H_2N-PEG-OMe, purchased from Iris Biotech, was stored at -30 °C prior to use. Molecular sieves (4 Å, 0.4 nm, Type 514) were purchased from Carl Roth. NMR solvents, purchased from Eurisotop, were purged with nitrogen and dried over molecular sieves. The reference catalysts and [Ir(COE)$_2$Cl]$_2$ were synthesized according to reported procedures.[1-3]

NMR spectra were recorded on a Bruker Avance III 400 with a BBFO plus probe with Z-gradient, Bruker Avance III HD 400 with a TBO probe with Z-gradient or a Bruker Avance III 600. Chemical shifts were referenced to the signal of the solvent (residual proton signal for ^{1}H spectra, carbon signal for ^{13}C spectra). Multiplicities are reported as follows: s (singlet), doublet (d), triplet (t), quartet (q), quintet (quint.), pentet (p), virtual multiplet (v), multiplet (m), broad (br.) and combination thereof. The NMR assignments were confirmed by common 2D NMR experiments (^{1}H,^{1}H-gCOSY; ^{1}H,^{13}C-gHSQC and ^{1}H,^{13}C-gHMBC). MestReNova software by Mestrelab Research S.L. was used for data evaluation. High temperature NMR spectroscopy of polymers was carried out at 130 °C in $C_2D_2Cl_4$ with 0.5 wt-% Cr(acac)$_3$ as relaxation agent. ^{13}C NMR spectra of polymers were referenced to the carbon signal of the solvent (74.4 ppm).

The total integral of all polyethylene signals was set to 1000 C and the branch content determined from the values for the α-methyl signal (37.6 ppm) and the methyl signal (20.1 ppm). Elemental analysis were carried out on an Elementar vario MICRO cube instrument at the Department of Chemistry at the University of Konstanz. Molecular weights of synthesized polyethylenes were determined by high temperature gel permeation chromatography (GPC). A Polymer Laboratories 220 instrument (software: Cirrus Multi Offline GPC/SEC software, version 3.3) equipped with PLgel Olexis columns at 160 °C in 1,2,4-trichlorobenzene (1.0 mL $minute^{-1}$ flow rate) using refractive index, viscosity and light scattering detection (15° and 90°) was used. High molecular weights (>100,000 g mol^{-1}) were determined using the triple detection method calibrated with narrow polystyrene standards. Low molecular weights (<100,000 g mol^{-1}) were determined by linear calibration versus polyethylene standards with concentration detection (refractive index) only. Differential scanning calorimetry (DSC) measurements of polymers were carried out on a Netzsch DSC 204 F1 instrument (software: Netzsch Proteus Thermal Analysis, version 6.1.0) with a heating/cooling rate of 10 K min^{-1}. Additional measurements to investigate the polymer chain disentanglement were done with a heating/cooling rate of 1 K min^{-1} (only first heating cycle reported). Dynamic light scattering (DLS) was performed on diluted polyethylene dispersions using a Malvern Zetasizer Nano-ZS ZEN 3600 instrument (633 nm) in backscattering mode (173°) at 25 °C. The data was analyzed to yield particle size distributions and polydispersity indices (PDIs; dimensionless number between 0 and 1; 1 being highly polydisperse; determined from gradient of cumulants analysis) using the Malvern Zetasizer Software, version 7.12. Transmission electron microscopy (TEM) images were recorded on a Zeiss Libra 120 EF-TEM instrument. Polyethylene dispersion samples were dialyzed versus demineralized water using Spectra/Por Dialysis Membranes 2, MWCO 12 – 14 kD and TEM samples prepared by drop casting (diluted to 0.01 wt-%) directly on the grid. The decrease of surfactant content during dialysis was monitored by tensiometry measurements. TEM images were analyzed using iTEM and ImageJ/Fiji software. Atomic force microscopy (AFM) was performed on a JPK NanoWizard instrument. Samples were prepared from dialyzed dispersions by drop casting and spin coating on a flat silicon waver. Tensiometry measurements were carried out on a Krüss Process Tensiometer K100 (software: Krüss Laboratory Desktop Software, version 3.3) equipped with a platinum plate.

2. Supplementary Methods

2.1 Ligand Synthesis

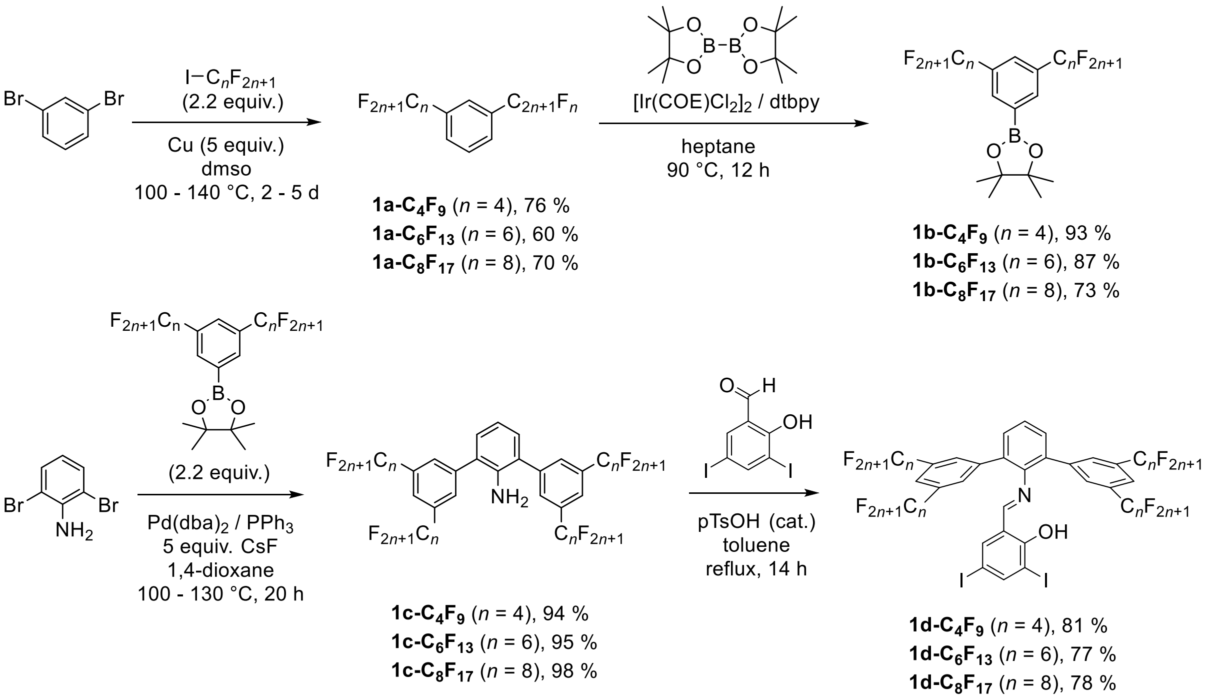

Supplementary Figure 1. Synthesis scheme of salicylaldimine ligands with perfluoroalkyl substitution. Starting with the synthesis of 1,3-(perfluoroalkyl)benzene, followed by selective borylation and Suzuki coupling with 2,6-dibromobenzene gave the respective terphenylamines. The salicylaldimine was obtained by acid catalyzed condensation with 3,5-diiodosalicylaldehyde.

Synthesis of 1,3-di(perfluoroalkyl)benzenes (**1a**)

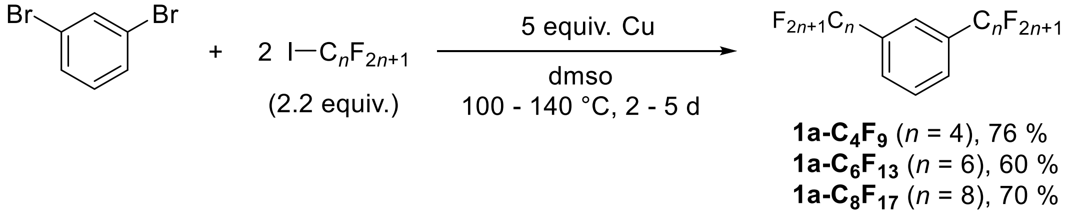

Supplementary Figure 2. Synthesis of 1,3-Di(perfluoroalkyl)benzenes (**1a**). The compounds were obtained by Copper-promoted Ullmann coupling of 1,3-dibromobenzene with the appropriate linear perfluoroalkyliodide.

General procedure:

Compounds **1a** were synthesized according to a modified literature procedure.[4] 1,3-dibromobenzene (1 equiv.), copper powder (5 equiv.) and perfluoroalkyliodide (2.2 equiv.) were added to 250 mL of dmso. The reaction mixture was sealed and stirred for 2 – 5 days at 100 – 140 °C under exclusion of light. The resulting red suspension was allowed to cool to room temperature, poured into 400 mL of water and stirred for 15 minutes. The red precipitates (inorganic salts) were filtered off and washed with diethyl ether (2 x 150 mL). The aqueous filtrate was extracted with diethyl ether (3 x 100 mL). The combined organic phases were

washed with water (2 x 100 mL), brine (100 mL) and dried over sodium sulfate. After removing the solvent under reduced pressure the product was obtained as yellow oil.

1,3-Diperfluorobutylbenzene (**1a-C_4F_9**)

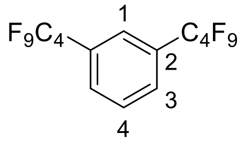

M = 514.2 g/mol

1a-C_4F_9, yield: 76 %

Compound **1a-C_4F_9** was synthesized according to the above general procedure using 7.75 g 1,3-dibromobenzene (32.8 mmol, 1 equiv.), 10.42 g copper powder (164 mmol, 5 equiv.) and 24.98 g perfluorobutyliodide (72.2 mmol, 2.2 equiv.). The reaction mixture was kept at 100 °C for 2 days. Yield: 76 %, 28.2 g, 54.8 mmol

^{1}H NMR (400 MHz, $CDCl_3$, 300 K): δ (ppm) = 7.87 – 7.79 (m, 3H, *H-1*, *H-3*), 7.70 (t, $^3J_{HH}$ = 7.6 Hz, *H-2*).

^{19}F{^{1}H} NMR (376 MHz, $CDCl_3$, 300 K): δ (ppm) = -81.1 (tt, J_{FF} = 10 Hz, J_{FF} = 3 Hz, 6F, -*CF_3*), -111.4 (t, $^3J_{FF}$ = 14 Hz, 4F, C_{arom}*CF_2*), -122.8 (m, 4F, *CF_2*), -125.6 (m, 4F, *CF_2*).

^{13}C{^{1}H} NMR (101 MHz, $CDCl_3$, 300 K): δ (ppm) = 130.6 (t, $^3J_{CF}$ = 7 Hz, *C-3*), 130.1 (t, $^2J_{CF}$ = 25 Hz, *C-2*), 129.5 (s, *C-4*), 125.7 (quint., $^3J_{CF}$ = 7 Hz, *C-1*) , 125-107 (*-C_4F_9*, *broad due to multiple $^XJ_{CF}$ couplings)*

1,3-Diperfluorohexylbenzene (**1a-C_6F_{13}**)

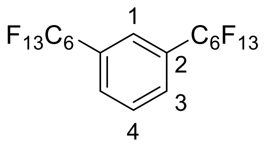

M = 714.2 g/mol

1a-C_6F_{13}, yield: 60 %

Compound **1a-C_6F_{13}** was synthesized according to the above general procedure using 7.61 g 1,3-dibromobenzene (32.3 mmol, 1 equiv.), 10.26 g copper powder (161.5 mmol, 5 equiv.) and 31.71 g perfluorohexyliodide (71.1 mol, 2.2 equiv.). The reaction mixture was kept at 140 °C for 5 days. Yield: 60 %, 13.5 g, 18.9 mmol

^{1}H NMR (400 MHz, $CDCl_3$/ C_6F_6, 300 K): δ (ppm) = 7.85 – 7.79 (m, 3H, *H-1, H-3*), 7.68 (t, $^3J_{HH}$ = 3.5 Hz, *H-4*).

$^{19}F\{^1H\}$ NMR (376 MHz, $CDCl_3$/ C_6F_6, 300 K): δ (ppm) = -81.7 (tt, J_{FF} = 10 Hz, J_{FF} = 3 Hz, 6F, *CF_3*), -111.8 (t, J_{FF} = 15 Hz, 4F, C_{arom}*CF_2*), -121.9 (m, 4F, *CF_2*), -122.4 (m, 4F, *CF_2*), -123.3 (m, 4F, *CF_2*), -126.8 (m, 4F, *CF_2*).

$^{13}C\{^1H\}$ NMR (101 MHz, $CDCl_3$, 300 K): δ (ppm) = 130.6 (t, $^4J_{CF}$ = 6 Hz, *C-3*), 130.5 (t, $^3J_{CF}$ = 25 Hz, *C-2*), 129.5 (s, *C-4*), 125.8 (t, $^4J_{CF}$ = 6 Hz, *C-1*), 120-150 (*-C_6F_{13}*, *broad due to multiple XJCF couplings*)

1,3-Diperfluorooctylbenzene (**1a-C_8F_{17}**)

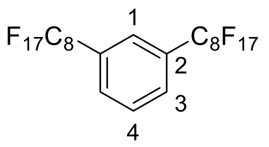

M = 914.2 g/mol

1a-C_8F_{17}, yield: 70 %

Compound **1a-C_8F_{17}** was synthesized according to the above general procedure using 1.42 g 1,3-dibromobenzene (6 mmol, 1 equiv.), 1.97 g copper powder (30 mmol, 5 equiv.) and 7.21 g perfluorooctyliodide (13.2 mmol, 2.2 equiv.). The reaction mixture was kept at 140 °C for 5 days. Yield: 70 %, 3.84 g, 4.2 mmol

^1H NMR (400 MHz, $CDCl_3$/ C_6F_6, 300 K): δ (ppm) = 7.88-7.78 (m, 2H, *H-1, H-3*), 7.71 (t, $^3J_{HH}$ = 7.9 Hz, 1H, *H-3*).

$^{19}F\{^1H\}$ NMR (376 MHz, $CDCl_3$/ C_6F_6, 300 K): δ (ppm) = -81.2 (m, 6F, *CF_3*), -111.5 (t, 4F, C_{arom}*CF_2*), -121.4 (m, 4F, *CF_2*), -122.2 (m, 12F, *CF_2*), -123.0 (m, 4F, *CF_2*), -126.4 (m, 4F, *CF_2*).

$^{13}C\{^1H\}$ NMR (101 MHz, $CDCl_3$/ C_6F_6, 300 K): δ (ppm) = 130.6 (m, *C-3*), 129.5 (s, *C-4*), 130.3 (t, $^2J_{CF}$ = 25 Hz, *C-2*), 125.8 (m, *C-1*).

<u>Synthesis of 3,5-diperfluoroalkylphenylboronic acid pinacol esters (**1b**)</u>

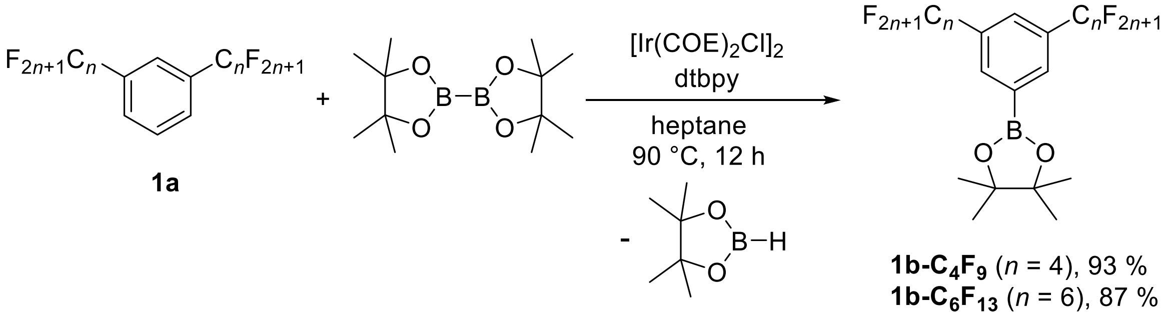

1b-C_4F_9 (*n* = 4), 93 %
1b-C_6F_{13} (*n* = 6), 87 %
1b-C_8F_{17} (*n* = 8), 73 %

Supplementary Figure 3. Synthesis of 3,5-Diperfluoroalkylphenylboronic acid pinacol esters (**1b**). The selective borylation was performed via iridium catalyzed reaction with bis(pinacolato)diboron.

General procedure:

The synthesis was conducted according to a modified literature procedure.[5] $[Ir(COE)_2Cl]_2$ (0.005 - 0.01 equiv.) and 4,4'-di-tert-butyl-2,2'-dipyridyl (0.01 - 0.02 equiv.) were stirred in 5 mL of heptane to give a dark blue catalyst suspension. This was transferred to a mixture of 1,3-diperfluoroalkylbenzene (**1a**, 1 equiv.) and bis(bipinacolato)biboron (1 equiv.) in 150 mL of heptane and stirred for 12 hours at 90 °C to give an orange solution. The reaction mixture was allowed to cool to room temperature, extracted with brine (2 x 150 mL) and dried over sodium sulfate. After removing the solvent in vacuum, the product was obtained as yellow oil. This was kept under vacuum for several hours to remove residues of pinacolborane.

3,5-Diperfluorobutylphenylboronic acid pinacol ester (**1b-C_4F_9**)

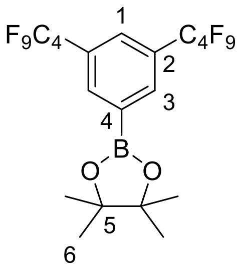

M = 640.1 g/mol

1b-C_4F_9, yield: 93 %

Compound **1b-C_4F_9** was synthesized according to the above general procedure using 25.7 g 1,3-diperfluorobutylbenzene (**1a-C_4F_9**, 50 mmol, 1 equiv.), 12.7 g bis(pinacolato)diboron (50 mmol, 1 equiv.), 0.180 g $[Ir(COE)_2Cl]_2$ (0.005 equiv.) and 0.125 g 4,4'-Di-tert-butyl-2,2'-dipyridyl (0.01 equiv.). Yield: 93 %, 29.8 g, 47 mmol

^{1}H NMR (400 MHz, $CDCl_3$, 300 K): δ (ppm) = 8.23 (s, 2H, *H-3*), 7.87 (s, 1H, *H-1*), 1.37 (s, 12H, *H-6*).

^{19}F{^{1}H} NMR (376 MHz, $CDCl_3$, 300 K): δ (ppm) = -81.4 (tt, J_{FF} = 10 Hz, J_{FF} = 3 Hz, 6F, *CF_3*), -111.5 (m, 4F, C_{arom}*CF_2*), -122.7 (m, 4F, *CF_2*), -125.9 (m, 4F, *CF_2*).

^{13}C{^{1}H} NMR (101 MHz, $CDCl_3$, 300 K): δ (ppm) = 136.7 (t, $^3J_{CF}$ = 6 Hz, *C-3*), 131.6 (br. m, *C-4*), 129.6 (t, $^3J_{CF}$ = 25 Hz, *C-2*), 128.0 (quint., $^3J_{CF}$ = 6 Hz, *C-1*), 123-106 (*-C_4F_9, broad due to multiple $^XJ_{CF}$ couplings),* 85.1 (s, *C-5*), 24.9 (s, *C-6*).

3,5-Diperfluorohexylphenylboronic acid pinacol ester (**1b-C_6F_{13}**)

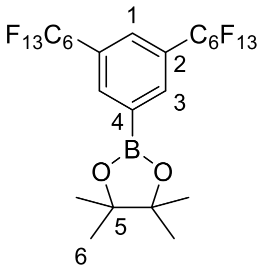

M = 840.2 g/mol

B-C_6F_{13}, yield: 87 %

Compound **1b-C_6F_{13}** was synthesized according to the above general procedure using 10.1 g 1,3-diperfluorohexyllbenzene (**1a-C_6F_{13},** 14.1 mmol, 1 equiv.), 3.6 g bis(pinacolato)diboron (14.1 mmol, 1 equiv.), 63 mg $[Ir(COE)_2Cl]_2$ (0.005 equiv.) and 50 mg 4,4'-Di-tert-butyl-2,2'-dipyridyl (0.01 equiv.). Yield: 87 %, 10.4 g, 47 mmol

^{1}H NMR (400 MHz, $CDCl_3$, 300 K): δ (ppm) = 8.22 (s, 2H, *H-3*), 7.86 (s, 1H, *H-1*), 1.37 (s, 12H, *H-6*).

^{19}F{^{1}H} NMR (376 MHz, $CDCl_3$, 300 K): δ (ppm) = -80.9 (tt, J_{FF} = 10 Hz, J_{FF} = 2 Hz, 6F, *CF_3*), -111.0 (t, J_{FF} = 14 Hz, 4F, C_{arom}*CF_2*), -121.3 to -121.8 (m, 8F, *CF_2*), -122.9 (m, 4F, *CF_2*), -126.2 (m, 4F, *CF_2*).

^{13}C{^{1}H} NMR (101 MHz, $CDCl_3$, 300 K): δ (ppm) = 136.6 (m, *C-3*), 129.5 (t, $^3J_{CF}$ = 25 Hz, *C-2*), 128.0 (m, *C-1*), 122-104 (*-C_6F_{13}, broad due to multiple $^XJ_{CF}$ couplings*), 85.0 (s, *C-5*), 25.0 (s, *C-6*), *C-4 was not detected, as expected for quaternary carbons with attached boryl groups.*

3,5-Diperfluorooctylphenylboronic acid pinacol ester (**1b-C_8F_{17}**)

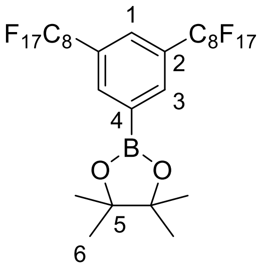

M = 1040.2 g/mol

1b-C_8F_{17}, yield: 73 %

Compound **1b-C_8F_{17}** was synthesized according to the above general procedure using 3.16 g 1,3-diperfluorooctylbenzene (**1a-C_8F_{17}**, 3.45 mmol, 1 equiv.), 0.88 g bis(pinacolato)diboron (3.45 mmol, 1 equiv.), 31 mg $[Ir(COE)_2Cl]_2$ (0.01 equiv.) and 25 mg 4,4'-Di-tert-butyl-2,2'-dipyridyl (0.02 equiv.). Yield: 73 %, 2.5 g, 47 mmol

^{1}H NMR (400 MHz, $CDCl_3$, 300 K): δ (ppm) = 8.23 (s, 2H, *H-3*), 7.86 (s, 1H, *H-1*), 1.37 (s, 12H, *H-6*).

^{19}F{^{1}H} NMR (376 MHz, $CDCl_3$, 300 K): δ (ppm) = -80.9 (tt, J_{FF} = 11 Hz, J_{FF} = 2 Hz, 6F, *CF_3*), -111.1 (t, J_{FF} = 14 Hz, 4F, C_{arom}*CF_2*), -121.2 (m, 4F, *CF_2*), -121.5 to -122.3 (m, 12F, *CF_2*), -122.8 (m, 4F, *CF_2*), -126.2 (m, 4F, *CF_2*).

^{13}C{^{1}H} NMR (101 MHz, $CDCl_3$, 300 K): δ (ppm) = 136.6 (t, $^{3}J_{CF}$ = 6 Hz, *C-3*), 129.5 (t, $^{3}J_{CF}$ = 25 Hz, *C-2*), 128.0 (m, *C-1*), 119-104 (*-C_8F_{17}, broad due to multiple $^{X}J_{CF}$ couplings),* 85.0 (s, *C-5*), 25.0 (s, *C-6*), *C-4 was not detected, as expected for quaternary carbons with attached boryl groups.*

Synthesis of 3,3',5,5'-tetra(perfluoroalkyl)terphenyl amines (**1c**)

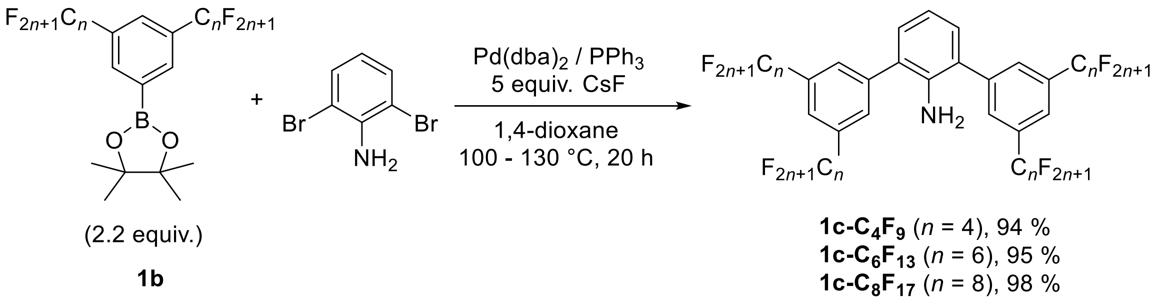

Supplementary Figure 4. Synthesis of 3,3',5,5'-Tetra(perfluoroalkyl)terphenyl amines (**1c**). The terphenylamines were obtained from palladium catalyzed Suzuki coupling of boronic esters with 2,6-dibromoaniline.

General procedure:

[Pd(dba)$_2$] and triphenylphosphine were stirred in 10 mL of dioxane to give an orange catalyst solution. This was added to a mixture of 3,5-diperfluorohexylphenylboronic acid pinacol ester (**2**, 2.2 equiv.), 2,6-dibromoaniline (1 equiv.) and cesium fluoride (5 equiv.) in 300 mL of dioxane. The dark orange reaction mixture was heated to 130 °C for 2 hours, and stirred at 100 °C overnight to give a green suspension. The solvent was removed under reduced pressure, and the residue (dark sticky oil and inorganic salts) was washed with warm water (2 x 250 mL) and warm methanol (2 x 250 mL). The dark residue was diluted in diethyl ether and filtrated over a plug of celite to remove palladium black impurities. After drying over sodium sulfate, the solvent was removed under reduced pressure to give the product as a brownish wax. The products were used for the next step (condensation) without further purification as the showed only minor impurities in the NMR spectra. Pure products could be obtained by column chromatography on silica using pentane/triethylamine [1:0.005] (for *n* = 6 and 8) or pentane/diethyl ether/trimethylamine [1:0.05:0.005] (for *n* = 4) as eluent.

3,3',5,5'-Tetra(perfluorobutyl)terphenyl amine (**1c-C_4F_9**)

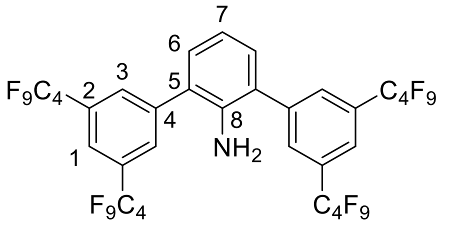

M = 1117.4 g/mol

1c-C_4F_9, yield: 94 %

Compound **1c-C_4F_9** was synthesized according to the above general procedure using 10.0 g 3,5-diperfluorobutyllphenylboronic acid pinacol ester (**1b-C_4F_9**, 15.6 mmol, 2.2 equiv.), 1.78 g 2,6-dibromoaniline (7.1 mmol, 1 equiv.), 5.36 g cesium fluoride (35.3 mmol, 5 equiv.), 0.208 g Pd(dba)$_2$ (0.36 mmol, 0.05 equiv.) and 0.209 g triphenylphosphine (0.8 mmol, 0.11 equiv.). Yield: 94 %, 7.46 g, 6.7 mmol

^{1}H NMR (400 MHz, $CDCl_3$, 300 K): δ (ppm) = 7.99 (s, 4H, *H-3*), 7.82 (s, 2H, *H-1*), 7.19 (d, $^3J_{HH}$ = 7.6 Hz, 2H, *H-6*), 7.00 (t, $^3J_{HH}$ = 7.6 Hz, 1H, *H-7*), 3.68 (s, 2H, *NH_2*).

^{19}F{^{1}H} NMR (376 MHz, $CDCl_3$, 300 K): δ (ppm) = -81.1 (tt, J_{FF} = 10 Hz, J_{FF} = 3 Hz, 12F, *CF_3*), -111.3 (t, J_{FF} = 12 Hz, 8F, C_{arom}*CF_2*), -122.6 (m, 8F, *CF_2*), -125.6 (m, 8F, *CF_2*).

^{13}C{^{1}H} NMR (101 MHz, $CDCl_3$, 300 K): δ (ppm) = 141.2 (s, *C-8*), 140.6 (s, *C-4*), 131.6 (m, *C-3*), 131.4 (s, *C-6*), 131.0 (t, $^2J_{CF}$ = 25 Hz, *C-2*), 125.5 (s, *C-5*), 124.7 (m, *C-1*), 121-105 (*-C_4F_9, broad due to multiple $^XJ_{CF}$ couplings*), 119.5 (s, *C-7*).

3,3',5,5'-Tetra(perfluorohexl)terphenyl amine (**1c-C_6F_{13}**)

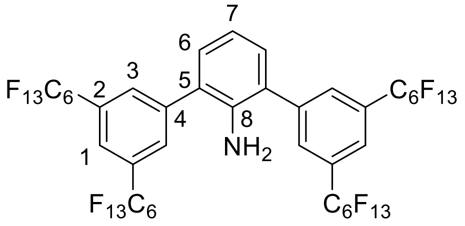

M = 1517.5 g/mol

1c-C_6F_{13}, yield: 95 %

Compound **1c-C_6F_{13}** was synthesized according to the above general procedure using 13.41 g 3,5-diperfluorohexylphenylboronic acid pinacol ester (**1b-C_6F_{13}**, 15.7 mmol, 2.2 equiv.), 1.82 g 2,6-dibromoaniline (7.25 mmol, 1 equiv.), 5.51 g cesium fluoride (36.3 mmol, 5 equiv.), 0.208 g Pd(dba)$_2$ (0.36 mmol, 0.05 equiv.) and 0.209 g triphenylphosphine (0.8 mmol, 0.11 equiv.). Yield: 97 %, 10.67 g, 7 mmol

^{1}H NMR (400 MHz, $CDCl_3$/ C_6F_6, 300 K): δ (ppm) = 8.00 (s, 4H, *H-3*), 7.83 (s, 2H, *H-1*), 7.20 (d, $^3J_{HH}$ = 7.7 Hz, 2H, *H-6*), 6.99 (t, $^3J_{HH}$ = 7.7 Hz, 1H, *H-7*), 3.68 (s, 2H, *NH_2*).

^{19}F{^{1}H} NMR (376 MHz, $CDCl_3$/ C_6F_6, 300 K): δ (ppm) = -81.1 (tt, J_{FF} = 10 Hz, J_{FF} = 3 Hz, 12F, *CF_3*), -111.2 (t, J_{FF} = 12 Hz, 8F, C_{arom}*CF_2*), -121.5 (m, 8F, *CF_2*), -121.9 (m, 8F, *CF_2*), -123.0 (m, 8F, *CF_2*), -126.4 (m, 8F, *CF_2*).

^{13}C{^{1}H} NMR (101 MHz, $CDCl_3$/ C_6F_6, 300 K): δ (ppm) = 141.2 (s, *C-8*), 140.7 (s, *C-4*), 131.6 (m, *C-3*), 131.3 (s, *C-6*), 131.1 (t, $^2J_{CF}$ = 25 Hz, *C-2*), 125.5 (s, *C-5*), 124.8 (m, *C-1*), 120-105 (*-C_6F_{13}, broad due to multiple $^XJ_{CF}$ couplings*), 119.5 (s, *C-7*).

3,3',5,5'-Tetra(perfluorooctyl)terphenyl amine (**1c-C_8F_{17}**)

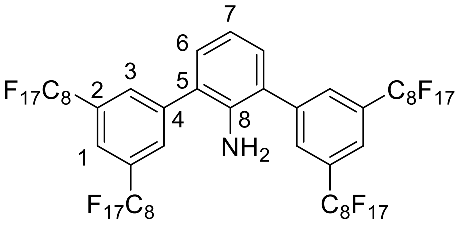

M = 1917.5 g/mol

1c-C_8F_{17}, yield: 98 %

Compound **1c-C_8F_{17}** was synthesized according to the above general procedure using 10.4 g 3,5-diperfluoroocylphenylboronic acid pinacol ester (**1b-C_8F_{17}**10 mmol, 2.2 equiv.), 1.09 g 2,6-dibromoaniline (4.35 mmol, 1 equiv.), 3.04 g cesium fluoride (20 mmol, 4.6 equiv.), 0.127 g $Pd(dba)_2$ (0.22 mmol, 0.05 equiv.) and 0.126 g triphenylphosphine (0.48 mmol, 0.11 equiv.). Yield: 98 %, 8.16 g, 4.3 mmol

^{1}H NMR (400 MHz, $CDCl_3$/ C_6F_6, 300 K): δ (ppm) = 8.03 (s, 4H, *H-3*), 7.85 (s, 2H, *H-1*), 7.22 (d, $^3J_{HH}$ = 7.6 Hz, 2H, *H-6*), 7.00 (t, $^3J_{HH}$ = 7.6 Hz, 1H, *H-7*), 3.73 (s, 2H, *NH_2*).

^{19}F{^{1}H} NMR (376 MHz, $CDCl_3$/ C_6F_6, 300 K): δ (ppm) = -80.9 (tt, J_{FF} = 10 Hz, J_{FF} = 2 Hz, 12F, *CF_3*), -111.1 (t, J_{FF} = 14 Hz, 8F, C_{arom}*CF_2*), -121.2 (m, 8F, *CF_2*), -121.6 to -122.3 (m, 24F, *CF_2*), -122.8 (m, 8F, *CF_2*), -126.2 (m, 8F, *CF_2*).

^{13}C{^{1}H} NMR (101 MHz, $CDCl_3$/ C_6F_6, 300 K): δ (ppm) = 141.4 (s, *C-8*), 140.8 (s, *C-4*), 131.7 (m, *C-3*), 131.4 (s, *C-6*), 131.3 (t, $^2J_{CF}$ = 25 Hz, *C-2*), 125.7 (s, *C-5*), 124.7 (m, *C-1*), 121-105 (*-C_8F_{17}, broad due to multiple $^XJ_{CF}$ couplings*), 119.5 (s, *C-7*).

Synthesis of 3,5-diiodo-N-[2,6-bis(3,5-diperfluoroalkyphenyl)phenyl]-salicylaldimines (**1d**)

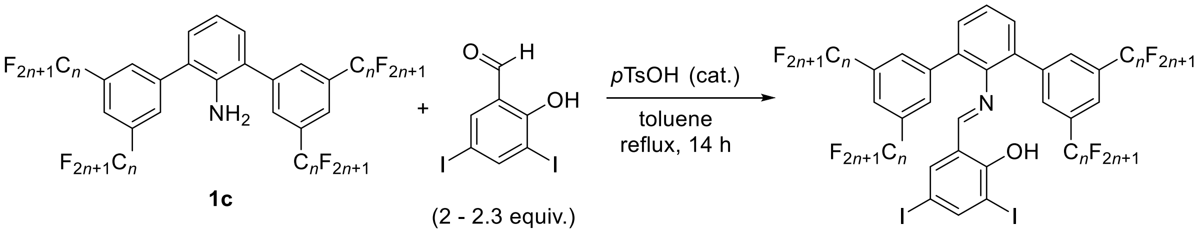

1d-C_4F_9 (n = 4), 81 %
1d-C_6F_{13} (n = 6), 77 %
1d-C_8F_{17} (n = 8), 78 %

Supplementary Figure 5. Synthesis of 3,5-Diiodo-N-[2,6-bis(3,5-diperfluoroalkyphenyl)phenyl]-salicylaldimines (**1d**). Highly pure salicylaldimines were obtained from acid catalyzed condensation of terphenylamines with 3,5-diiodosalicylaldhyde, followed by column chromatography (pentane as eluent).

General procedure:

Terphenylamine (**1c**, 1 equiv.), 3,5-diiodosalicylaldehyde (2.0 – 2.3 equiv.) and 50 mg *p*-toluene sulfonic acid hydrate (*p*TsOH) were added to 200 – 250 mL of toluene. The flask was equipped with a Soxhlet apparatus filled with dried molecular sieves to allow an azeotropic water removal during the reaction. The reaction mixture was heated to intense reflux (heating bath temperature >160 °C) for 14 hours. Note, that a complete dissolution of the terphenylamine is essential for conversion to the desired product. The reaction mixture was allowed to cool down to room temperature, and the solvent removed was under reduced pressure. After purification via column chromatography on silica with pentane as eluent (yellow band), the product was obtained as a yellow sticky oil. Precipitation from a saturated pentane solution at -78 °C gave the pure product as a yellow powder.

3,5-Diiodo-N-[2,6-bis(3,5-diperfluorobutylphenyl)phenyl]-salicylaldimine (**1d-C_4F_9**)

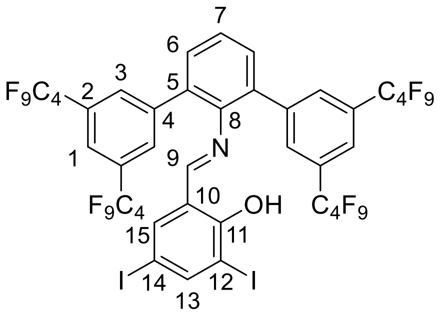

M = 1473.3 g/mol

1d-C_4F_9, yield: 81 %

Compound **1d-C_4F_9** was obtained following the general procedure with 7.46 g 3,3',5,5'-tetra(perfluorobutyl)terphenyl amine (**1c-C_4F_9**, 6.7 mmol, 1 equiv.), 5.0 g 3,5-diiodosalicylaldehyde (13.4 mmol, 2 equiv.) and 50 mg *p*TsOH in 250 mL of toluene. Yield: 81 %, 8.0 g, 5.4 mmol.

^{1}H NMR (400 MHz, $CDCl_3$, 300 K): δ (ppm) = 12.65 (s, 1H, O*H*), 8.01 (d, $^{4}J_{HH}$ = 2.0 Hz, *H-13*), 7.78 (br s, 4H, *H-3, H-9*), 7.75 (s, 2H, *H-1*), 7.52 (m, 3H, *H-6*, *H-7*), 7.04 (d, $^{4}J_{HH}$ = 2.1 Hz, *H-15*).

^{19}F{^{1}H} NMR (376 MHz, $CDCl_3$, 300 K): δ (ppm) = -80.9 (tt, J_{FF} = 11 Hz, J_{FF} = 3 Hz, 12F, *CF_3*), -111.3 (t, J_{FF} = 14 Hz, 8F, C_{arom}*CF_2*), -122.7 (m, 8F, *CF_2*), -125.5 (m, 8F, *CF_2*).

^{13}C{^{1}H} NMR (101 MHz, $CDCl_3$, 300 K): δ (ppm) = 168.3 (s, *C-9*), 159.8 (s, *C-11*), 150.4 (s, *C-13*), 144.7 (s, *C-8*), 140.6 (s, *C-4*), 140.3 (s, *C-15*), 132.3 (s, *C-5*), 131.8 (m, *C-3*, *C-6*), 130.6 (t, $^{2}J_{CF}$ = 25 Hz, *C-2*), 127.3 (s, *C-7*), 124.7 (m, *C-1*), 119-105 (*-C_4F_9*, *broad due to multiple $^{X}J_{CF}$ couplings*), 119.3 (s, *C-10*), 86.7 (s, *C-12*), 79.8 (s, *C-14*).

Elemental analysis (%) for $C_{41}H_{13}F_{36}I_2NO$: Found (Calculated)
C 33.63 (33.42); H 1.49 (0.89); N 1.19 (0.95)

3,5-Diiodo-N-[2,6-bis(3,5-diperfluorohexylphenyl)phenyl]-salicylaldimine (**1d-C_6F_{13}**)

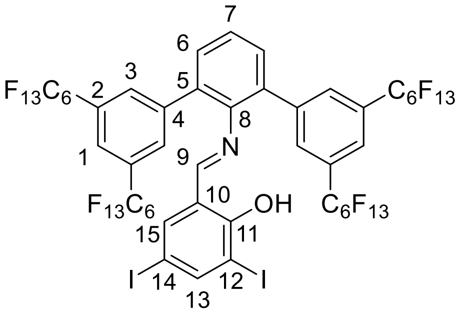

M = 1873.4 g/mol

1d-C_6F_{13}, yield: 77 %

Compound **1d-C_6F_{13}** was obtained following the general procedure with 8.8 g 3,3',5,5'-tetra(perfluorohexl)terphenyl amine (**1c-C_6F_{13}**, 5.6 mmol, 1 equiv.), 4.2 g 3,5-diiodosalicylaldehyde (11.2 mmol, 2 equiv.) and 50 mg *p*TsOH in 250 mL of toluene. Yield: 77 %, 8.1 g, 4.3 mmol.

^{1}H NMR (400 MHz, $CDCl_3$, 300 K): δ (ppm) = 12.68 (s, 1H, O*H*), 8.00 (d, $^{4}J_{HH}$ = 2.0 Hz, *H-13*), 7.79 (br s, 4H, *H-3, H-9*), 7.75 (s, 2H, *H-1*), 7.52 (m, 3H, *H-6*, *H-7*), 7.04 (d, $^{4}J_{HH}$ = 2.0 Hz, *H-15*).

^{19}F{^{1}H} NMR (376 MHz, $CDCl_3$/ C_6F_6, 300 K): δ (ppm) = -80.8 (tt, J_{FF} = 10 Hz, J_{FF} = 3 Hz, 12F, *CF_3*), -111.3 (t, J_{FF} = 12 Hz, 8F, C_{arom}*CF_2*), -121.5 (m, 8F, *CF_2*), -121.7 (m, 8F, *CF_2*), -122.7 (m, 8F, *CF_2*), -126.1 (m, 8F, *CF_2*).

^{13}C{^{1}H} NMR (101 MHz, $CDCl_3$, 300 K): δ (ppm) = 168.2 (s, *C-9*), 159.9 (s, *C-11*), 150.5 (s, *C-13*), 144.7 (s, *C-8*), 140.6 (s, *C-4*), 140.2 (s, *C-15*), 132.3 (s, *C-5*), 131.8 (m, *C-3*, *C-6*), 130.7

(t, $^2J_{CF}$ = 25 Hz, *C-2*), 127.4 (s, *C-7*), 124.7 (quint., $^3J_{CF}$ = 6.6 Hz, *C-1*), 122-105 (*-C_6F_{13}, broad due to multiple $^XJ_{CF}$ couplings*), 119.3 (s, *C-10*), 86.7 (s, *C-12*), 79.8 (s, *C-14*).

Elemental analysis (%) for $C_{49}H_{13}F_{52}I_2NO$: Found (Calculated)
C 31.76 (31.42); H 1.10 (0.70); N 0.75 (0.79)

3,5-Diiodo-N-[2,6-bis(3,5-diperfluorooctylphenyl)phenyl]-salicylaldimine (**1d-C_8F_{17}**)

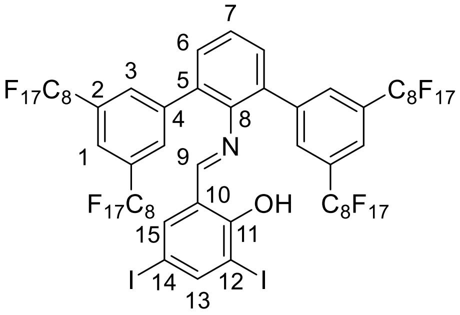

M = 2273.4 g/mol

1d-C_8F_{17}, yield: 81 %

Compound **1d-C_8F_{17}** was obtained following the general procedure with 4.39 g 3,3',5,5'-tetra(perfluorooctyl)terphenyl amine (**1c-C_8F_{17}**, 2.3 mmol, 1 equiv.), 1.94 g 3,5-diiodosalicylaldehyde (5.2 mmol, 2.3 equiv.) and 50 mg *p*TsOH in 200 mL of toluene. Yield: 81 %, 4.1 g, 1.79 mmol.

^{1}H NMR (400 MHz, $CDCl_3$, 300 K): δ (ppm) = 12.68 (s, 1H, O*H*), 8.00 (d, $^4J_{HH}$ = 2.0 Hz, *H-13*), 7.79 (br s, 4H, *H-3, H-9*), 7.75 (s, 2H, *H-1*), 7.52 (m, 3H, *H-6*, *H-7*), 7.04 (d, $^4J_{HH}$ = 2.0 Hz, *H-15*).

^{19}F{^{1}H} NMR (376 MHz, $CDCl_3$/ C_6F_6, 300 K): δ (ppm) = -80.9 (tt, J_{FF} = 10 Hz, J_{FF} = 2 Hz, 12F, *CF_3*), -111.1 (t, J_{FF} = 14 Hz, 8F, C_{arom}*CF_2*), -121.2 (m, 8F, *CF_2*), -121.6 to -122.3 (m, 24F, *CF_2*), -122.8 (m, 8F, *CF_2*), -126.2 (m, 8F, *CF_2*).

^{13}C{^{1}H} NMR (101 MHz, $CDCl_3$, 300 K): δ (ppm) = 168.2 (s, *C-9*), 159.9 (s, *C-11*), 150.5 (s, *C-13*), 144.7 (s, *C-8*), 140.6 (s, *C-4*), 140.2 (s, *C-15*), 132.3 (s, *C-5*), 131.8 (m, *C-3*, *C-6*), 130.7 (t, $^2J_{CF}$ = 25 Hz, *C-2*), 127.4 (s, *C-7*), 124.7 (quint., $^3J_{CF}$ = 6.6 Hz, *C-1*), 122-105 (*-C_8F_{17}, broad due to multiple $^XJ_{CF}$ couplings*), 119.3 (s, *C-10*), 86.7 (s, *C-12*), 79.8 (s, *C-14*).

Elemental analysis (%) for $C_{57}H_{13}F_{68}I_2NO$: Found (Calculated)
C 29.88 (30.11); H 1.10 (0.58); N 0.58 (0.62)

2.4 Synthesis of complexes

Synthesis of *κ²-(N,O)*-Salicylaldiminato NickelI(II) Methyl Pyridine Complexes (**1/Pyr**)

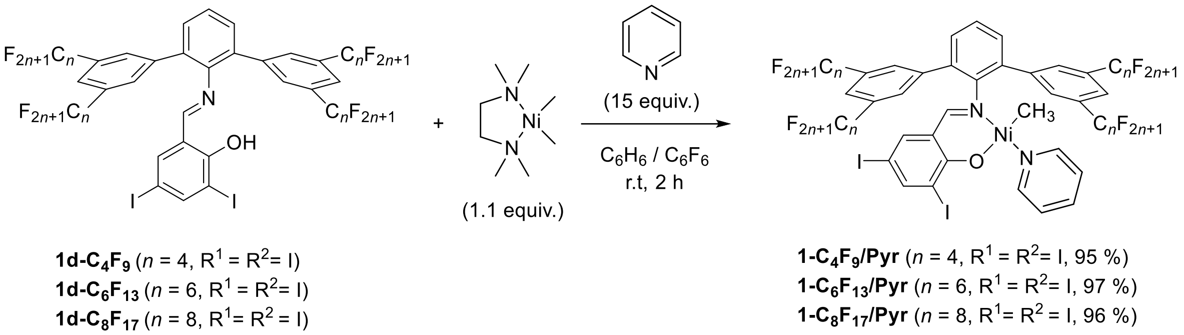

Supplementary Figure 6. Synthesis of κ²-(N,O)-Salicylaldiminato Nickel(II) Methyl Pyridine Complexes (**1/Pyr**). The labile ligand (pyridine) was added to a mixture of nickel precursor and salicylaldimine to generate the respective nickel complex bearing different fluorocarbon substituents.

General procedure:

To [(tmeda)NiMe$_2$] (1.1 equiv.) and the respective salicylaldimine (**1d**, 1 equiv.), a solution of pyridine (15 equiv.) in 4.5 - 7 mL of benzene/hexafluorobenzene was added. Gas evolution (methane) was observed and the reaction mixture turned orange to red. The reactants were stirred for 2 hours at room temperature. Now, the formed Nickel black was removed via centrifugation. The red solution was frozen in liquid nitrogen und the solvent removed by freeze drying to give the desired product as a red powder.

{3,5-Diiodo-N-[2,6-bis(3,5-diperfluorobutylphenyl)phenyl]salicylaldiminato- κ²-N,O}methylpyridinenickel(II) (**1-C$_4$F$_9$/Pyr**)

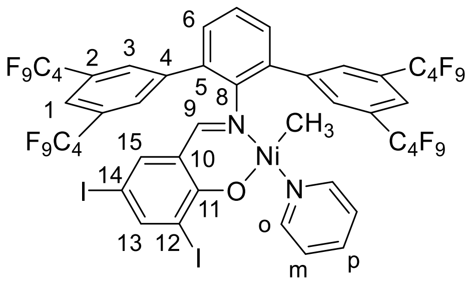

M = 1625.1 g/mol

1-C$_4$F$_9$ / Pyr, yield: 95 %

Complex **1-C$_4$F$_9$/Pyr** was synthesized following the general procedure for Ni(II) methyl pyridine complexes using 147 mg salicylaldimine **1d-C$_4$F$_9$** (100 µmol, 1 equiv.), 23 mg [(tmeda)NiMe$_2$] (110 µmol, 1.1 equiv.) and 119 mg pyridine (1.5 mmol, 15 equiv.) in 6 mL of benzene. Yield: 95 %, 95 µmol, 154 mg

^{1}**H** NMR (400 MHz, C_6D_6, 300 K): δ (ppm) = 8.38 (d, $^{3}J_{HH}$ = 5.6 Hz, 2H, *o-Pyr*), 8.27 (s, 4H, *H-3*), 8.03 (m, 3H, *H-1*, *H-13*), 7.06 (m, 3H, *H-6*, *H-7*), 6.73 (m, 3H, *H-15*, *m-Pyr*), 6.57 (s, 1H, *H-9*), 6.46 (t, $^{3}J_{HH}$ = 6.8 Hz, *p-Pyr*), -0.74 (s, 3H, Ni-*CH$_3$*).

^{19}**F{^{1}H}** NMR (376 MHz, C_6D_6, 300 K): δ (ppm) = -80.9 (t, J_{FF} = 10 Hz, 12F, *CF$_3$*), -110.9 (m, 8F, C_{arom}*CF$_2$*), -122.4 (m, 8F, *CF$_2$*), -125.4 (m, 8F, *CF$_2$*).

^{13}**C{^{1}H}** NMR (101 MHz, C_6D_6, 300 K): δ (ppm) = 167.8 (s, *C-9*), 164.1 (s, *C-11*), 151.2 (br. s, *o-Pyr*), 150.5 (s, *C-8*), 150.0 (s, *C-13*), 141.6 (s, *C-4*, *C-15*), 136.6 (br. s., *p-Pyr*), 132.6 (t, $^{3}J_{CF}$ = 5 Hz, *C-3*), 131.5 (s, *C-5*), 131.2 (s, *C-6*), 130.5 (t, $^{2}J_{CF}$ = 25 Hz, *C-2*), 127.1 (s, *C-7*), 124.6 (m, *C-1*), 123.2 (br. s, *m-Pyr*), 122-105 (*-C$_4$F$_9$, broad due to multiple $^{X}J_{CF}$ couplings*), 119.6 (s, *C-10*), 96.8 (s, *C-12*), 72.4 (s, *C-14*), -7.7 (s, Ni-*CH$_3$*).

Elemental analysis (%) for $C_{47}H_{20}F_{36}I_2N_2NiO$: Found (Calculated):
C 35.05 (34.74); H 1.82 (1.24); N 2.23 (1.72)

{3,5-Diiodo-N-[2,6-bis(3,5-diperfluorohexylphenyl)phenyl]salicylaldiminato- κ^2-N,O}methylpyridinenickel(II) (**1-C$_6$F$_{13}$/Pyr**)

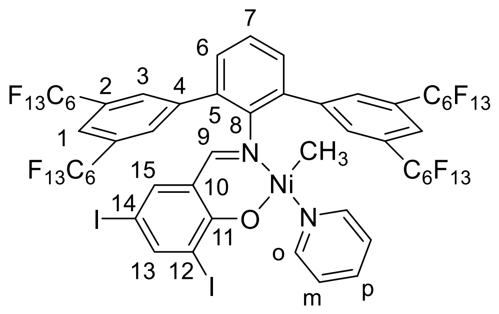

M = 2041.2 g/mol

1-C$_6$F$_{13}$ / Pyr, yield: 97 %

Complex **1-C$_6$F$_{13}$/Pyr** was synthesized following the general procedure for Ni(II) methyl pyridine complexes using 281 mg salicylaldimine **1d-C$_6$F$_{13}$** (150 µmol, 1 equiv.), 34 mg [(tmeda)NiMe$_2$] (165 µmol, 1.1 equiv.) and 178 mg pyridine (2.3 mmol, 15 equiv.) in 7 mL of benzene. Yield: 97 %, 146 µmol, 297 mg

^{1}**H** NMR (400 MHz, C_6D_6/C_6F_6, 300 K): δ (ppm) = 8.38 (d, $^{3}J_{HH}$ = 4.7 Hz, 2H, *o-Pyr*), 8.20 (s, 4H, *H-3*), 7.95 (m, 2H, *H-1*), 7.94 (d, 1H, $^{4}J_{HH}$ = 2.2 Hz, *H-13*), 6.97 (m, 3H, *H-6*, *H-7*), 6.70-6.58 (m, 3H, *H-15*, *m-Pyr*), 6.49 (s, 1H, *H-9*), 6.40 (t, $^{3}J_{HH}$ = 6.8 Hz, *p-Pyr*), -0.82 (s, 3H, Ni-*CH$_3$*).

^{19}**F{^{1}H}** NMR (376 MHz, C_6D_6/C_6F_6, 300 K): δ (ppm) = -81.5 (t, J_{FF} = 10 Hz, 12F, *CF$_3$*), -111.2 (m, 8F, C_{arom}*CF$_2$*), -121.6 (m, 16F, *CF$_2$*), -122.9 (m, 8F, *CF$_2$*), -126.4 (m, 8F, *CF$_2$*).

^{13}**C{^{1}H}** NMR (101 MHz, C_6D_6/C_6F_6, 300 K): δ (ppm) = 168.0 (s, *C-9*), 164.3 (s, *C-11*), 151.5 (br. s, *o-Pyr*), 150.8 (s, *C-8*), 150.3 (s, *C-13*), 141.8 (s, *C-4*), 141.7 (s, *C-15*), 133.8 (br. s., *m-*

Pyr), 132.8 (t, $^3J_{CF}$ = 5 Hz, *C-3*), 131.5 (s, *C-5*), 131.2 (s, *C-6*), 130.9 (t, $^2J_{CF}$ = 25 Hz, *C-2*), 127.3 (s, *C-7*), 124.9 (m, *C-1*), 123.4 (br. s, *p-Pyr*), 122-105 (*-C_6F_{13}, broad due to multiple $^XJ_{CF}$ couplings*), 119.8 (s, *C-10*), 96.7 (s, *C-12*), 72.1 (s, *C-14*), -8.0 (s, Ni-*CH_3*).

Elemental analysis (%) for $C_{55}H_{20}F_{52}I_2N_2NiO$: Found (Calculated)
C 32.49 (32.62); H 1.32 (1.00); N 1.38 (1.56)

{3,5-Diiodo-N-[2,6-bis(3,5-diperfluorooctylphenyl)phenyl]salicylaldiminato- κ^2-N,O}methylpyridinenickel(II) (**1-C_8F_{17}/Pyr**)

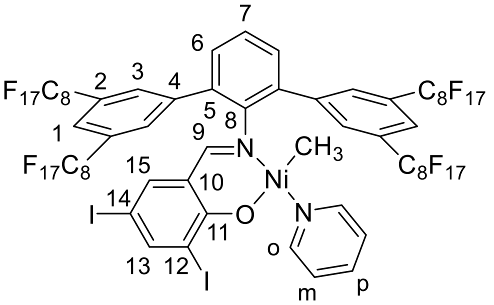

M = 2441.3 g/mol

1-C_8F_{17} / Pyr, yield: 96 %

Complex **1-C_8F_{17}/Pyr** was synthesized following the general procedure for Ni(II) methyl pyridine complexes using 341 mg salicylaldimine **1d-C_8F_{17}** (150 µmol, 1 equiv.), 34 mg [(tmeda)$NiMe_2$] (165 µmol, 1.1 equiv.) and 178 mg pyridine (2.3 mmol, 15 equiv.) in 5 mL of benzene and 1 mL of hexafluorobenzene. Yield: 96 %, 144 µmol, 369 mg.

^{1}H NMR (400 MHz, C_6D_6/C_6F_6, 300 K): δ (ppm) = 8.35 (d, $^3J_{HH}$ = 4.9 Hz, 2H, *o-Pyr*), 8.24 (s, 4H, *H-3*), 8.01 (m, 2H, *H-1*), 7.89 (d, 1H, $^4J_{HH}$ = 2.2 Hz, *H-13*), 7.05 (m, 3H, *H-6*, *H-7*), 6.76 (t, $^3J_{HH}$ = 7.7 Hz, 1H, *m-Pyr*), 6.65 (d, $^4J_{HH}$ = 2.2 Hz, 1H, *H-15*), 6.54 (s, 1H, H-9), 6.49 (t, $^3J_{HH}$ = 6.5 Hz, *p-Pyr*), -0.84 (s, 3H, Ni-*CH_3*).

^{19}F{^{1}H} NMR (376 MHz, C_6D_6/C_6F_6, 300 K): δ (ppm) = -81.4 (t, J_{FF} = 10 Hz, J_{FF} = 2 Hz, 12F, *CF_3*), -111.0 (t, J_{FF} = 14 Hz, 8F, C_{arom}*CF_2*), -121.0 to -122.5 (m, 32F, *CF_2*), -122.9 (m, 8F, *CF_2*), -126.4 (m, 8F, *CF_2*).

^{13}C{^{1}H} NMR (101 MHz, C_6D_6/C_6F_6, 300 K): δ (ppm) = 167.9 (s, *C-9*), 164.2 (s, *C-11*), 151.4 (s, *o-Pyr*), 150.7 (s, *C-8*), 150.3 (s, *C-13*), 141.7 (m, *C-4*, *C-15*), 133.3 (s, *m-Pyr*), 132.8 (t, $^3J_{CF}$ = 5 Hz, *C-3*), 131.3 (s, *C-5, C-6*), 130.7 (t, $^2J_{CF}$ = 25 Hz, *C-2*), 127.2 (s, *C-7*), 124.9 (m, *C-1*), 123.3 (s, *p-Pyr*), 122-105 (*-C_8F_{17}, broad due to multiple $^XJ_{CF}$ couplings*), 119.7 (s, *C-10*), 96.7 (s, *C-12*), 72.1 (s, *C-14*), -7.9 (s, Ni-*CH_3*).

Elemental analysis (%) for $C_{63}H_{20}F_{68}I_2N_2NiO$: Found (Calculated):
C 31.74 (31.20); H 1.59 (0.83); N 1.48 (1.16)

Synthesis of *κ²-(N,O)*-Salicylaldiminato NickelI(II) Methyl [α-Methoxy-ω-amino poly(ethylene glycol)] Complexes (**1/PEG**)

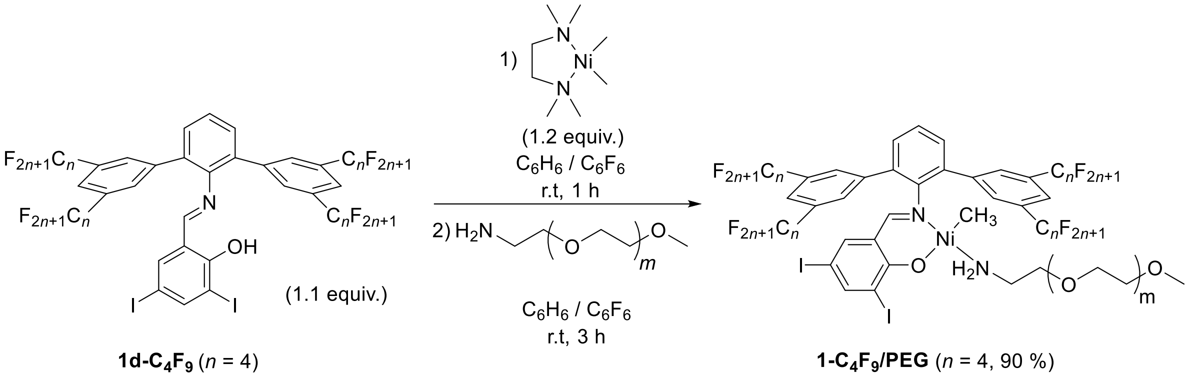

1d-C$_4$F$_9$ (*n* = 4)
1d-C$_6$F$_{13}$ (*n* = 6)
1d-C$_8$F$_{17}$ (*n* = 8)

1-C$_4$F$_9$/PEG (*n* = 4, 90 %)
1-C$_6$F$_{13}$/PEG (*n* = 6, 93 %)
1-C$_8$F$_{17}$/PEG (*n* = 8, 95 %)

Supplementary Figure 7. Synthesis of κ²-(N,O)-Salicylaldiminato Nickel(II) Methyl [α-Methoxy-ω-amino poly(ethylene glycol)] Complexes (**1/PEG**). The labile ligand (α-Methoxy-ω-amino poly[ethylene glycol]) was added to a reacted mixture of nickel precursor and salicylaldimine to generate the respective nickel complex bearing different fluorocarbon substituents.

General procedure:

To solid [(tmeda)NiMe$_2$] (1.2 equiv.) a solution of the respective salicylaldimine (**1d**, 1.1 equiv.) in a mixture of benzene/hexafluorobenzene or neat benzene was added, and stirred for 1 hour at room temperature. During the addition, gas evolution (methane) was observed and an orange to red solution (slightly turbid) was obtained. α-Methoxy-ω-amino poly(ethylene glycol) (1.0 equiv.; M_w = 1981 g mol^{-1}, unless noted otherwise) in 1 ml of benzene was added and the reaction was stirred for further 3 hours at room temperature (the solution cleared up). After filtration through a syringe filter to remove nickel black, the solvent was removed under vacuum. The orange residue was washed with portions of pentane (3-5 times, 7.5 mL each) until the filtrate remained almost colorless (slightly orange). After drying under vacuum, the desired product was obtained as an orange powder.

{3,5-Diiodo-N-[2,6-bis(3,5-diperfluorobutylphenyl)phenyl]salicylaldiminato- κ²-N,O}methyl[α-Methoxy-ω-amino poly(ethylene glycol)]nickel(II) (**1-C$_4$F$_9$/PEG**)

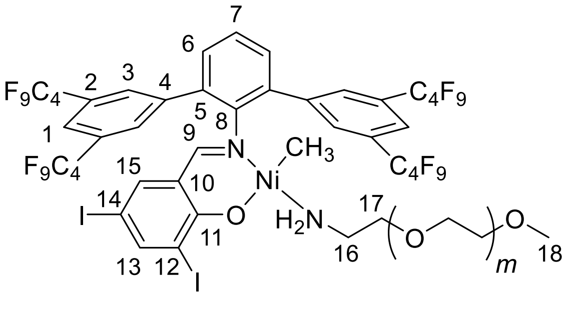

M = 3527 g/mol

1-C$_4$F$_9$/PEG, yield: 90 %

Complex **1-C_4F_9/PEG** was synthesized following the general procedure for Ni(II) methyl [α-Methoxy-ω-amino poly(ethylene glycol] complexes using 193 mg salicylaldimine **1d-C_4F_9** (131 µmol, 1.05 equiv.), 28 mg [(tmeda)$NiMe_2$] (138 µmol, 1.1 equiv.) and 248 mg [α-Methoxy-ω-amino poly(ethylene glycol] (125 µmol, 1 equiv.) in 6 mL of benzene. Yield: 90 %, 113 µmol, 399 mg

^{1}H NMR (400 MHz, C_6D_6, 300 K): δ (ppm) = 8.11 (s, 4H, *H-3*), 7.96 (d, 1H, $^4J_{HH}$ = 2.2 Hz, *H-13*), 7.90 (s, 2H, *H-1*), 6.95 (m, 3H, *H-6*, *H-7*), 6.63 (d, 1H, $^4J_{HH}$ = 2.2 Hz, *H-15*), 6.47 (s, 1H, *H-9*), 3.78-3.18 (m, 233H, *H*-PEG), 3.14 (s, 3H, *H-18*), 3.05 (t, 2H, $^3J_{HH}$ = 4.9 Hz, *H-17*), 2.50 (m, 2H, *H-16*), 1.06 (t, 2H, $^3J_{HH}$ = 7.1 Hz, *-NH_2*), -1.19 (s, 3H, Ni-*CH_3*).

^{19}F{^{1}H} NMR (376 MHz, C_6D_6, 300 K): δ (ppm) = -80.9 (t, J_{FF} = 10 Hz, 12F, *CF_3*), -110.9 (m, 8F, C_{arom}*CF_2*), -122.4 (m, 8F, *CF_2*), -125.4 (m, 8F, *CF_2*).

^{13}C{^{1}H} NMR (101 MHz, C_6D_6, 300 K): δ (ppm) = 167.3 (s, *C-9*), 163.1 (s, *C-11*), 150.3 (s, *C-8*), 149.7 (s, *C-13*), 141.6 (s, *C-15*), 141.5 (s, *C-4*), 133.2 (s, *C-5*), 132.5 (t, $^3J_{CF}$ = 5 Hz, *C-3*), 131.2 (s, *C-6*), 130.4 (t, $^2J_{CF}$ = 25 Hz, *C-2*), 127.1 (s, *C-7*), 124.6 (m, *C-1*), 122-105 (*-C_4F_9, broad due to multiple $^XJ_{CF}$ couplings*), 119.8 (s, *C-10*), 96.8 (s, *C-12*), 72.5-70.1 (m, *C*-PEG, *C-14, C-17*), 58.7 (s, *C-18*), 43.2 (s, *C-16*), -13.3 (s, Ni-*CH_3*).

Elemental analysis (%) for $C_{131}H_{196}F_{36}I_2N_2NiO_{45}$: Found (Calculated)
C 46.51 (44.76); H 6.19 (5.62); N 0.90 (0.80)

{3,5-Diiodo-N-[2,6-bis(3,5-diperfluorohexylphenyl)phenyl]salicylaldiminato- κ^2-N,O}methyl[α-Methoxy-ω-amino poly(ethylene glycol)]lnickel(II) (**1-C_6F_{13}/PEG**)

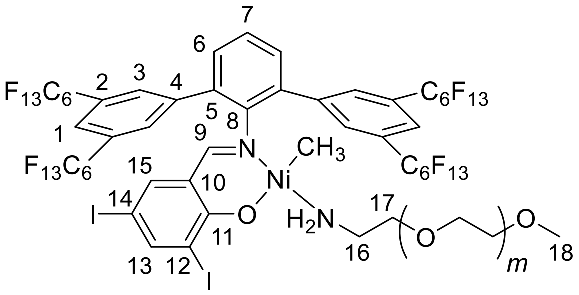

M = 3927 g/mol

1-C_6F_{13} / PEG, yield: 93 %

Complex **1-C_6F_{13}/PEG** was synthesized following the general procedure for Ni(II) methyl [α-Methoxy-ω-amino poly(ethylene glycol] complexes using 296 mg salicylaldimine **1d-C_6F_{13}** (158 µmol, 1.05 equiv.), 34 mg [(tmeda)$NiMe_2$] (165 µmol, 1.1 equiv.) and 297 mg [α-Methoxy-ω-amino poly(ethylene glycol] (150 µmol, 1 equiv.) in a mixture of 10 mL of benzene and 3 mL of hexafluorobenzene. Yield: 93 %, 140 µmol, 550 mg.
The analogue complex with M(H_2N-PEG-OMe) = 5516 g mol^{-1} was also prepared by an identical procedure.

^{1}H NMR (400 MHz, C_6D_6, 300 K): δ (ppm) = 8.14 (s, 4H, *H-3*), 7.97 (d, 1H, $^{4}J_{HH}$ = 2.2 Hz, *H-13*), 7.93 (s, 2H, *H-1*), 6.96 (m, 3H, *H-6*, *H-7*), 6.65 (d, 1H, $^{4}J_{HH}$ = 2.2 Hz, *H-15*), 6.50 (s, 1H, *H-9*), 3.78-3.19 (m, 230H, *H*-PEG), 3.14 (s, 3H, *H-18*), 3.07 (t, 2H, $^{3}J_{HH}$ = 4.9 Hz, *H-17*), 2.52 (m, 2H, *H-16*), 1.06 (t, 2H, $^{3}J_{HH}$ = 7.0 Hz, *-NH₂*), -1.16 (s, 3H, Ni-*CH₃*).

^{19}F{^{1}H} NMR (376 MHz, C_6D_6, 300 K): δ (ppm) = -80.9 (t, J_{FF} = 10 Hz, 12F, *CF_3*), -110.8 (t, 8F, J_{FF} = 14 Hz, C_{arom}*CF_2*), -121.4 (m, 16F, *CF_2*), -122.6 (m, 8F, *CF_2*), -126.0 (m, 8F, *CF_2*).

^{13}C{^{1}H} NMR (101 MHz, C_6D_6, 300 K): δ (ppm) = 167.2 (s, *C-9*), 163.1 (s, *C-11*), 150.2 (s, *C-8*), 149.8 (s, *C-13*), 141.6 (s, *C-15*), 141.5 (s, *C-4*), 133.2 (s, *C-5*), 132.5 (t, $^{3}J_{CF}$ = 5 Hz, *C-3*), 131.3 (s, *C-6*), 130.5 (t, $^{2}J_{CF}$ = 25 Hz, *C-2*), 127.1 (s, *C-7*), 124.7 (m, *C-1*), 122-105 (*-C_6F_{13}, broad due to multiple $^{X}J_{CF}$ couplings*), 119.8 (s, *C-10*), 96.8 (s, *C-12*), 72.5-70.1 (m, *C*-PEG, *C-14, C-17*), 58.7 (s, *C-18*), 43.2 (s, *C-16*), -13.3 (s, Ni-*CH_3*).

Elemental analysis (%) for $C_{139}H_{196}F_{52}I_2N_2NiO_{45}$: Found (Calculated)
C 43.31 (42.64); H 5.20 (5.05); N 0.82 (0.72)

{3,5-Diiodo-N-[2,6-bis(3,5-diperfluorooctylphenyl)phenyl]salicylaldiminato- κ²-N,O}methyl[α-Methoxy-ω-amino poly(ethylene glycol)]lnickel(II) (**1-C_8F_{17} /PEG**)

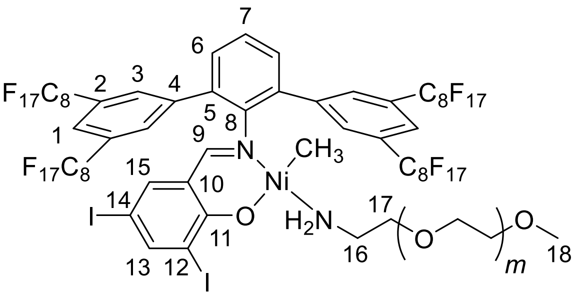

M = 4327 g/mol

1-C_8F_{17} / PEG, yield: 95 %

Complex **1-C_8F_{17}/PEG** was synthesized following the general procedure for Ni(II) methyl [α-Methoxy-ω-amino poly(ethylene glycol] complexes using 239 mg salicylaldimine **1d-C_8F_{17}** (105 µmol, 1.05 equiv.), 23 mg [(tmeda)$NiMe_2$] (110 µmol, 1.1 equiv.) and 196 mg [α-Methoxy-ω-amino poly(ethylene glycol] (100 µmol, 1 equiv.) in a mixture of 6 mL of benzene and 3 mL of hexafluorobenzene. Yield: 95 %, 95 µmol, 411 mg

^{1}H NMR (400 MHz, C_6D_6/C_6F_6, 300 K): δ (ppm) = 8.17 (s, 4H, *H-3*), 7.99 (d, 1H, $^{4}J_{HH}$ = 2.2 Hz, *H-13*), 7.96 (s, 2H, *H-1*), 6.98 (m, 3H, *H-6*, *H-7*), 6.66 (d, 1H, $^{4}J_{HH}$ = 2.2 Hz, *H-15*), 6.51 (s, 1H, *H-9*), 3.73-3.19 (m, 218H, *H*-PEG), 3.14 (s, 3H, *H-18*), 3.09 (t, 2H, $^{3}J_{HH}$ = 4.9 Hz, *H-17*), 2.54 (m, 2H, *H-16*), 1.08 (t, 2H, $^{3}J_{HH}$ = 7.2 Hz, *-NH_2*), -1.14 (s, 3H, Ni-*CH_3*).

^{19}F{^{1}H} NMR (376 MHz, C_6D_6/C_6F_6, 300 K): δ (ppm) = -81.4 (t, J_{FF} = 10 Hz, J_{FF} = 2 Hz, 12F, *CF_3*), -111.0 (t, J_{FF} = 14 Hz, 8F, C_{arom}*CF_2*), -121.1 to -122.2 (m, 32F, *CF_2*), -122.9 (m, 8F, *CF_2*), -126.4 (m, 8F, *CF_2*).

^{13}C{^{1}H} NMR (101 MHz, C_6D_6/C_6F_6, 300 K): δ (ppm) = 167.4 (s, *C-9*), 163.3 (s, *C-11*), 150.5 (s, *C-8*), 149.9 (s, *C-13*), 141.7 (m, *C-15*, *C-4*), 133.4 (s, *C-5*), 132.7 (t, $^{3}J_{CF}$ = 6 Hz, *C-3*), 131.4 (s, *C-6*), 130.7 (t, $^{2}J_{CF}$ = 25 Hz, *C-2*), 127.1 (s, *C-7*), 124.8 (m, *C-1*), 122-105 (*-C_8F_{17}, broad due to multiple $^{X}J_{CF}$ couplings*), 119.9 (s, *C-10*), 96.8 (s, *C-12*), 72.5, 72.2, 71.5-70.7, 70.4 (m, *C*-PEG, *C-14, C-17*), 58.7 (s, *C-18*), 43.3 (s, *C-16*), -13.3 (s, Ni-*CH_3*).

Elemental analysis (%) for $C_{147}H_{196}F_{68}I_2N_2NiO_{45}$: Found (Calculated)
C 41.40 (40.91); H 5.13 (4.58); N 0.80 (0.65)

2.5 Cyclic voltammetry of complexes

All electrochemical measurements were carried out with a computer-controlled BAS potentiostat, connected to a costum-built cyclindrical one-compartment cell (Prof. Winter research group). The experiments were performed in a glovebox under inert atmosphere. A spiral-shaped Pt wire and an Ag wire, sealed into glass capillaries, were used as counter and reference electrodes and introduced at opposite sides of the cell. A platinum electrode was used as working electrode and introduced through the top port of the cell. Prior to all experiments, the working electrode was polished with 1 µm and 0.25 µm diamond paste (purchased from Buehler-Wirtz). The complex was dissolved in approximately 6 mL of dichloromethane mixed with NBu_4^+ PF_6^- (0.1 M) as supporting electrolyte. Referencing was performed by addition of decamethylferrocene ($Cp^*{}_2Fe$) as internal standard to the analyte solution, after all data of interest had been acquired, and another set of scans was recorded. Final referencing was performed against the ferrocene/ferrocenium couple with $E_{1/2}(Cp^*{}_2Fe)$ = -550 mV vs. $Cp_2Fe^{0/+}$. Electrochemical data were acquired with a standard sweep rate of 400 mV s^{-1}. A 10 Hz noise filter was applied to enhance the signal quality. For complex **1-C_8F_{17}/Pyr** the measurement was performed in presence of the reference only, as precipitation of decomposition products (presumably ligand) at the electrode surface hindered multiple high quality measurements.

All cyclic voltammetry measurements of complexes **1/Pyr** and **2/Pyr** showed oxidation and reduction transitions for the Ni(II)/Ni(III) pair. Only partial reversibility was observed, in line with an expected rapid decomposition of the formed Ni(III) species. Additional measurements of the free salicylaldehydes performed under otherwise identical conditions did not show redox transitions in the region of the corresponding pyridine precatalysts, suggesting metal centered oxidation and reduction processes.

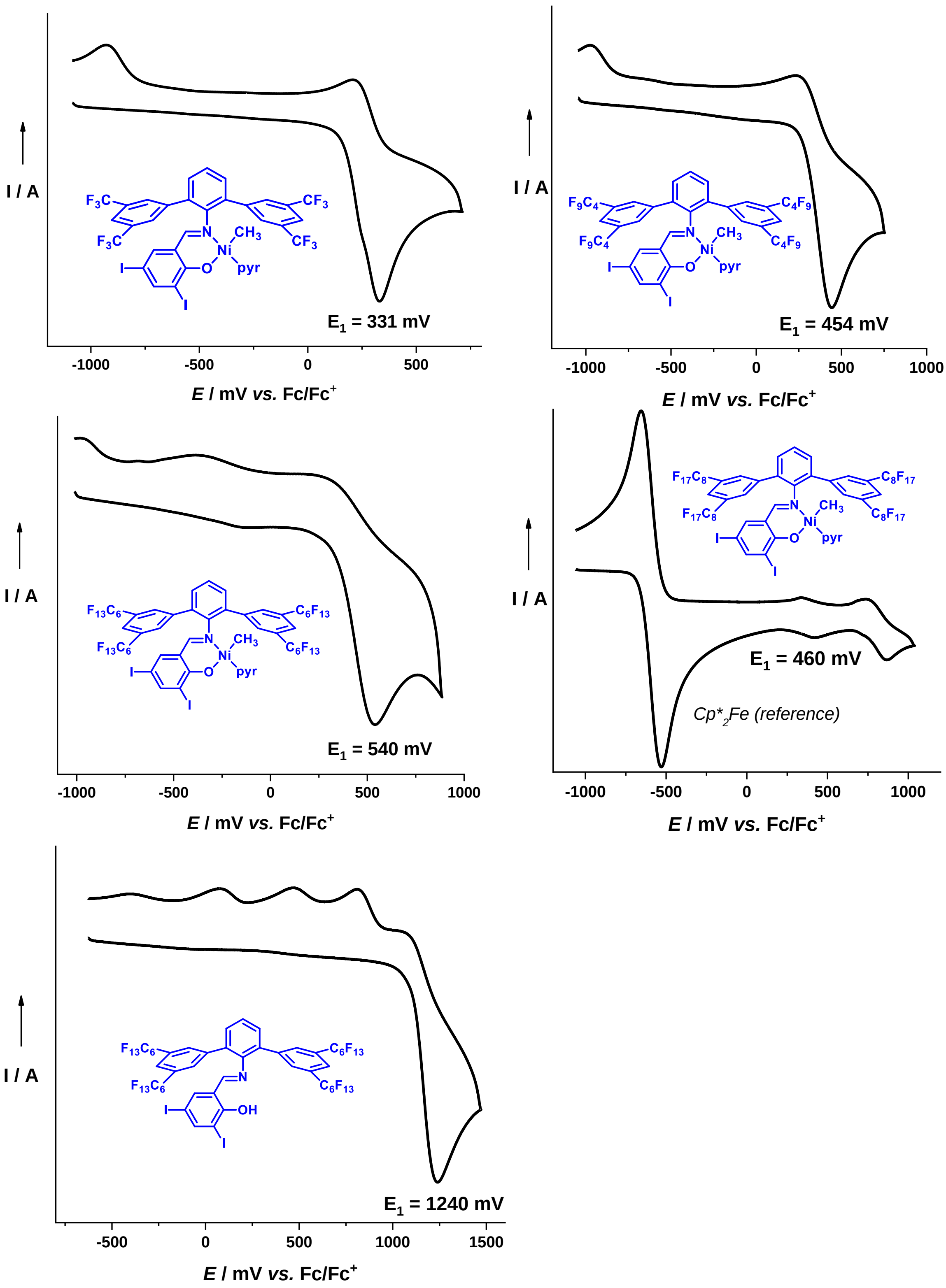

Supplementary Figure 8. Cyclic voltammograms of complexes **1/Pyr**, and salicylaldimine **1d-C_6F_{13}**.

2.6 Polymerization procedures

Polymerization experiments in toluene

All ethylene polymerizations in toluene were conducted in a *Büchi miniclave* reactor with a 280 mL vessel. The reactor was equipped with a mechanical stirrer, a heating and cooling jacket connected to a thermostat, a thermocouple dipping into the polymerization mixture and a nitrogen/vacuum supply. Prior to all polymerization experiments, the reactor was evacuated and heated up (thermostat temperature: 90 °C). When the reactor temperature was > 60 °C, the reactor was flushed with nitrogen and evacuated three times. The reactor was brought 5 °C below the desired reaction temperature. The reactor was then filled with 100 mL of toluene via cannula transfer and the solution stirred with 500 rpm. The catalyst was dissolved in 5 mL of toluene and transferred into the reactor via syringe. Immediately after addition, the stirring rate was increased to 1000 rpm and the reactor pressurized to the desired pressure. During the pressurization procedure the temperature was adjusted to the desired reaction temperature. All experiments were conducted at constant pressure over the entire polymerization experiment with the ethylene feed controlled and monitored by *Bronkhorst* mass flow meters. After the stated reaction time, the ethylene flow was stopped and the reactor carefully vented. The reactor content was poured into 300 mL of methanol and stirred for 30 minutes. The precipitated polymer was filtrated, washed with methanol and dried in a vacuum oven (60 °C, 30 mbar) overnight.

Polymerization experiments aqueous surfactant solution

All ethylene polymerizations in water were conducted in a *Büchi ecoclave* reactor with a 600 mL vessel. The reactor was equipped with a heating and cooling jacket connected to a thermostat, a mechanical stirrer, a nitrogen/vacuum supply, and an ultrasonotrode (*Hielscher UIP250*) and a thermocouple couple both dipping into the reaction mixture. A *Bronkhorst* MassFlow apparatus consisting of two flow meters (up to 20 g L^{-1} and 200 g L^{-1} ethylene), a pressure meter and a compressed air-driven badger valve was used to work under constant ethylene pressure. All gas valves and devices were connected to a HiTec Zang LabBox and operated by HiTec Zang LabVision® software (ver. 2.13). The software allowed for a precise process visualization, control and recording of all relevant parameters in one single process flow chart. The integration of HiText™ programming language enabled the construction of several automation scripts (e.g. for pressurization, temperature control, venting, etc.). Prior to all polymerization experiments, the reactor was evacuated and heated up (thermostat temperature: 90 °C) using a custom HiText™ program. When the reactor temperature was > 60 °C, the reactor was flushed with nitrogen, evacuated three times and automatically cooled down to 13 °C. The desired amount of surfactant (e.g. sodium dodecyl sulfate) and base (e.g.

cesium hydroxide) and a magnetic stirrer bar were put into a Schlenk flask, and the flask was transferred into a glovebox. After addition of lipophilic solvent (e.g. mesitylene) and catalyst, the flask was sealed, brought outside the glovebox and water was added under vigorous stirring via cannula transfer. The clear orange solution (after 3-5 minutes of stirring) was then transferred to the reactor and stirred at 500 rpm. The polymerization experiment was started using a costum HiText™ program with a graphical interface to adjust ultrasound application power and duration, reaction time and reaction temperature control. The reaction mixture was then automatically treated with ultrasound (usually for 4 minutes with 120 watt power) and the solution temperature monitored to stay around 15 °C. Immediately afterwards, the stirring rate was increased to 1000 rpm and the reactor pressurized stepwise to 40 bar ethylene pressure within 30 seconds. The ethylene flow was then controlled and recorded by the mass flow meter to ensure a constant pressure over the entire polymerization experiment. The reaction temperature was automatically adjusted to 15 °C. After the desired reaction time, the pressure was automatically released stepwise. Below 15 bar residual pressure, the reactor was vented manually into a beaker to collect migrating dispersion due to foaming. The entire dispersion was weighed, filtered over cotton wool and the solid content determined by precipitation of a 50 g aliquot of dispersion in 300 mL of methanol. After stirring for 30 minutes, the precipitated bulk polymer was filtered and washed thoroughly with water and methanol, and dried in a vacuum oven (60 °C, 30 mbar) overnight.

2.7 Process design for polymerization in aqueous media

Influence of the fluorocarbon chain length in the catalyst structure

The length of the perfluorinated alkyl chain in the catalyst structure was found to have a strong impact on the catalytic properties of the active nickel center, as studied in detail in toluene as a reaction medium with lipophilic catalyst precursors (pyridine as labile ligand; cf. Figure 2). These trends were also confirmed for hydrophilic analogues in aqueous system (Supplementary Table 1).

Supplementary Table 1. Results of aqueous polymerization experiments performed with catalysts bearing different fluorocarbon chain lengths.

entry	catalyst	yield PE[a] [g]	M_n[b] [10^3 g mol^{-1}]	M_w / M_n[b]	chains/ [Ni]	T_m[c] [°C] (Crystallinity [%])	Particle size[d] [nm]
**1**	2/PEG	0.28	206	1.5	0.2	137 (69) / 133 (54)	16 (0.36)
**2**	1-C_4F_9/PEG	2.18	324	1.2	0.9	141 (79) / 136 (58)	18 (0.30)
**3**	1-C_6F_{13}/PEG	4.53	626	1.2	1.0	139 (79) / 135 (55)	23 (0.17)
**4**	1-C_8F_{17}/PEG	3.76	503	1.1	1.0	141 (73) / 136 (52)	24 (0.48)

conditions: 40 bar ethylene pressure, 15 °C reaction temperature, 6.0 g sodium dodecyl sulfate, 1.5 g cesium hydroxide, 0.1 mL hexafluorobenzene, 7.5 µmol catalyst loading, 30 minutes reaction time, in 150 mL water. a: determined via precipitation from methanol; b: determined via GPC at 160 °C in 1,2,4-trichlorobenzene; c: determined by DSC (10 K min^{-1} heating rate), reported as [first heating / second heating] d: volume average size from DLS (particle size polydispersity index)

Also in aqueous systems, yields and molecular weights generally increase with increasing length of the fluorocarbon group in the catalyst structure (cf. Supplementary Figure 9).

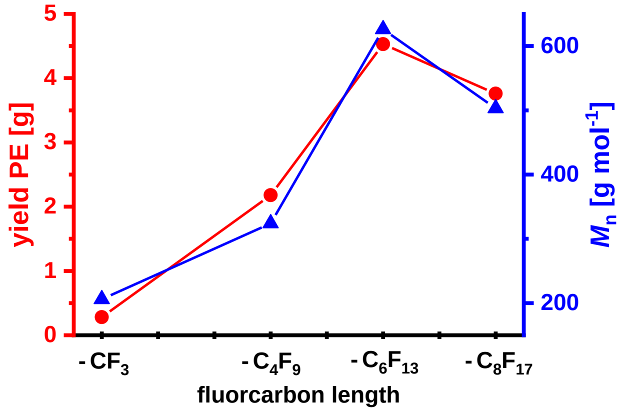

Supplementary Figure 9. Polymer yield and molecular weight in dependence of the fluorocarbon chain length in the catalyst for aqueous system.

This shows that these trends are independent of the solvent nature, and are based on a direct electronic influence caused by the fluorocarbon groups on the active nickel center. The experiments further underline catalyst **1-C_6F_{13}/PEG** to be the catalyst of choice.

An influence of the hydrophobic perfluoroalkyl on the dissociation rate of the labile amino-PEG ligand in aqueous system was also found. This is evident from low chains-per-nickel ratios for

catalyst **2/PEG** (entry 1, Supplementary Table 1) as the labile ligand seems to bind strongly to the metal center and hinders a sufficient activation. This was not observed for catalysts **1/PEG** with their hydrophobic perfluoroalkyl groups that force the highly hydrophilic labile ligand in close proximity to dissociate as revealed by chains-per-nickel rations close to unity (entry 2-4, Supplementary Table 1). The catalyst was further modified by changing the length of the PEG-unit of the labile coordinated ligand molecule (1981 g mol^{-1} vs. 5516 g mol^{-1}). Catalyst bearing longer PEG-units generally showed a slightly higher activity, presumably due to an increased water-solubility resulting in an enhanced dissociation rate (entries 1-3 vs. entries 4-6, Supplementary Table 4). Other than this, an essentially similar behavior was observed.

Choice of lipophilic solvents in aqueous systems

To ensure a maximum degree of dispersion of the catalyst, followed by an undisturbed particle growth process, the addition of small amounts organic solvents to the aqueous surfactant solution was studied. A range of aliphatic and aromatic, as well as fluorinated and non-fluorinated compounds were investigated to further reveal the nature of this effect (cf. Supplementary Figure 10). These different organic compounds were studied in aqueous polymerization experiments with catalyst **1-C_6F_{13}/PEG** under otherwise identical conditions (results summarized in Supplementary Table 2). All solutions were sonicated with ultrasound prior to ethylene pressurization to ensure a sufficient mixing. In all cases, a crucial influence of the additive was found, when compared to the control experiment without added solvent (entries 26 and 27).

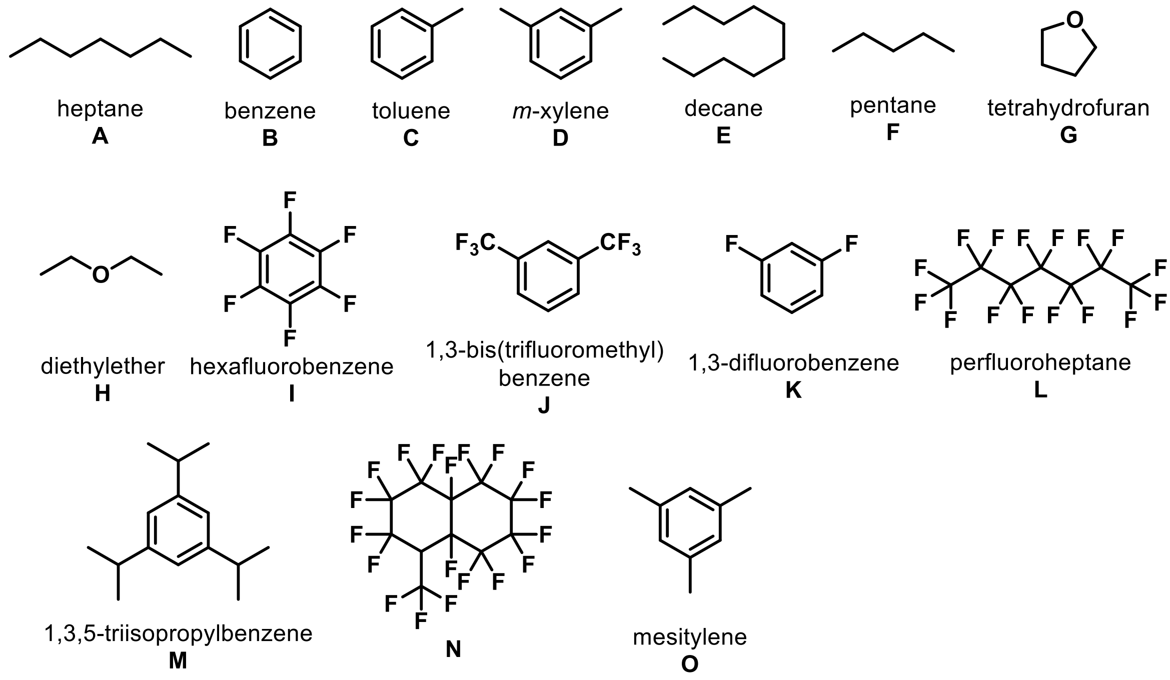

Supplementary Figure 10. Organic compounds studied as lipophilic solvents for polymerizations in aqueous surfactant solution. The labels refer to the "additive" column in supplementary table 2.

Supplementary Table 2. Results of polymerization experiments in aqueous surfactant solution using different solvents as additives. For designation of additives cf. Supplementary Figure 10.

entry	additive	reaction time [min]	yield PE[a] [g]	M_n[b] [10^3 g mol^{-1}]	M_w / M_n[b]	chains/ [Ni]	T_m[c] [°C] (Crystallinity [%])	Particle size[d] [nm]
1	**A**	240	9.11	1229	1.3	1.0	145 (74) / 137 (58)	22 (0.22)
2		120	7.21	842	1.4	1.1	143 (68) / 137 (50)	21 (0.15)
3		60	6.25	738	1.1	1.2	142 (65) / 136 (49)	19 (0.11)
4		30	3.84	385	1.1	1.3	141 (65) / 137 (48)	16 (0.11)
5	**B**	30	3.90	376	1.2	1.4	139 (69) / 136 (49)	16 (0.14)
6	**C**	30	4.29	623	1.2	0.9	140 (64) / 135 (47)	15 (0.16)
7	**D**	240	9.22	1242	1.1	1.0	143 (66) / 135 (47)	21 (0.08)
8		60	7.24	778	1.4	1.2	142 (65) / 137 (41)	19 (0.06)
9		30	4.88	779	1.1	0.8	139 (65) / 135 (45)	16 (0.23)
10	**E**	240	6.73	882	1.3	1.0	143 (67) / 136 (48)	28 (0.37)
11		60	6.29	893	1.2	0.9	142 (66) / 136 (48)	19 (0.13)
12	**F**	60	4.52	870	1.3	0.7	142 (65) / 136 (48)	19 (0.13)
13	**G**	30	3.65	466	1.2	1.0	142 (62) / 135 (44)	17 (0.21)
14	**H**	60	2.60	536	1.1	0.7	141 (68) / 136 (44)	19 (0.12)
15	**I**	240	8.32	912	1.2	1.2	144 (67) / 136 (47)	21 (0.13)
16		120	6.20	802	1.3	1.0	142 (72) / 136 (52)	22 (0.10)
17[e]		60	4.56	493	1.1	1.2	143 (68) / 136 (49)	16 (0.17)
18		30	4.00	742	1.2	0.7	140 (65) / 136 (44)	16 (0.15)
19	**J**	240	5.20	669	1.3	1.0	144 (66) / 137 (49)	21 (0.11)
20	**K**	240	8.53	1113	1.2	1.0	142 (65) / 135 (46)	20 (0.13)
21	**L**	30	2.95	483	1.3	0.8	141 (65) / 136 (50)	18 (0.26)
22	**M**	240	3.62	506	1.2	1.0	143 (62) / 137 (46)	20 (0.37)
23	**N**	30	3.55	386	1.1	1.2	143 (68) / 137 (49)	18 (0.40)
24	**O**	240	9.91	1348	1.2	1.0	142 (66) / 135 (45)	24 (0.04)
25		60	7.07	1020	1.1	0.9	140 (65) / 134 (46)	19 (0.05)
26	-	240	4.80	701	1.5	0.9	144 (66) / 138 (48)	348 (0.50)
27		30	3.42	370	1.1	1.2	141 (68) / 137 (51)	17 (0.30)

conditions: 7.5 µmol precatalyst **1-C_6F_{13}/PEG**, 6.0 g SDS, 1.5 g CsOH, 0.3 mL additive, 40 bar ethylene pressure, in 150 mL water, 15 °C reaction temperature, (for experiments with 60, 120, 240 and 360 minutes reaction time); 7.5 µmol precatalyst, 4.5 g SDS, 1.5 g CsOH, 0.2 mL additive, 40 bar ethylene pressure, 15 °C (for experiments with 30 minutes reaction time); catalyst solution was ultrasonicated (4 min, 120 Watt) prior to ethylene pressurization; a: determined via precipitation from methanol; b: determined via GPC at 160 °C in 1,2,4-trichlorobenzene; c: determined by DSC (10 K min^{-1} heating rate), reported as [first heating / second heating] d: volume average size from DLS (particle size polydispersity index); e: 0.1 mL additive added

The addition of small amounts organic lipophilic compounds (0.1 vol-%) significantly increased the polymer yield and the quality of the dispersion obtained after polymerization in terms of stability and particle size distribution, quantified by DLS measurements, in all cases (cf. Supplementary Figure 11).

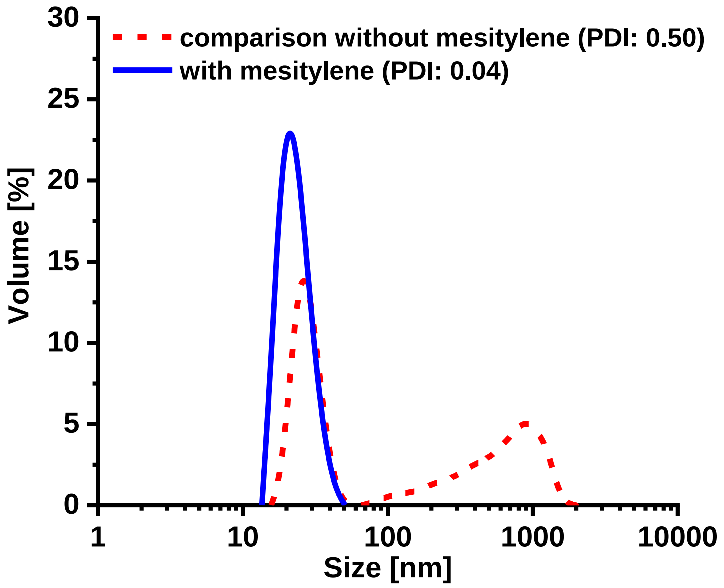

Supplementary Figure 11. DLS traces of PE dispersions obtained from polymerization experiments in water with mesitylene added (blue curve; Table 2, entry 4) and without (red curve) under otherwise identical conditions.

However, the choice of compound does impact the outcome of polymerization (e.g. entry 19 vs. entry 24). The overall data gives no indication that the lipophilic compound directly interacts with the active center and alters its catalytic properties with regard to chain growth and chain microstructure. This is evident from the fact that in all cases polyethylene with comparable thermal properties was formed in a living polymerization as suggested from chains per nickel ratios close to unity and narrow molecular weight distributions (molecular weights differ in accordance with yield). Rather the polymerization results appear to depend on the lipophilic solvents ability to dissolve/distribute the precatalyst/active center in solution, respectively, and on its ability to form stable and highly-dispersed emulsions in the water/SDS system. With this in mind, the organic compound does not influence the polymer formation mechanism itself, but rather the way the catalysts' polymer chain forms ordered single nanocrystals during chain growth and in particular the initial stages of the polymerization experiment. As alkanes and alkylbenzenes are known to form highly stable emulsions with SDS[6], their superior role as lipophilic solvents compared to e.g. fluorinated solvents is plausible. The importance of a sufficient emulsion formation is further underlined by direct correlation of particle size distribution and yield. Reduced yields are usually found for dispersions with broad particle size distributions and vice versa. As particle/catalyst agglomeration disturbs chain growth and consequently influences yield, respectively, molecular weight, the found relation is expected.

Supplementary Table 3. Polymerization experiments in aqueous surfactant solution with different amounts of mesitylene as a lipophilic solvent.

entry	mesitylene [mL]	yield PE[a]	M_n[b] [10^3 g mol^{-1}]	M_w / M_n[b]	chains/ [Ni]	T_m[c] [°C] (Crystallinity [%])	Particle size[d] [nm]
**1**	0.1	4.65 g	772	1.3	0.8	145 (67) / 139 (45)	20 (0.09)
**2**	0.15	5.22 g	666	1.3	1.0	144 (63) / 136 (41)	20 (0.05)
**3**	0.3	7.07 g	1020	1.1	0.9	140 (65) / 134 (46)	19 (0.05)
**4**	0.6	7.27 g	1141	1.2	0.9	141 (68) / 135 (45)	20 (0.04)
**5**	0.75	7.09 g	846	1.2	1.1	143 (64) / 135 (43)	20 (0.03)
**6**	0.9	7.57 g	941	1.3	1.1	139 (64) / 133 (44)	20 (0.03)

conditions: 7.5 µmol precatalyst **1-C_6F_{13}/PEG**, 6.0 g SDS, 1.5 g CsOH, 40 bar ethylene pressure, 15 °C, 60 minutes reaction time, in 150 mL water; catalyst solution was ultrasonicated (4 min, 120 Watt) prior to ethylene pressurization; a: determined via precipitation from methanol; b: determined via GPC at 160 °C in 1,2,4-trichlorobenzene; c: determined by DSC (10 K min^{-1} heating rate), reported as [first heating / second heating] d: volume average size from DLS (particle size polydispersity index)

We found mesitylene to be the solvent of choice in that it promotes high yields and molecular weights as well as narrow particle size distributions (entries 26 and 27, Supplementary Table 2). For the case of mesitylene even in the absence of ultrasonication similar favorable polymerization results were found, which underlines the suitability of this system to distribute the catalyst in the initial reaction mixture well. Studies with variable amounts of mesitylene showed the minimal concentration required to be 0.2 vol-% (entry 2, Supplementary Table 3). Experiments performed with lower portions showed reduced yields and broadening in particle size distributions (entries 1-2, Supplementary Table 3). Above this concentration no significant impact of mesitylene loadings were found.

Surfactant

The surfactant is of major importance for a favorable particle formation process. Experiments conducted without surfactant lead to immediate polymer precipitation and polymerization activity ceasing within minutes. As the surfactant is absorbed during the reaction on the growing crystals surface, it is one major limiting factor of that reaction type and limits the polymerization progress. We further hypothesize that the employed hydrophobic catalyst systems with their perfluorinated alkyl chains require a certain amount of free surfactant in solution to be stabilized in the initial reaction mixture in order to perform undisturbed chain and particle growth. Usually polyethylene of 1.5- to 2.0-times the sodium dodecyl sulfate content (by mass) was found to be formed before the activity suddenly decreased. This is evident from the ethylene mass flow curves recorded during the polymerization experiment that allows for a precise monitoring of the catalytic activity (cf. Supplementary Figure 12).

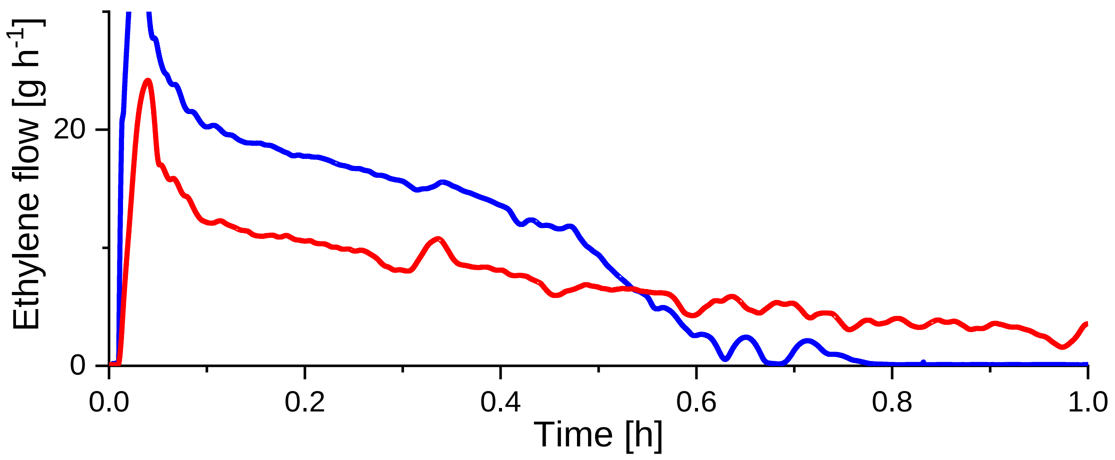

Supplementary Figure 12. Ethylene mass flow traces of aqueous polymerization experiments with different catalyst loadings under otherwise identical conditions. With high catalyst loading (7.5 µmol, blue curve), a sudden decrease in activity within 1 hour reaction time due to insufficient surfactant concentration is visible. With lower catalyst loading (5 µmol, red curve), the initial overall ethylene consumption is lower but lasts for at least 1 hour reaction time as enough free surfactant per active center is available (Cond.: 7.5 or 5 µmol precatalyst **1-C_6F_{13}/PEG**, 4.5 g SDS, 1 g CsOH, 0.5 mL mesitylene, catalyst solution ultrasonicated prior to ethylene pressurization (2 min, 120 watt), 15 °C reaction temperature, 40 bar ethylene pressure, 1 hour reaction time).

Considering this, a sufficiently high amount of surfactant per nickel center is desirable to provide a sufficient colloidal stabilization of both the catalyst in the (initial) reaction mixture, as well as the formed nanoparticles. However, the surfactant concentration is limited by its solubility in water at 15 °C, the increasing viscosity of the reaction mixture that hinders sufficient mixing and the potential formation of ethylene hydrate[7]. To increase the amount of free surfactant per active center, the total volume of the reaction mixture was varied while keeping the surfactant (and other additives) concentration constant and reducing the catalysts concentration (identical amounts of catalyst were used in increased reaction mixture volumes). Results are summarized in Supplementary Table 4. The yields were found to increase for experiments, where more free surfactant per nickel center is available. On the other hand, also molecular weights and particle size were found to be higher. This shows that the surfactant content directly influences the amount of polymer that is formed by an active center. A high content enables the synthesis of particles with > 80 nm lateral size and molecular weights of $M_n = 3 \times 10^6$ g mol^{-1}, while yield, molecular weight and particle size are reduced when half the amount of surfactant is used (entries 4 and 7, Table 2). In contrast, the molecular mechanism of chain growth itself is not influenced as in all cases polyethylene with comparable thermal properties is formed in a living polymerization.

Supplementary Table 4. Polymerization experiments in aqueous surfactant solution with different surfactant per active center ratios, respectively, different precatalyst concentrations. Two sets of identical experiments were performed with precatalysts bearing labile ligands with different PEG-chain lengths.

entry (cond.)	surfactant / active center [10^3 molecules]	yield PE[a]	M_n[b] [10^3 g mol^{-1}]	M_w / M_n[b]	chains/ [Ni]	T_m[c] [°C] (Crystallinity [%])	Particle size[d] [nm]
**1 (A)**	4.2	8.27	994	1.2	1.1	142 (68) / 134 (46)	23 (0.06)
**2 (B)**	5.5	12.98	1686	1.3	1.0	143 (64) / 133 (42)	30 (0.04)
**3 (C)**	8.3	17.08	1974	1.2	1.2	142 (65) / 135 (38)	34 (0.03)
**4 (A)**	4.2	10.47	1214	1.3	1.1	142 (68) / 134 (46)	31 (0.07)
**5 (B)**	5.5	15.64	1810	1.3	1.1	141 (65) / 133 (43)	37 (0.05)
**6 (C)**	8.3	18.42	2616	1.3	0.9	142 (68) / 134 (46)	43 (0.06)

conditions: 40 bar ethylene pressure, 2 hours reaction time, 15 °C reaction temperature, 7.5 µmol catalyst loading (**1-C_6F_{13}/PEG**, entries 1-3 with M(H_2N-PEG-OMe) = 1981 g mol^{-1}, entries 4-7 with M(H_2N-PEG-OMe) = 5516 g mol^{-1}), 0.137 mol L^{-1} sodium dodecyl sulfate, 0.067 mol L^{-1} cesium hydroxide, 5 mL L^{-1} mesitylene, catalyst solution was ultrasonicated (4 min, 120 Watt) prior to ethylene pressurization; in 150 mL (A), 300 mL (B) or 450 mL (C) water. a: determined via precipitation from methanol; b: determined via GPC at 160 °C in 1,2,4-trichlorobenzene; c: determined by DSC (10 K min^{-1} heating rate), reported as [first heating / second heating] d: volume average size from DLS (particle size polydispersity index)

2.8 Particle size statistics of PE nanocrystal dispersions

For particle size and volume statistics, lateral dimensions and the corresponding basal plane surface area were accessed by TEM imaging and particle thickness (*H*) was determined by AFM. Only isolated particles clearly oriented flat on the grid surface were considered for TEM analysis. The particles of different samples have a hexagonal, truncated-lozenge or lozenge shape, respectively, depending on the progress of morphology evolution as given by the duration of the polymerization experiment. The limited electron contrast of organic polymers results in a limited resolution of the edges, particularly relevant for particles with (lateral) sizes of ca. < 30 nm. To circumvent subjective errors of a manual analysis of particle dimensions and surface areas, an ellipsoidal fit of the particles employing analysis of TEM images via ImageJ/Fiji[8-10] software was used. This fit was verified to yield accurate areas even for lozenge shaped particles (vide infra). The TEM analysis routine provided values for the full-axis lengths *A* and *B* over all analyzed particles. The equivalent diameter *D* (diameter of a circle with same surface area as the fitted ellipse) was calculated according to Supplementary Equation 1 and used as characteristic parameter for comparison of lateral particle sizes of different samples.

$$D = 2\sqrt{0.25\,A\,B} \quad (1)$$

The average volume of a particle $V_{\mathbf{part}}$ was calculated according to Supplementary Equation 2, employing the average thickness (*H*) from AFM (vide infra).

$$V_{\mathrm{part}} = \frac{1}{6}\pi\,A\,B\,H \quad (2)$$

The average mass of one particle $m_{\mathbf{part}}$ was determined by multiplication of $V_{\mathbf{part}}$ with the density ρ_{PE} (Supplementary Equation 3). The density was assumed to be ρ_{PE} = 0.94 g cm^{-1} [11] for these type of nanocrystals (bulk density of UHMWPE). The mass of one polymer chain $m_{\mathbf{chain}}$ was calculated according to Supplementary Equation 4 using the mass-average molecular weight M_W. The chains per particle ratio $N_{\mathbf{chain}}/N_{\mathbf{part}}$ can then be determined by Supplementary Equation 5. The resulting data is summarized in Supplementary Tables 5 and 6.

$$m_{\mathrm{part}} = V_{\mathrm{part}} \cdot \rho_{\mathrm{PE}} \quad (3)$$

$$m_{\mathrm{chain}} = \frac{M_{\mathrm{W}}}{N_A} \ \text{with} \ N_A = 6.022 \cdot 10^{23}\,mol^{-1} \quad (4)$$

$$\frac{N_{\mathrm{chain}}}{N_{\mathrm{part}}} = \frac{m_{\mathrm{part}}}{m_{\mathrm{chain}}} = \frac{V_{\mathrm{part}}\ \rho_{\mathrm{PE}}\ N_A}{M_{\mathrm{W}}} \quad (5)$$

Supplementary Table 5. Statistical particle size data determined via ellipse fit method of TEM pictures. Values *A* and *B* are full-axis lengths of calculated ellipses (standard deviation reported). D is the average lateral particle size, calculated as mean value of *A* and *B* per particle (standard deviation reported).

entry in Table 2	number of particles	*A* [nm]	*B* [nm]	*D* [nm]
1	213	26.0 ± 2.3	22.0 ± 1.7	23.6 ± 1.6
2	238	33.1 ± 3.1	25.3 ± 2.3	28.9 ± 2.2
3	147	38.5 ± 3.6	29.3 ± 2.1	33.6 ± 2.3
4	255	41.9 ± 3.9	32.4 ± 2.7	36.8 ± 2.8
5	325	54.2 ± 7.7	39.2 ± 5.3	46.0 ± 6.1
6	140	63.6 ± 11.4	45.7 ± 7.5	53.9 ± 9.1
7	158	77.0 ± 15.3	53.9 ± 10.2	64.4 ± 12.3

Supplementary Table 6. Values used to calculate the respective chain per particle ratio for data entries in Table 2. The particle height *H* was determined via AFM measurements (vide infra). Particle volume V_{part}, molecular weight M_w and the resulting chains per particle ratios are reported with estimated measured errors/error propagation analysis (vide infra).

entry in Table 2	*H* [nm]	V_{part} [10^{-24} nm]	M_w [10^3 g mol^{-1}]	chains/ particles
1	5.2 ± 0.6[a]	1.6 ± 0.3	760 ± 76	1.2 ± 0.3
2	5.2 ± 0.6	2.3 ± 0.5	1087 ± 109	1.2 ± 0.3
3	5.3 ± 0.6 [a]	3.1 ± 0.6	1402 ± 140	1.3 ± 0.3
4	5.3 ± 0.6	3.8 ± 0.5	1735 ± 174	1.2 ± 0.3
5	5.3 ± 0.6 [a]	5.9 ± 1.2	2371 ± 237	1.4 ± 0.3
6	5.6 ± 0.7 [a]	8.5 ± 1.7	3202 ± 320	1.5 ± 0.4
7	5.6 ± 0.7	12.2 ± 2.4	3952 ± 395	1.7 ± 0.4

[a]Data of measured samples in similar particle size regime was used as the particles thickness do not change significantly during the particle growth process.

The validity of the automated ellipse fit was verified by comparison to manual analysis. This was performed for the case of lozenge particles, as the deviation from an ellipse is the largest for this particle shape (compared to hexagons and truncated lozenges), and as these are the largest type of particles such that the limited resolution of the particle edges weighs in least. Lozenge particles were analyzed by measuring all four side lengths and the short diagonal (cf. Supplementary Figure 13). An exemplary TEM image and the identical pictures with either the marked triangles or the automatically fitted ellipses are shown in Supplementary Figure 14.

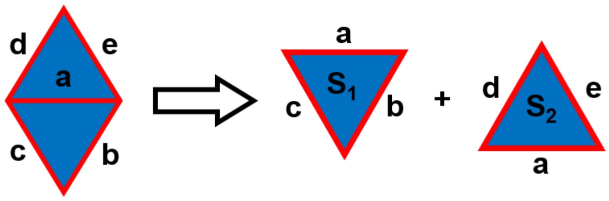

Supplementary Figure 13. Schematic segmentation of a lozenge-shaped particle into two triangles with surface areas S_1 and S_2.

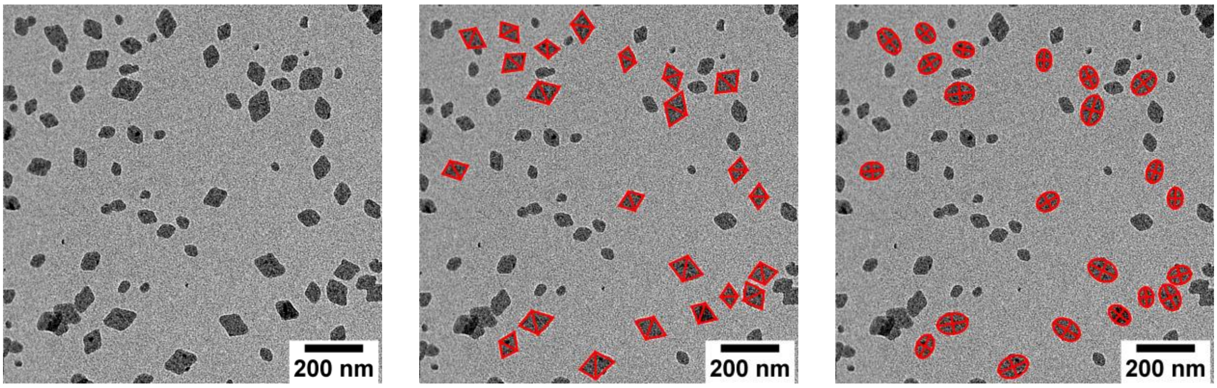

Supplementary Figure 14. TEM image of sample with mixed-size lozenge particles. Left: without analysis; middle: with measured triangle side lengths for surface area determination; right: with fitted ellipses for surface area determination.

The triangles' surface areas S_1 and S_2 is given by Supplementary Equations 6 and 7 and the overall lozenge surface area S_Δ is their sum, Supplementary Equation 8 (standard deviation over 50 particles reported).

$$S_1 = \sqrt{x(x-a)(x-b)(x-c)} \quad with\, x = \frac{a+b+c}{2} \quad (6)$$

$$S_2 = \sqrt{x(x-a)(x-d)(x-e)} \quad with\, x = \frac{a+d+e}{2} \quad (7)$$

$$S_\Delta = S_1 + S_2 = 3932 \pm 928\,\mathrm{nm}^2 \quad (8)$$

With the fitted full-axis lengths A and B determined by the automated ellipse fit, the surface area S_O is given by Supplementary Equation 9 (with standard deviation over 50 particles reported).

$$S_O = 0.25\,\pi\, A\, B = 4373 \pm 1027\,\mathrm{nm}^2 \; (9)$$

Both methods gave similar results for an identical set of particles with only 10 % difference. This shows that the ellipse-fit routine is not only suited for surface determination of hexagonal/round particles but can also describe lozenge-shaped exemplars with satisfactory accuracy. In addition, the statistical distribution is identically expressed in both cases (Supplementary Figure 15).

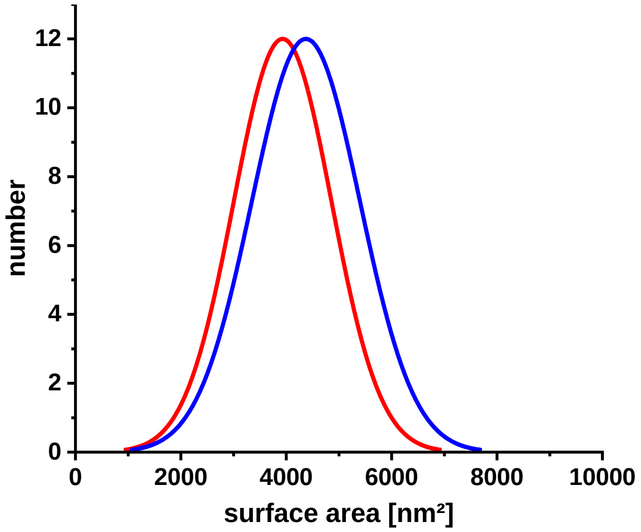

Supplementary Figure 15. Particle surface area distribution of same set of particles. Either automatically determined by an ellipse-fit routine (blue curve) or manually measured with the triangle dissection (red curve).

Considering the propagation of systematic errors, we estimated the measurement error of the molecular weight determined by GPC ΔM_W to be ± 10 %, the error of the particle volume determined by tem statistics ΔV_part to be ± 20 % and the particle density error $\Delta\rho_\mathrm{PE}$ to be ± 10 %. Application of a Gaussian error propagation approach to Supplementary Equation 5 yields the error in determination of the chain per particle ratio by Supplementary Equation 10. Results for V_part, M_w and the chain-per-particle ratios are listed in Supplementary Table 6.

$$\Delta N_\mathrm{C/P} = \sqrt{\left(\frac{\partial N_\mathrm{C/P}}{\partial M_\mathrm{W}}\right)^2 \Delta {M_\mathrm{W}}^2 + \left(\frac{\partial N_\mathrm{C/P}}{\partial V_\mathrm{part}}\right)^2 \Delta {V_\mathrm{part}}^2 + \left(\frac{\partial N_\mathrm{C/P}}{\partial \rho_\mathrm{PE}}\right)^2 \Delta {\rho_\mathrm{PE}}^2}$$

$$= \sqrt{\left(-\frac{V_\mathrm{part}\ \rho_\mathrm{PE}\ N_\mathrm{A}}{{M_\mathrm{W}}^2}\right)^2 \Delta {M_\mathrm{W}}^2 + \left(\frac{\rho_\mathrm{PE}\ N_\mathrm{A}}{M_\mathrm{W}}\right)^2 \Delta {V_\mathrm{part}}^2 + \left(\frac{V_\mathrm{part}\ N_\mathrm{A}}{M_\mathrm{W}}\right)^2 \Delta {\rho_\mathrm{PE}}^2} \quad (10)$$

$$with\ \Delta M_\mathrm{W} = 0.1\ M_W\ , \Delta V_\mathrm{part} = 0.2\ V_{part}\ , \Delta\rho_\mathrm{PE} = 0.1\ \rho_\mathrm{PE}$$

2.9 AFM measurements

Atomic force microscopy (AFM) measurements to determine the particles height were performed on samples generated by spincoating of dialyzed dispersions on Si wavers using intermittent contact mode with Si cantilevers.

Supplementary Table 7. Values for particle heights H determined via AFM measurements.

entry in Table 2	number of particles	*H* [nm]
2	31	5.2 ± 0.6
4	29	5.3 ± 0.6
7	36	5.6 ± 0.7

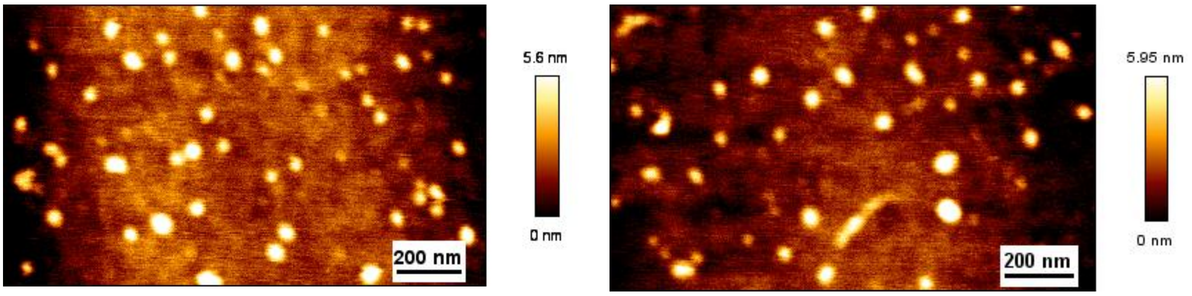

Supplementary Figure 16. AFM image sections of dispersion sample obtained in aqueous polymerization (Table 2, entry 3).

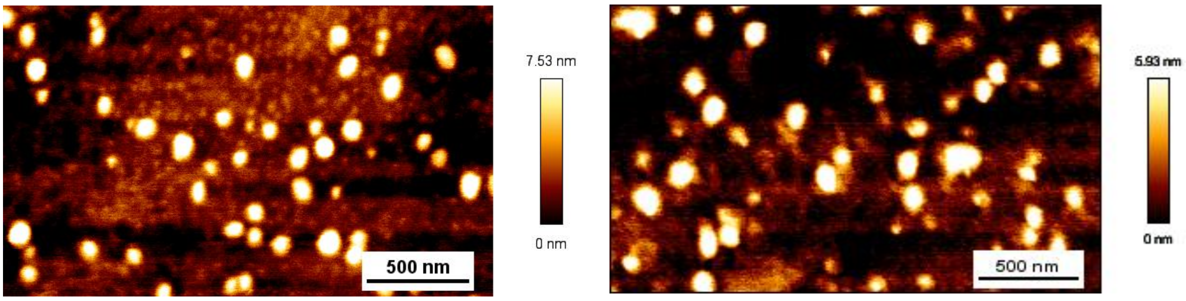

Supplementary Figure 17. AFM image sections of dispersion sample obtained in aqueous polymerization (Table 2, entry 7).

3. Supplementary Figures

3.1 Histograms from particle size TEM statistics

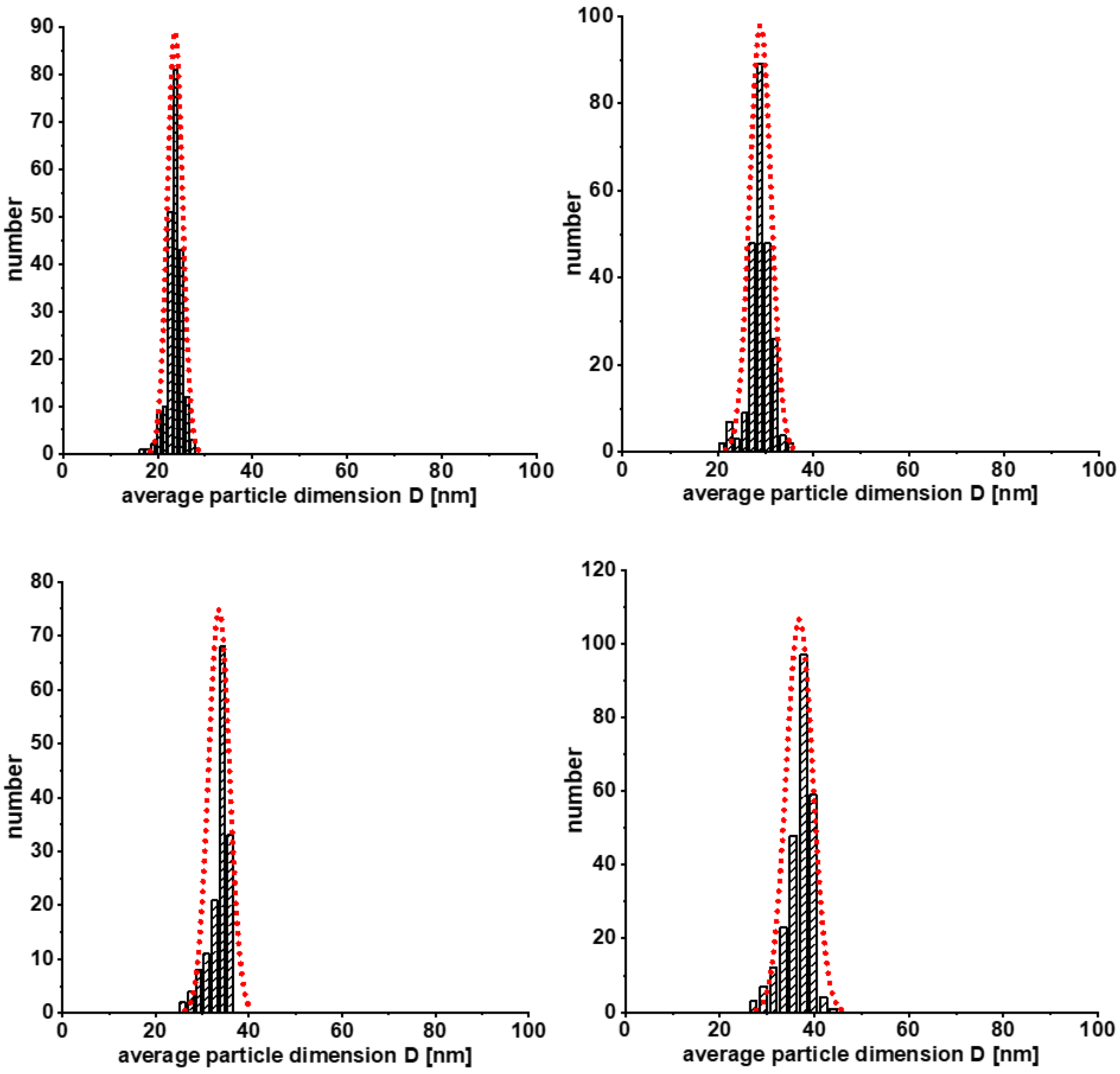

Supplementary Figure 18. Histograms of TEM statistical data. The class size was chosen depending on the determined statistical standard deviation exclusively for every data set. A distribution curve (red) is shown assuming Gaussian particle size distribution. Data from entries in Table 2 (top, left: entry 1 | top, right: entry 2 | bottom, left: entry 3 | bottom, right: entry 4).

3.2 TEM images of PE nanocrystals

Exemplary TEM sections used for statistical calculations

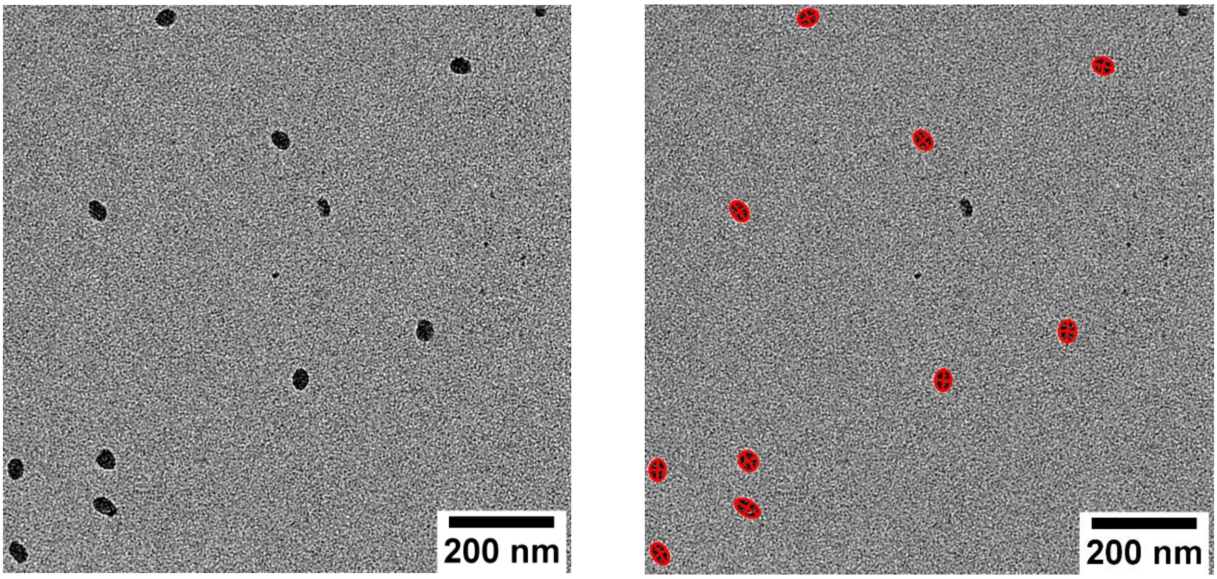

Supplementary Figure 19. TEM image of polyethylene dispersion (entry 3, Table 2); applied ellipse fit shown (right).

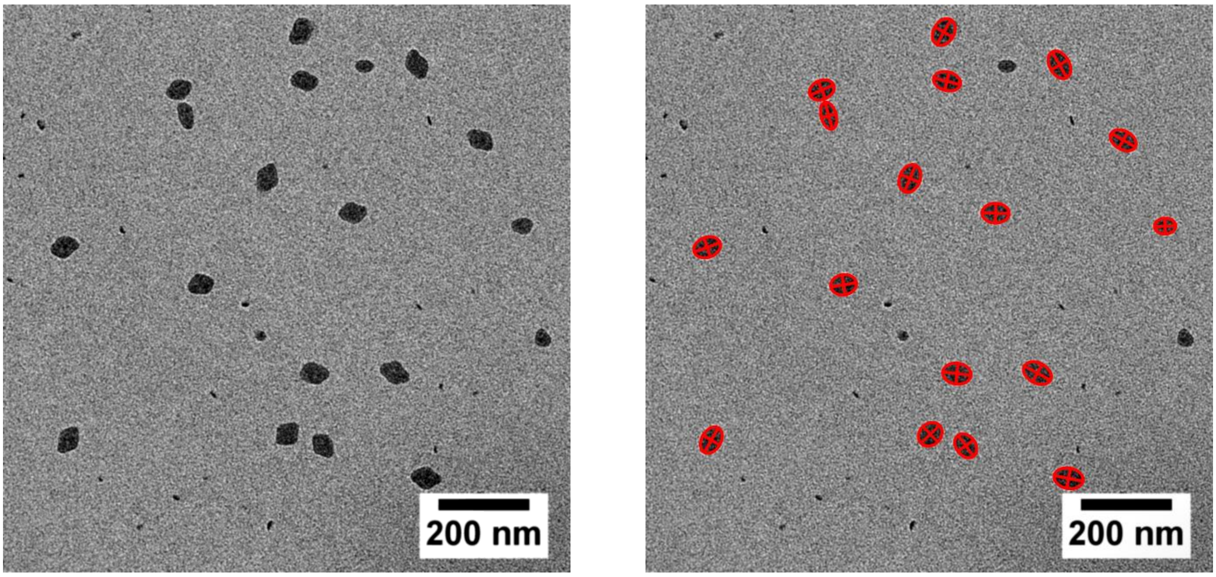

Supplementary Figure 20. TEM image of polyethylene dispersion (entry 6, Table 2); applied ellipse fit shown (right).

TEM overview images of assembled particles

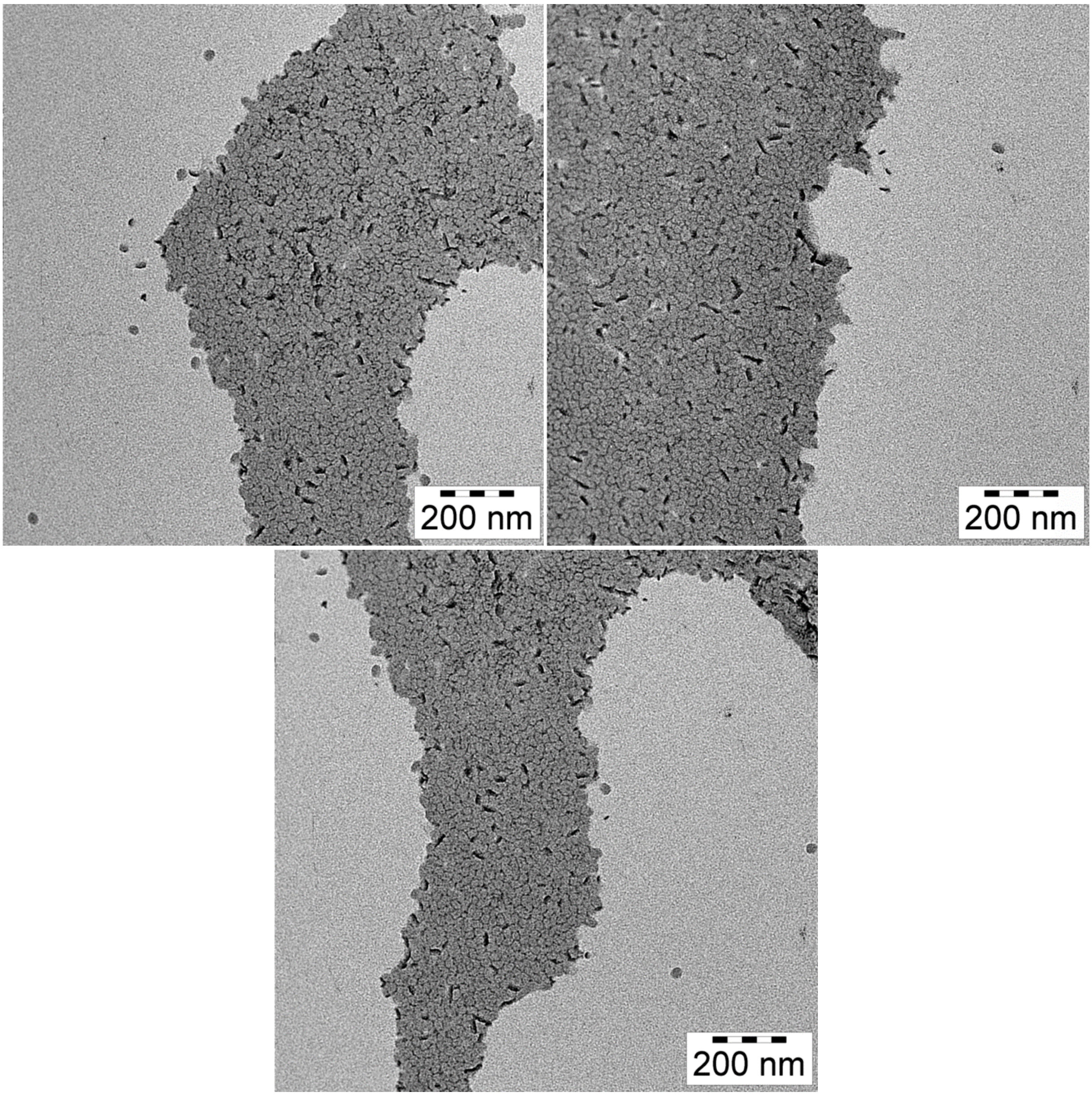

Supplementary Figure 21. Assemblies of nanoparticles obtained in aqueous polymerization (entry 2, Table 2) to layers by drop casting of dialyzed dispersion. Dark structures are particles standing perpendicular to the electron beam.

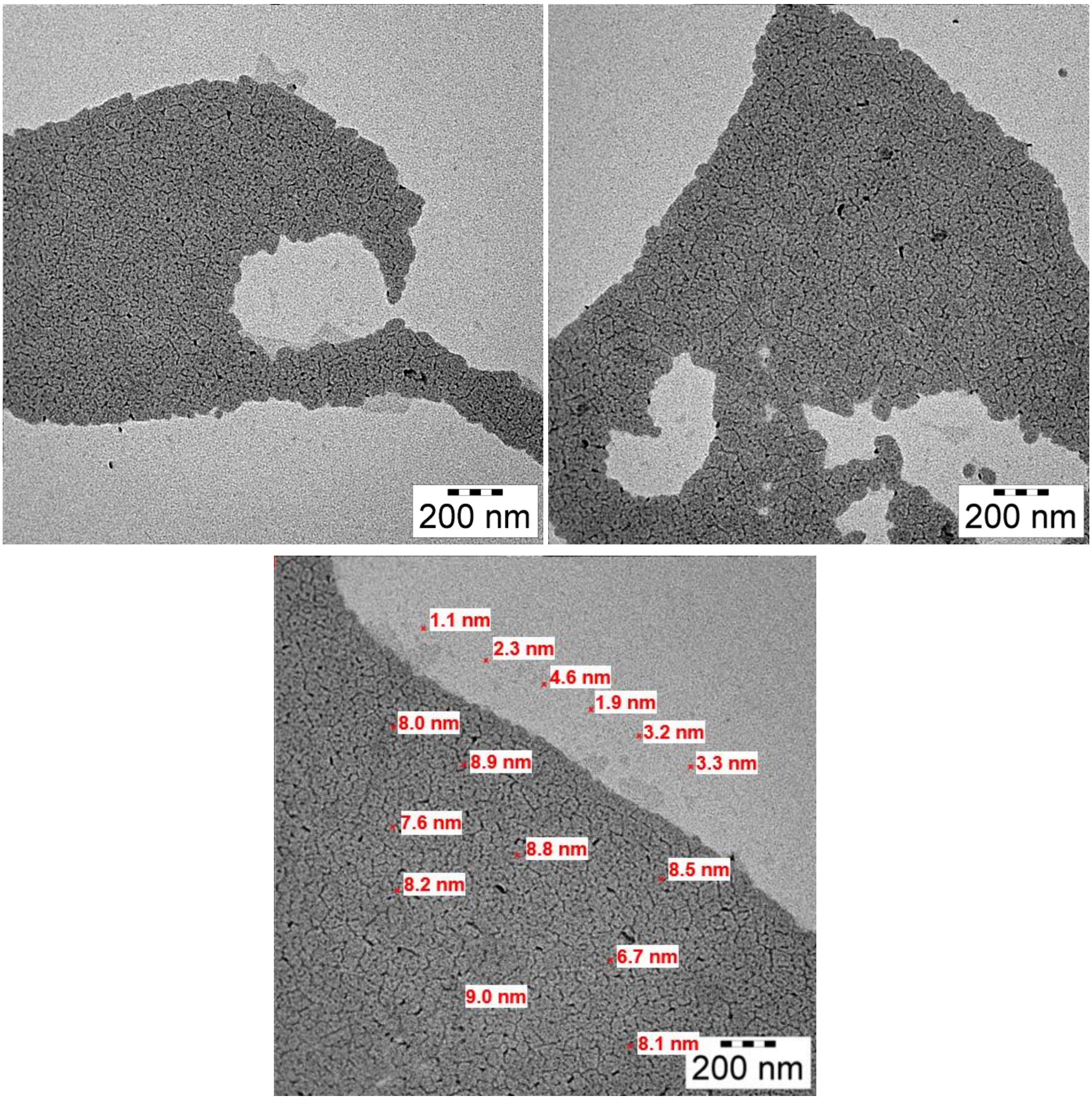

Supplementary Figure 22. Assemblies of nanoparticles obtained in aqueous polymerization (entry 7, Table 2) to layers by drop casting of dialyzed dispersion. Top: Overview images of different assemblies. Bottom: Specimen thickness measurements (red) of typical particle layers determined via TEM. The thickness map was obtained from two acquired images, elastic and global bright-field image (without energy filtering/slit). The mean free path length of carbon was used for all measurements. The average layer thickness was around 6 nm, respectively, around the typical particle thickness as expected for monolayers.

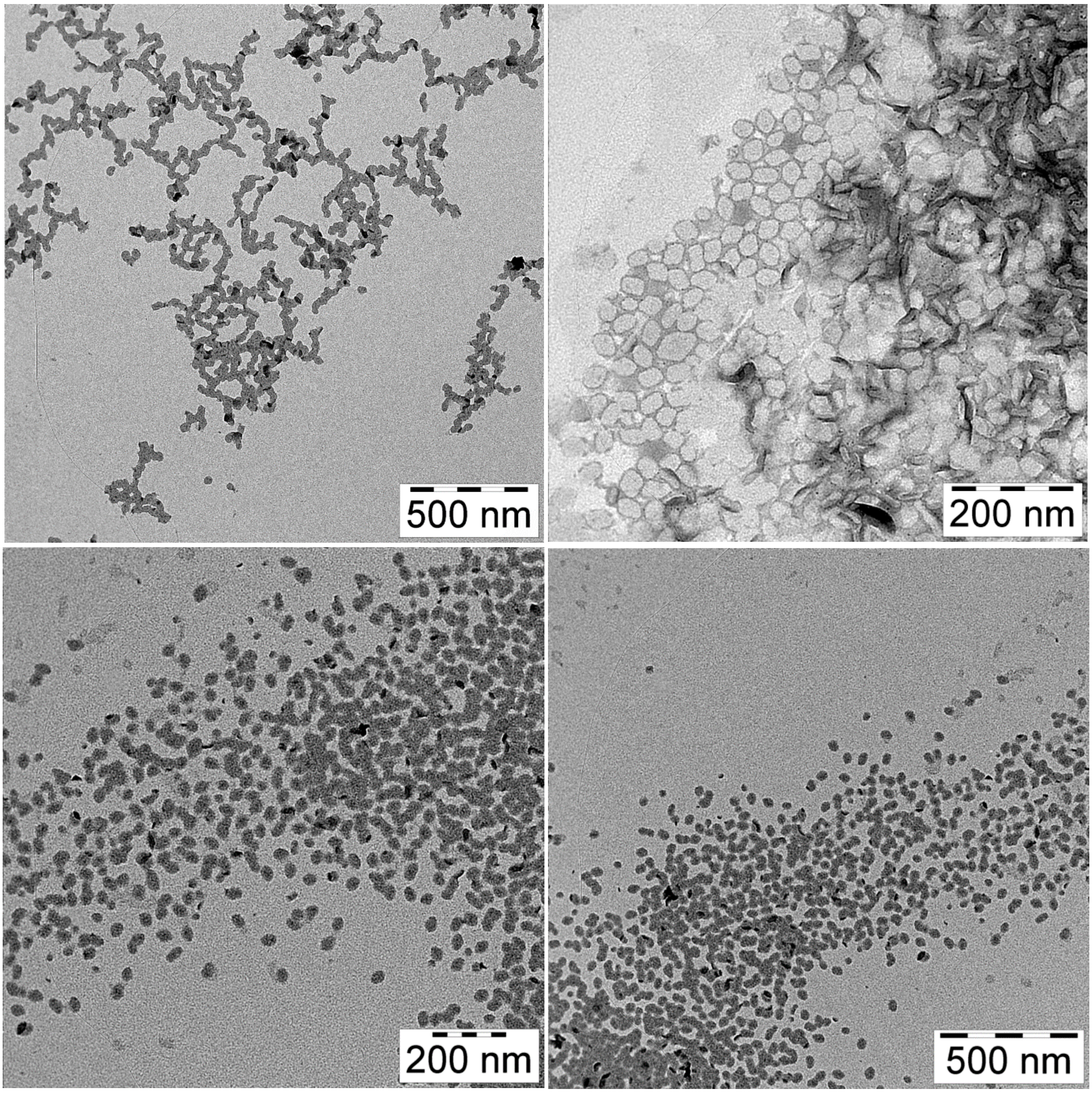

Supplementary Figure 23. Assemblies of nanoparticles obtained in aqueous polymerization (entry 4, Table 2) to layers by drop casting of dialyzed dispersion. Top, left: Low concentrated dispersion was used. No formation of closed layers, but of dendritic structures was observed. Top, right: Higher concentrated dispersion was used. Typically ordered particles were found at the droplet rim together with multilayered structures (black). Bottom: Assemblies obtained from slow drying experiments at lower temperature (5-6 °C). A fluent transition between isolated single particles, small assemblies and layers was observed.

Particle shape analysis

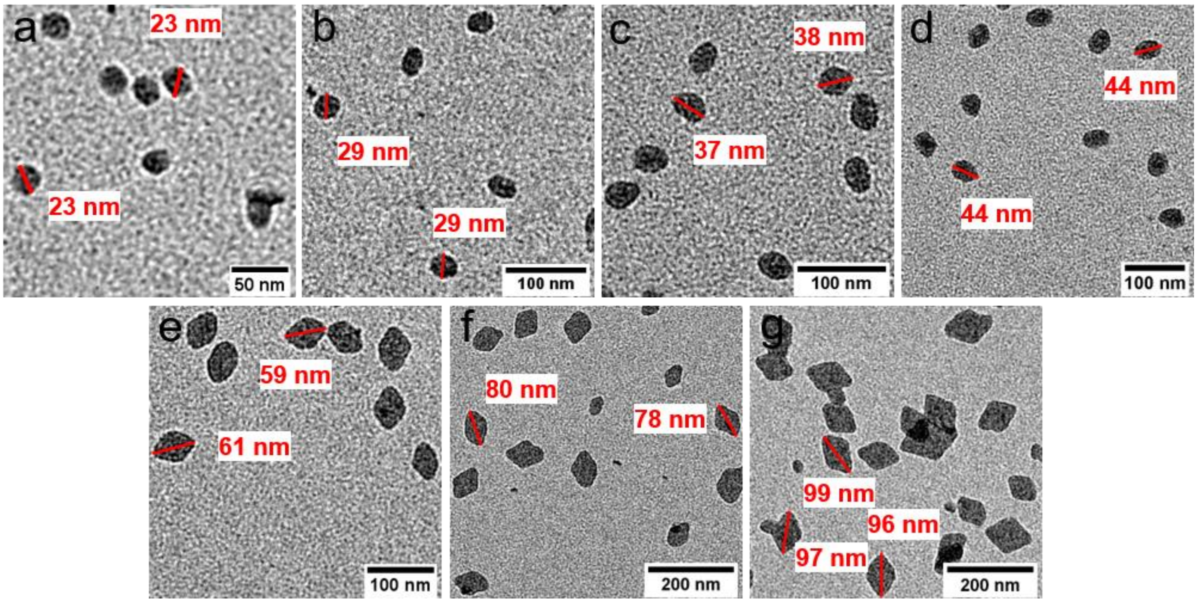

Supplementary Figure 24. TEM images of UHMWPE nanocrystals obtained from aqueous polymerization after different reaction times showing the evolution of size and shape (entries 1-7 (a-g), Supplementary Table 6).

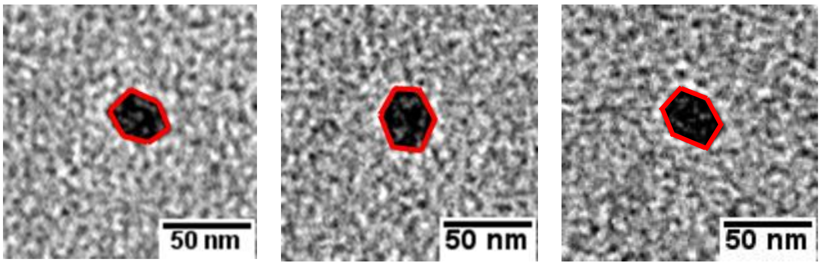

Supplementary Figure 25. Close-up of hexagonal shaped particles obtained in aqueous polymerization (Table 2, entry 2).

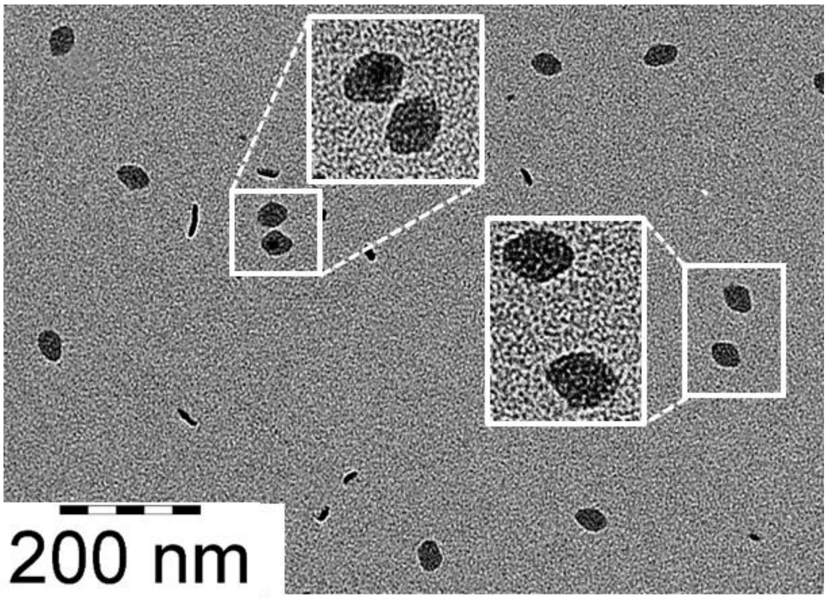

Supplementary Figure 26. Close-up of truncated lozenge shaped particles obtained in aqueous polymerization (Table 2, entry 4). The differences in crystal face growth from hexagonal particles to truncated lozenges are usually identifiable for particles with lateral egde-to-edge sizes >40 nm (particles shown have ~45 nm).

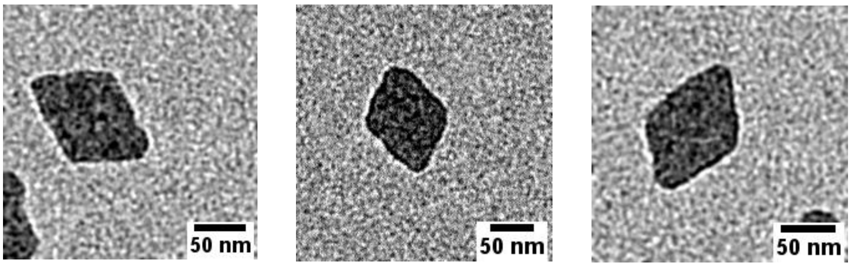

Supplementary Figure 27. Close-up of lozenge shaped particles obtained in aqueous polymerization.

3.3 NMR spectra of complexes

<u>¹H NMR spectra of κ²-(N,O)-Salicylaldiminato Nickel(II) Methyl complexes</u>

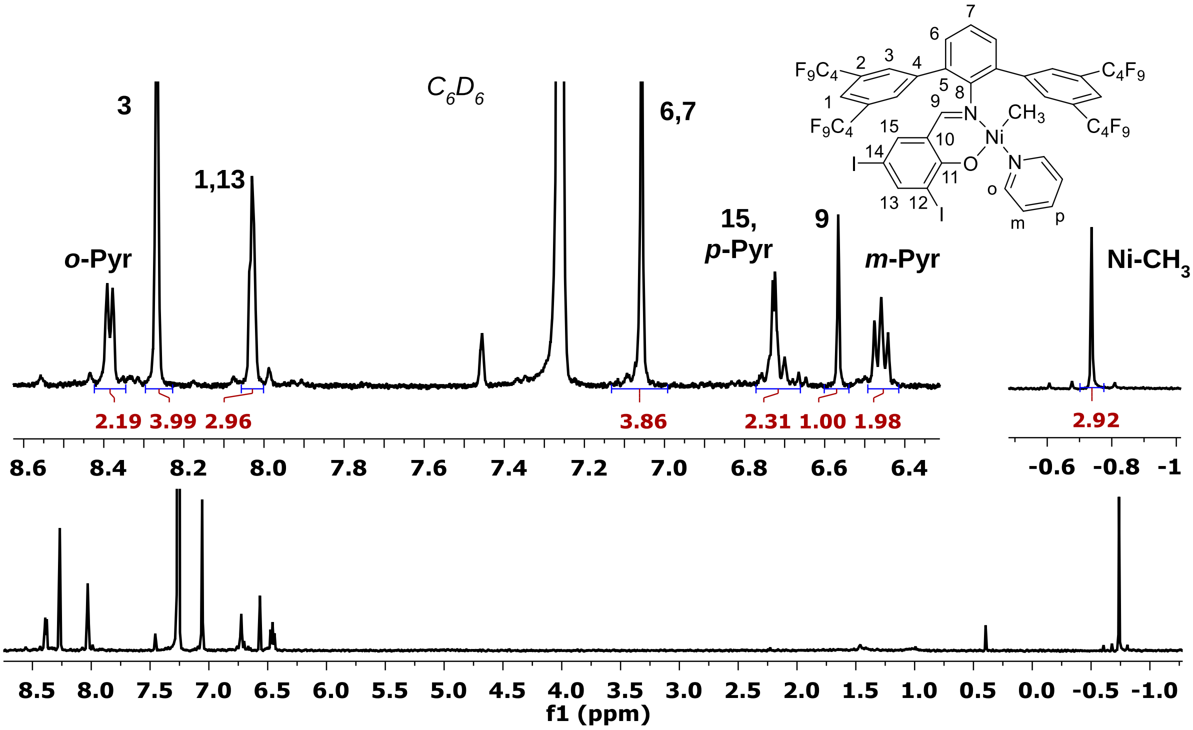

Supplementary Figure 28. ^{1}H NMR spectrum (400 MHz, C_6D_6, 300 K) of complex **1-C_4F_9 /Pyr.**

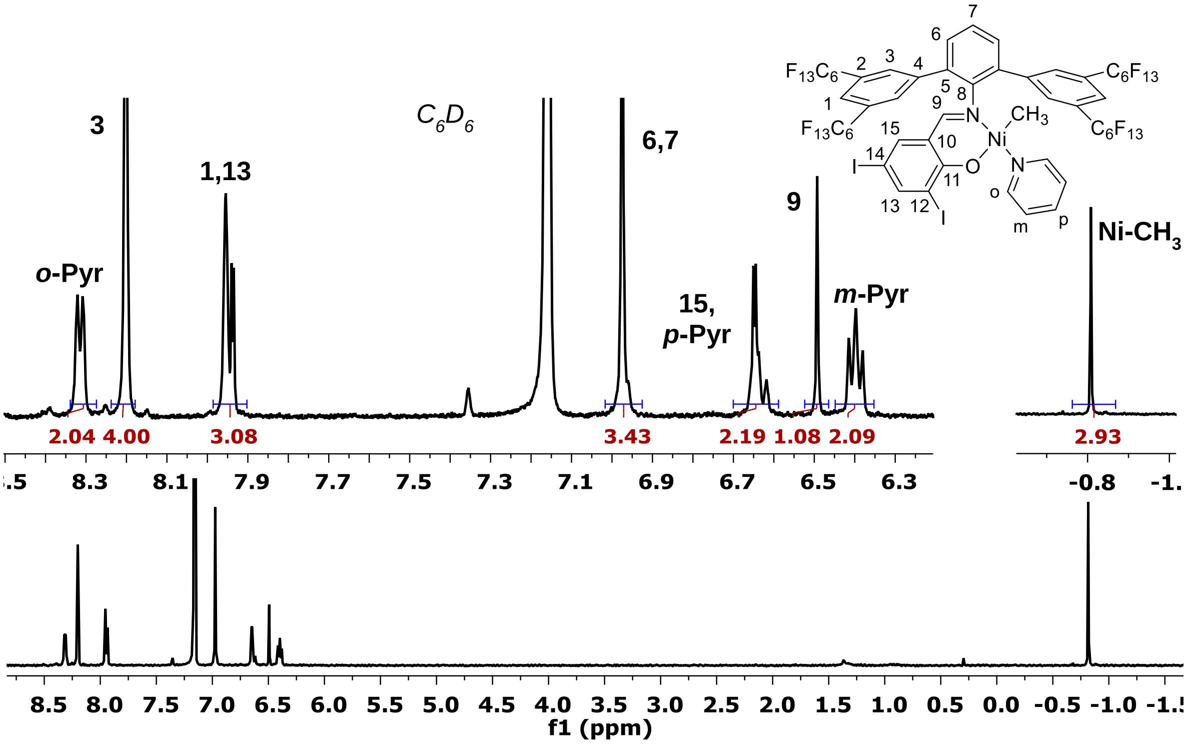

Supplementary Figure 29. ^{1}H NMR spectrum (400 MHz, C_6D_6/C_6F_6, 300 K) of complex **1-C_6F_{13} /Pyr.**

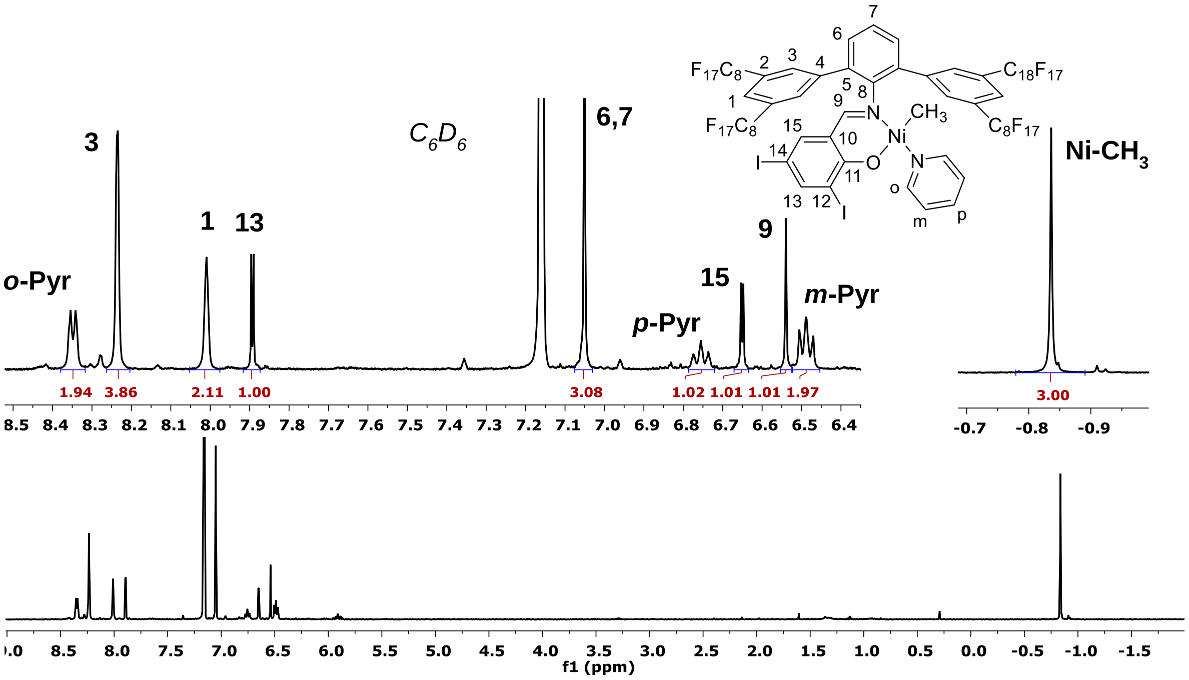

Supplementary Figure 30. ^{1}H NMR spectrum (400 MHz, C_6D_6/C_6F_6, 300 K) of complex **1-C_8F_{17} /Pyr.**

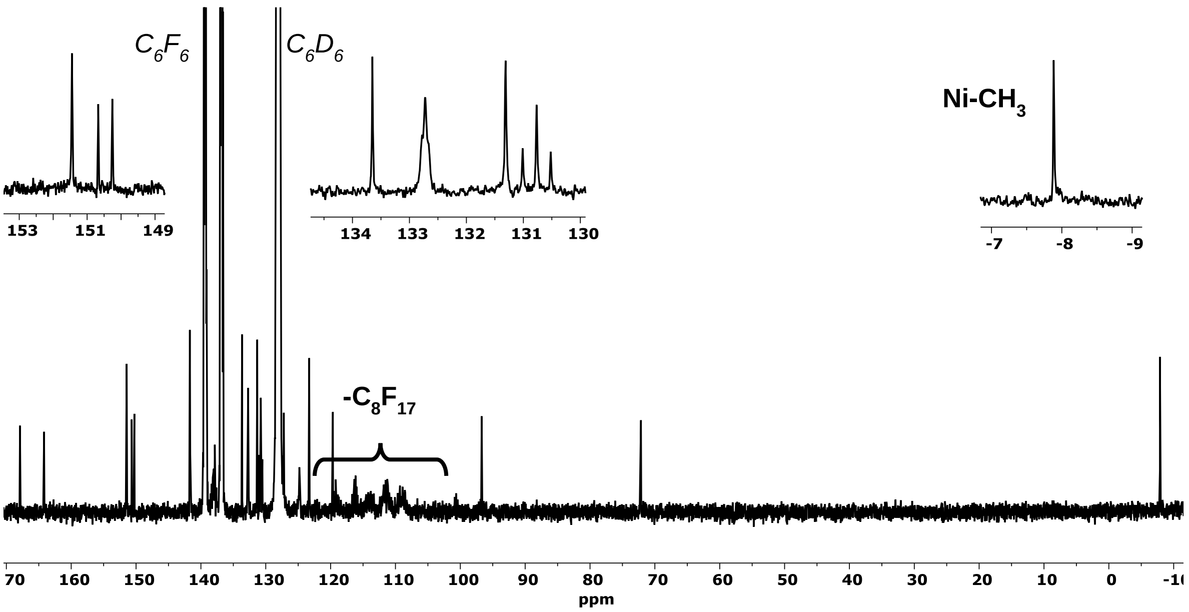

Supplementary Figure 31. ^{13}C{^{1}H} NMR spectrum (400 MHz, C_6D_6/C_6F_6, 300 K) of complex **1-C_8F_{17} /PEG**.

NMR spectra of κ²-(N,O)-Salicylaldiminato Nickel(II) Methyl [α-Methoxy-ω-amino poly(ethylene glycol] complexes

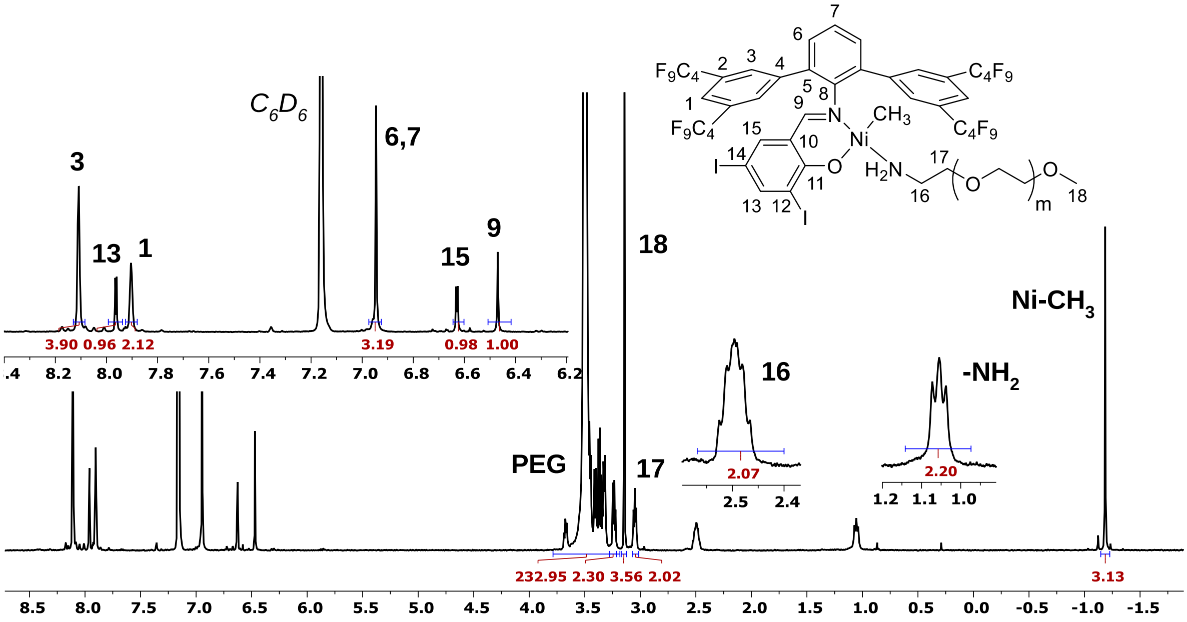

Supplementary Figure 32. ^{1}H NMR spectrum (400 MHz, C_6D_6, 300 K) of complex **1-C_4F_9 /PEG.**

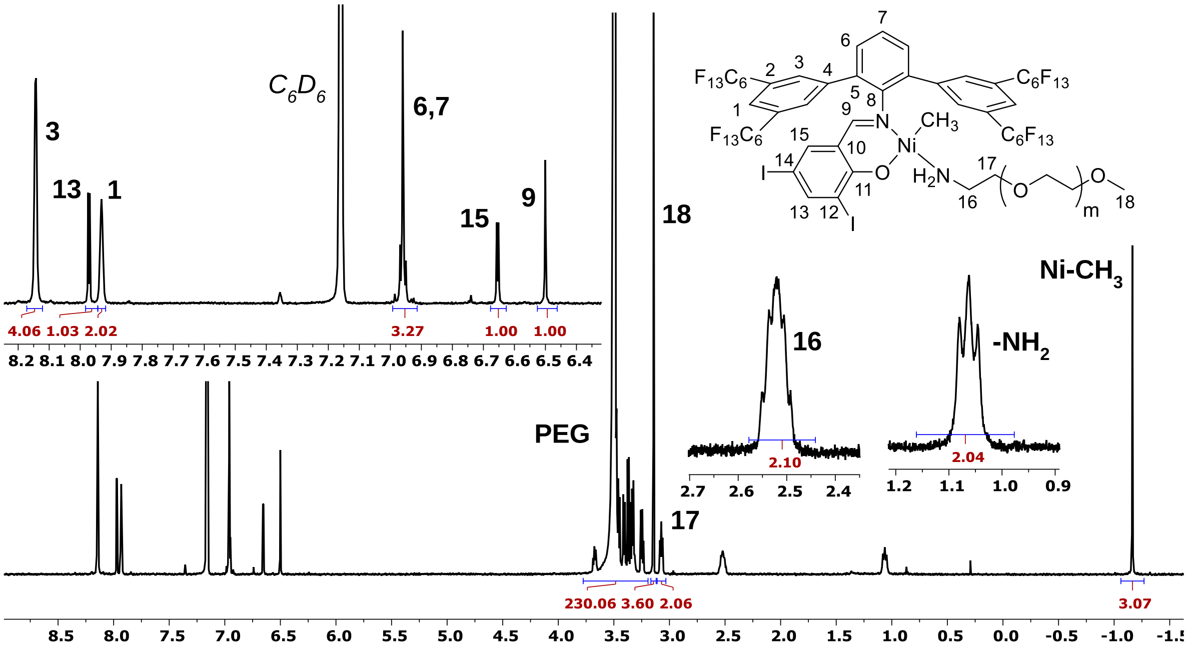

Supplementary Figure 33. ^{1}H NMR spectrum (400 MHz, C_6D_6, 300 K) of complex **1-C_6F_{13} /PEG**.

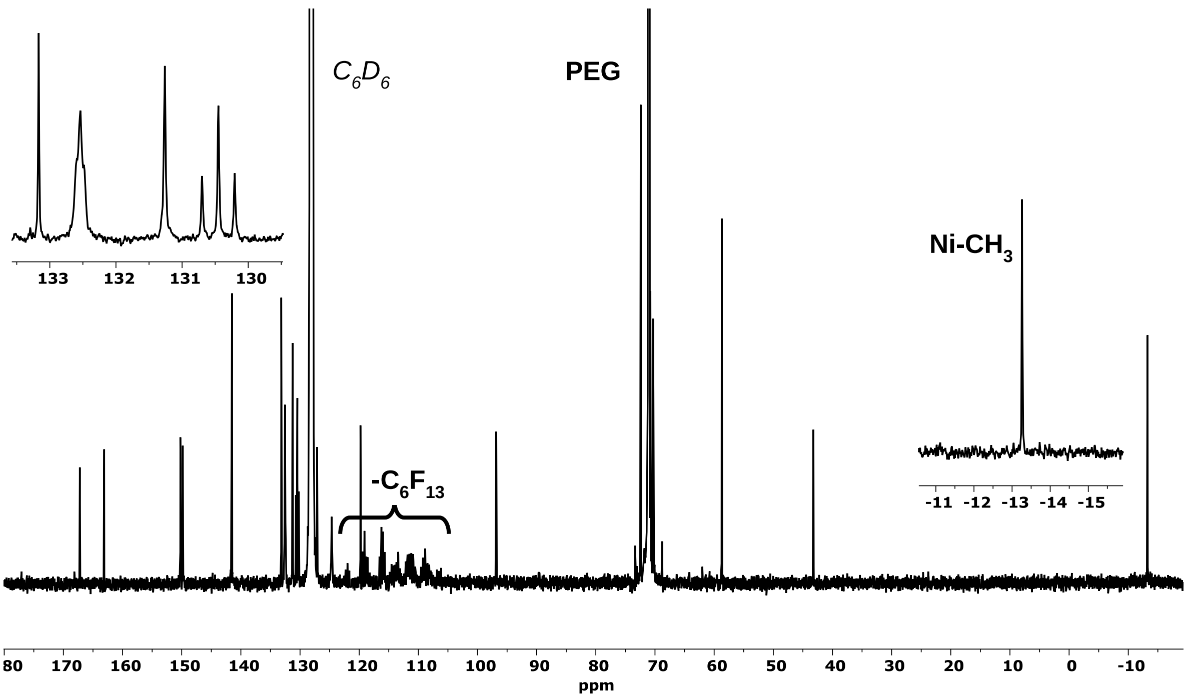

Supplementary Figure 34. $^{13}C\{^{1}H\}$ NMR spectrum (400 MHz, C_6D_6, 300 K) of complex **1-C_6F_{13}/PEG**.

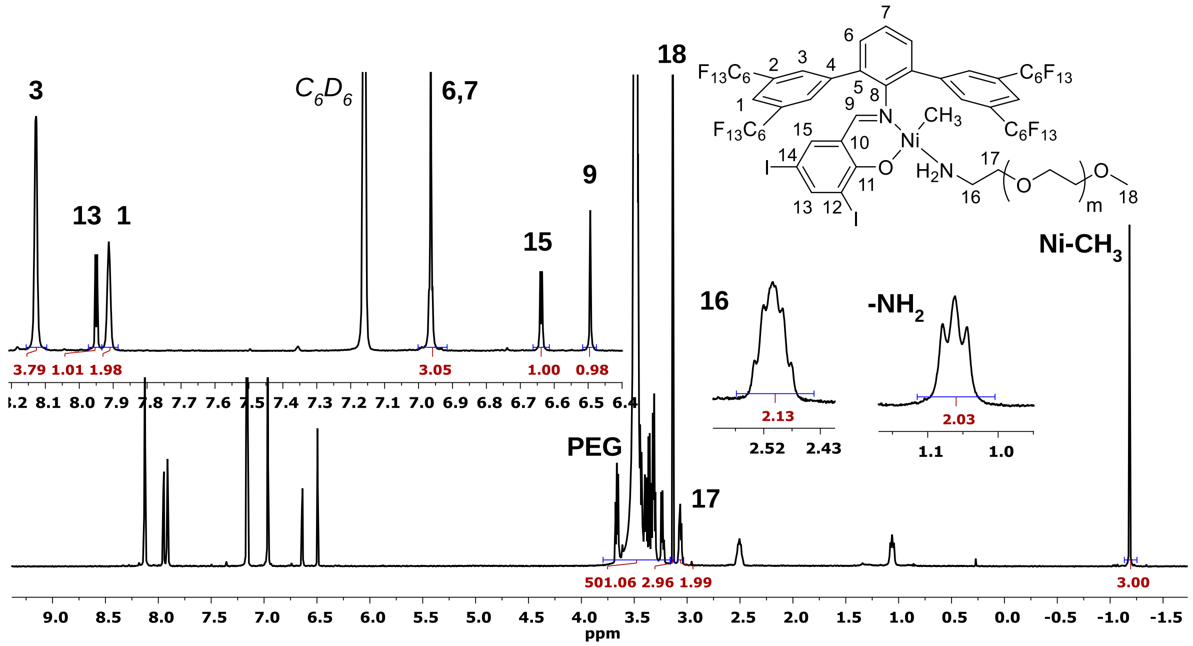

Supplementary Figure 35. ^{1}H NMR spectrum (400 MHz, C_6D_6, 300 K) of complex **1-C_6F_{13}/PEG** with M(H_2N-PEG-OMe) = 5516 g mol^{-1}**.**

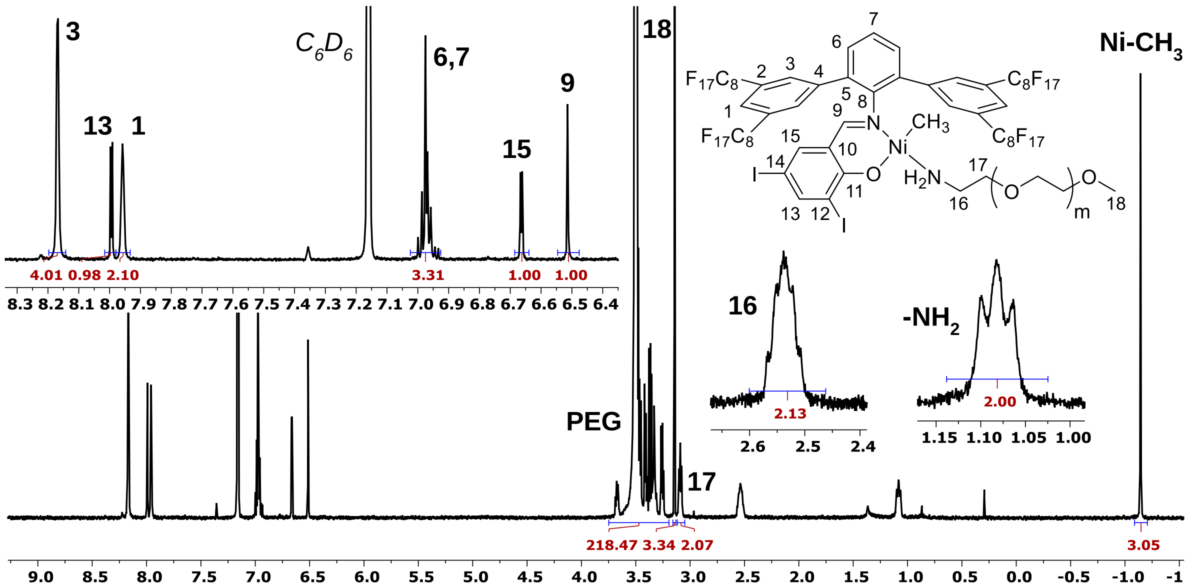

Supplementary Figure 36. ^{1}H NMR spectrum (400 MHz, C_6D_6, 300 K) of complex **1-C_8F_{17}/PEG.**

3.4 GPC traces of synthesized polyethylenes

MW Averages

Mp: 535567	Mn: 274278	Mv: 480723	Mw: 514252
Mz: 769068	Mz+1: 1020335	PD: 1.8749	

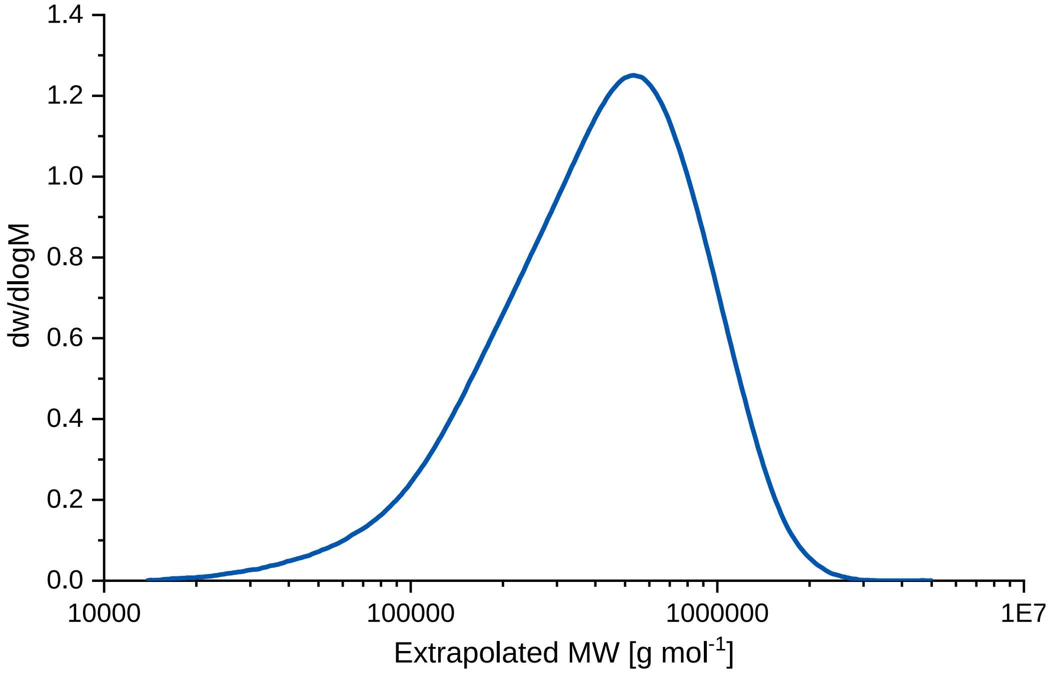

Supplementary Figure 37. GPC trace of polyethylene obtained from polymerization in toluene (Table 1, entry 1).

MW Averages

Mp: 677336	Mn: 488544	Mv: 684305	Mw: 716460
Mz: 967941	Mz+1: 1225414	PD: 1.4665	

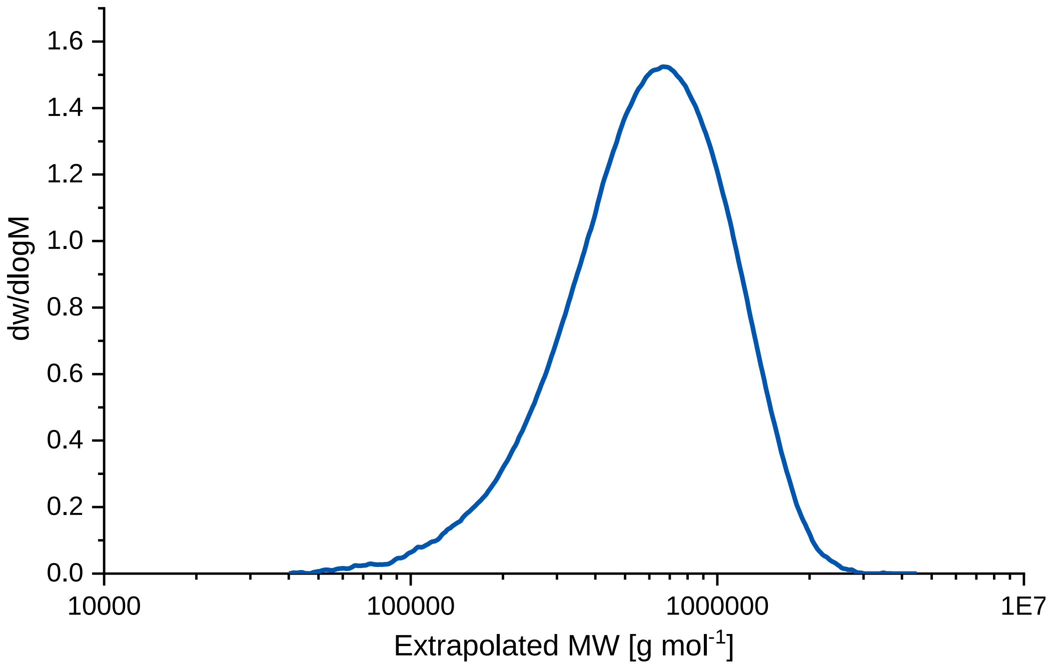

Supplementary Figure 38. GPC trace of polyethylene obtained from polymerization in toluene (Table 1, entry 4).

MW Averages

Mp: 1290184 Mn: 1189881 Mv: 1451560 Mw: 1498582

Mz: 1841984 Mz+1: 2205834 PD: 1.2594

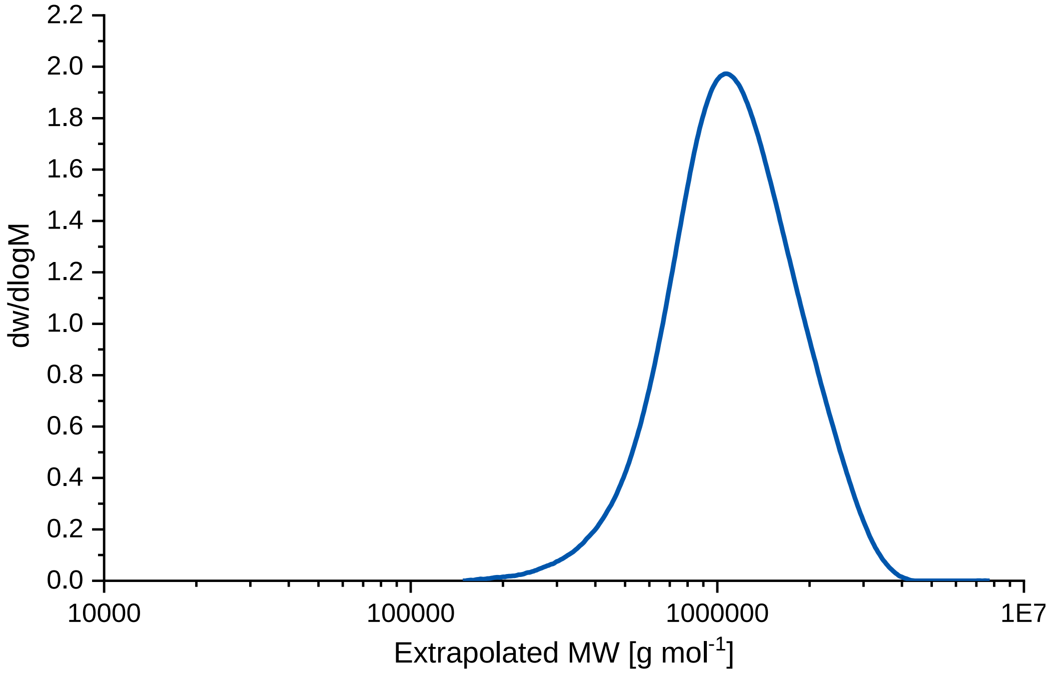

Supplementary Figure 39. GPC trace of polyethylene obtained from polymerization in toluene (Table 1, entry 6).

MW Averages

Mp: 1040276 Mn: 844265 Mv: 1107594 Mw: 1153173

Mz: 1488868 Mz+1: 1828966 PD: 1.3659

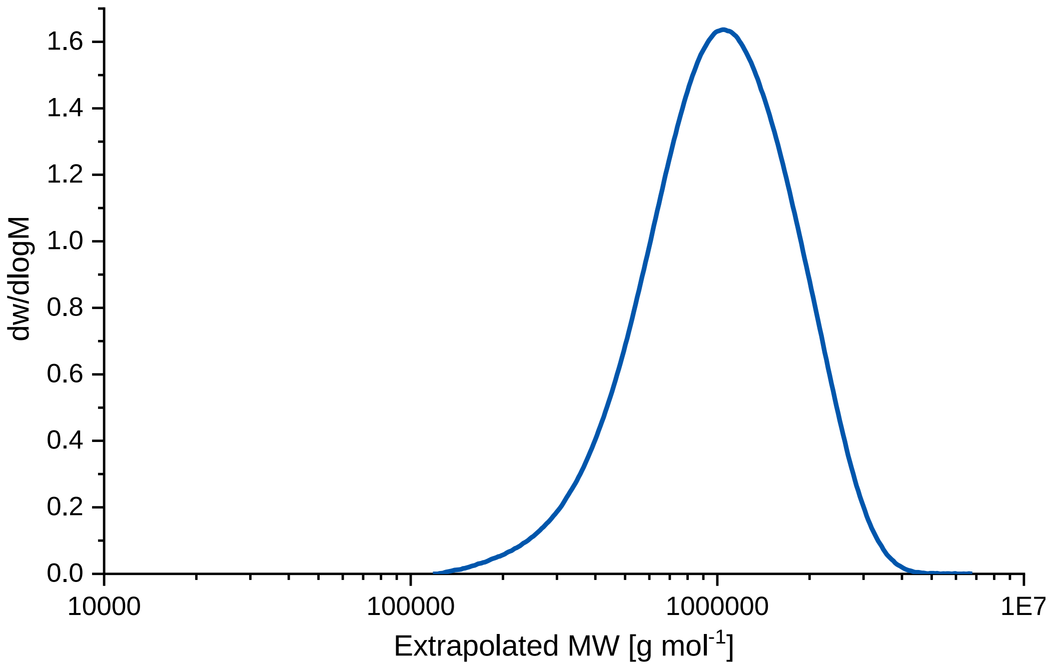

Supplementary Figure 40. GPC trace of polyethylene obtained from polymerization in toluene (Table 1, entry 7).

MW Averages

Mp: 669276	Mn: 514505	Mv: 711657	Mw: 745849
Mz: 1001982	Mz+1: 1256795	PD: 1.4496	

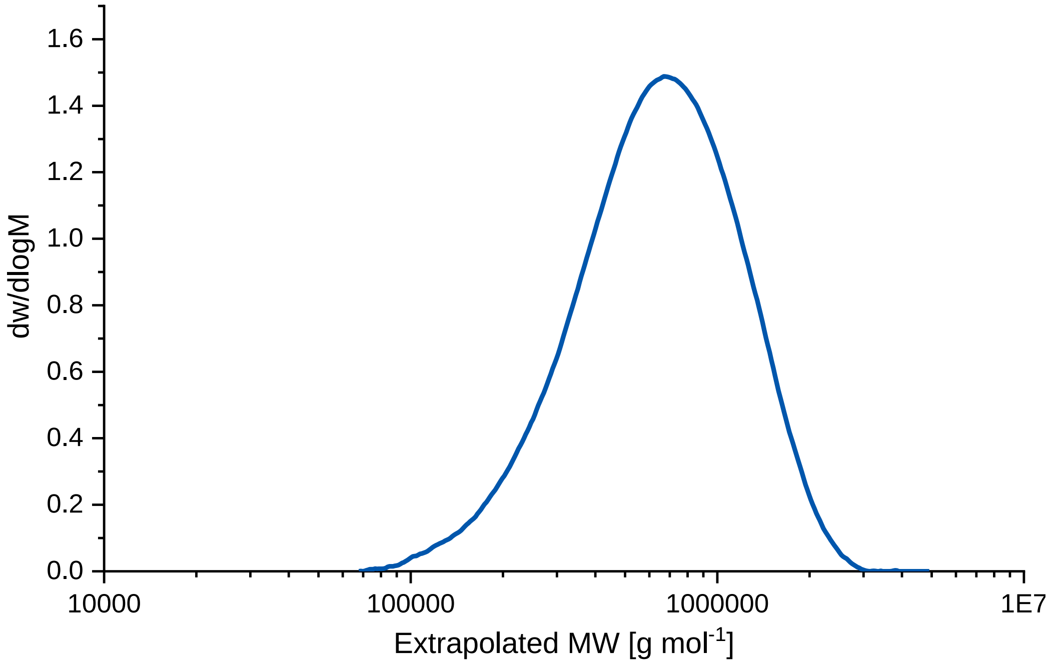

Supplementary Figure 41. GPC trace of polyethylene obtained from polymerization in toluene (Table 1, entry 11).

MW Averages

Mp: 841367	Mn: 631128	Mv: 744627	Mw: 759766
Mz: 858675	Mz+1: 939230	PD: 1.2038	

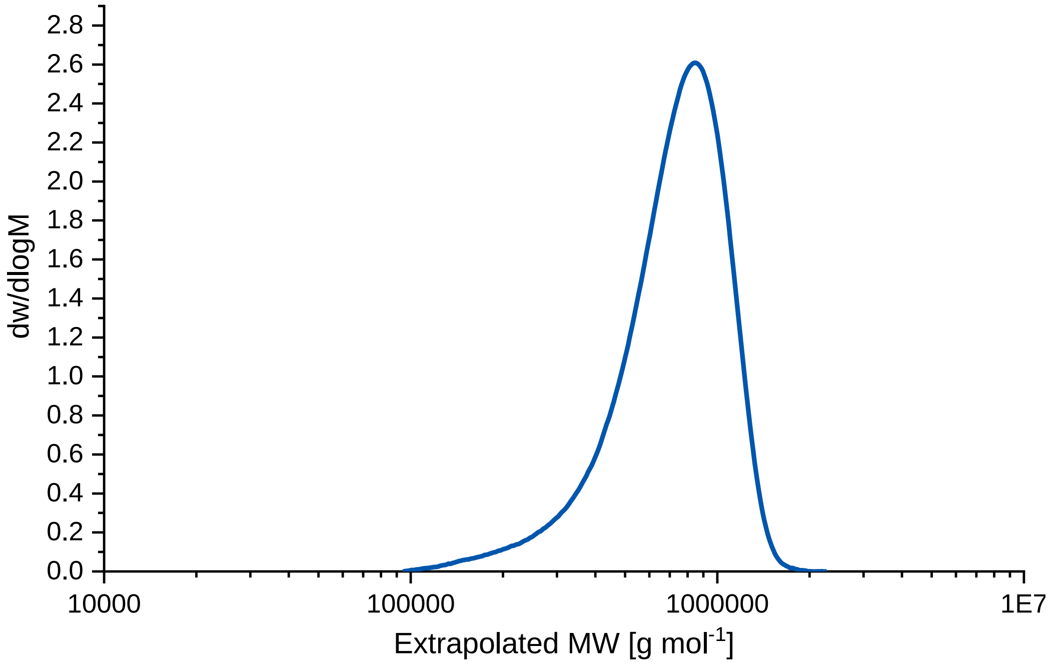

Supplementary Figure 42. GPC trace of polyethylene obtained in aqueous polymerization (Table 2, entry 1).

MW Averages

Mp: 1119626	Mn: 895720	Mv: 1061798	Mw: 1086858
Mz: 1256537	Mz+1: 1407631	PD: 1.2134	

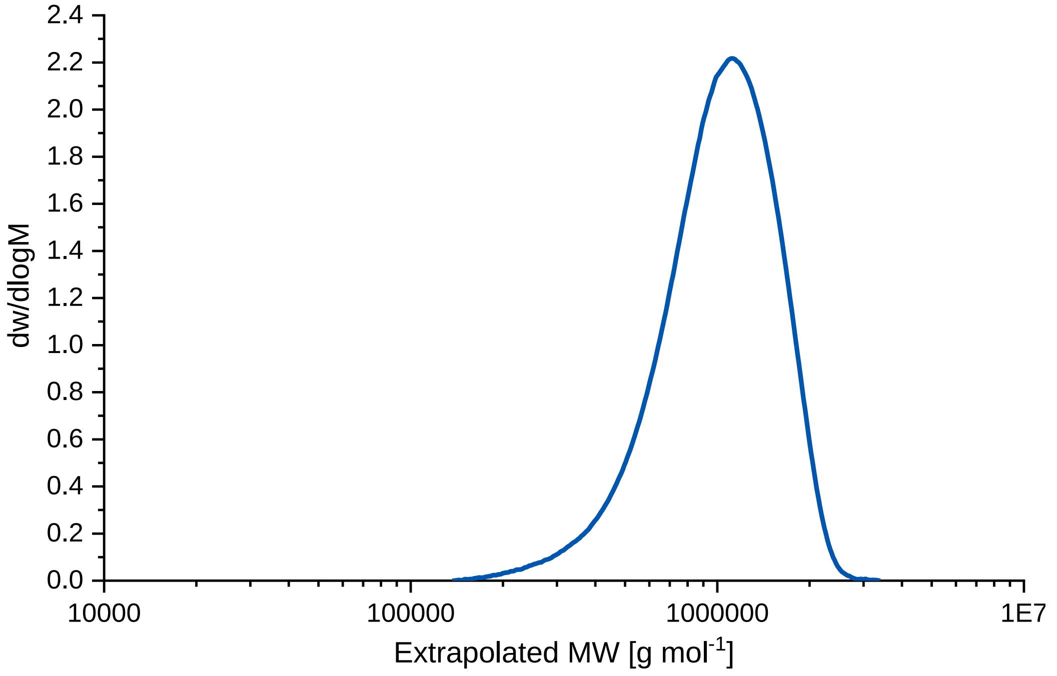

Supplementary Figure 43. GPC trace of polyethylene obtained in aqueous polymerization (Table 2, entry 2).

MW Averages

Mp: 1293036	Mn: 1156651	Mv: 1368421	Mw: 1402375
Mz: 1654788	Mz+1: 1895446	PD: 1.2124	

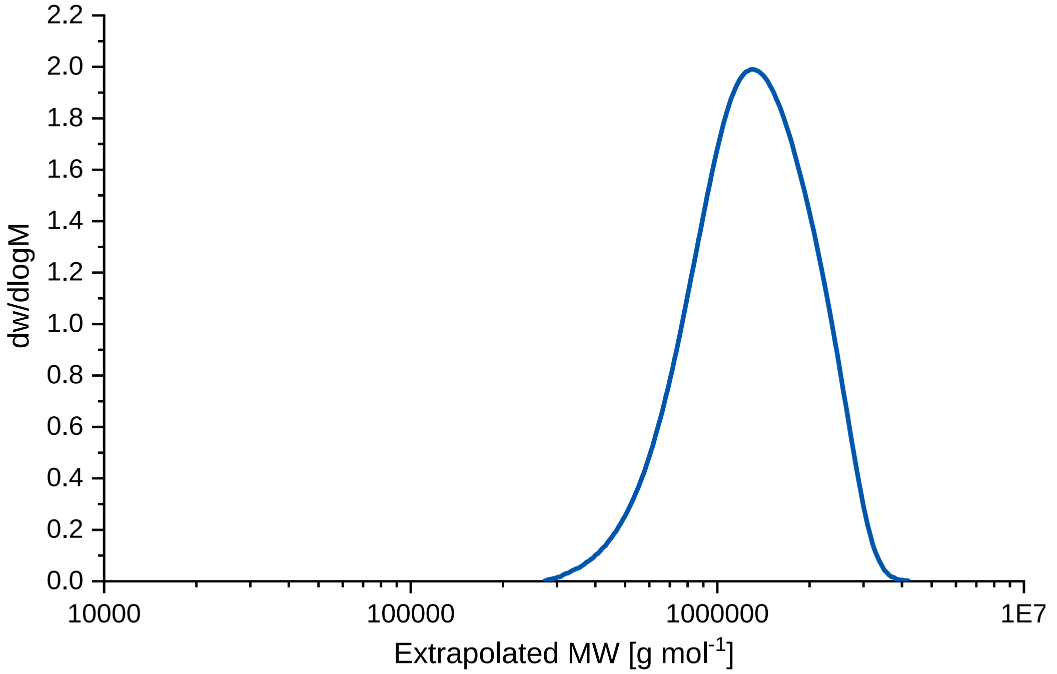

Supplementary Figure 44. GPC trace of polyethylene obtained in aqueous polymerization (Table 2, entry 3).

MW Averages

Mp: 1614059	Mn: 1420795	Mv: 1689964	Mw: 1735217
Mz: 2058946	Mz+1: 2374972	PD: 1.2213	

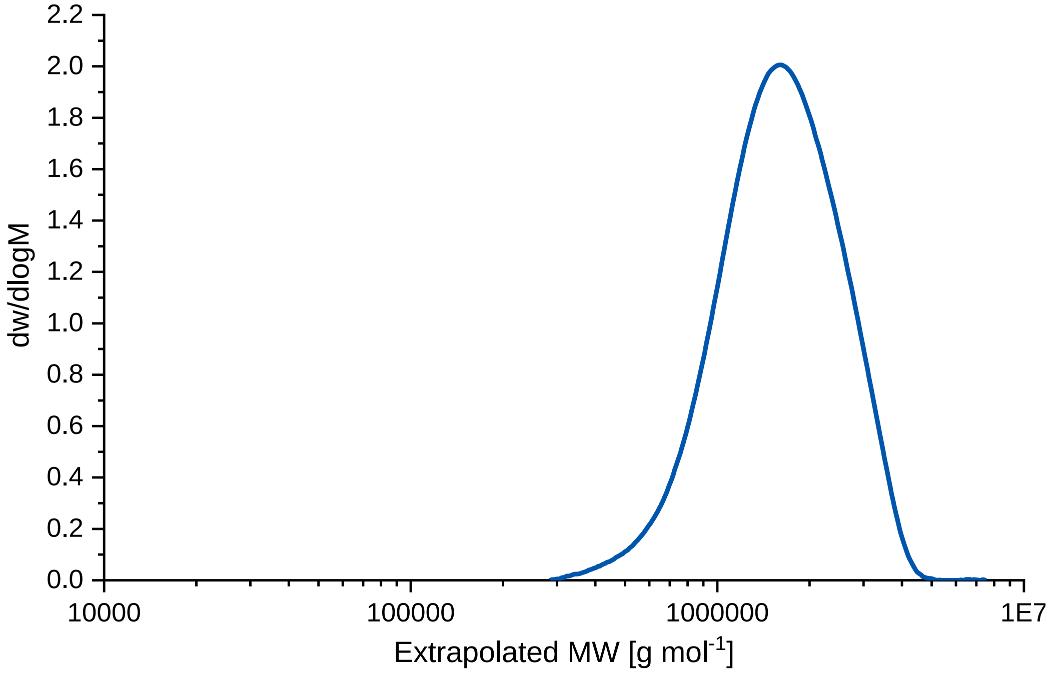

Supplementary Figure 45. GPC trace of polyethylene obtained in aqueous polymerization (Table 2, entry 4).

MW Averages

Mp: 1935030	Mn: 1911985	Mv: 2267010	Mw: 2370536
Mz: 2926073	Mz+1: 3529468	PD: 1.2398	

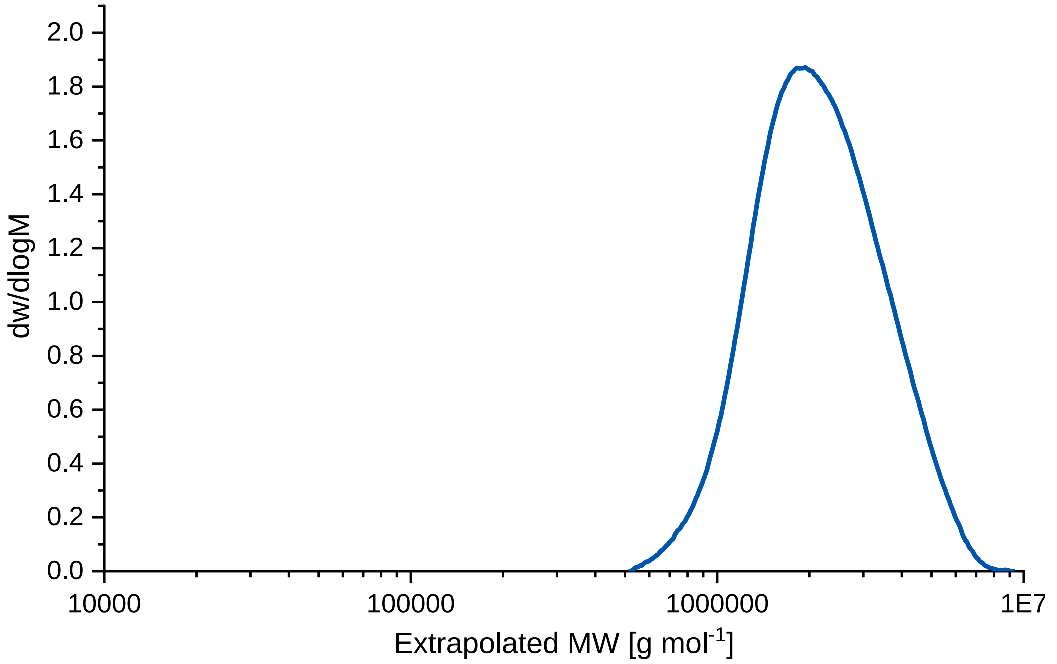

Supplementary Figure 46. GPC trace of polyethylene obtained in aqueous polymerization (Table 2, entry 5).

MW Averages

Mp: 2488346	Mn: 2588220	Mv: 3056994	Mw: 3202100
Mz: 3967207	Mz+1: 4812901	PD: 1.2372	

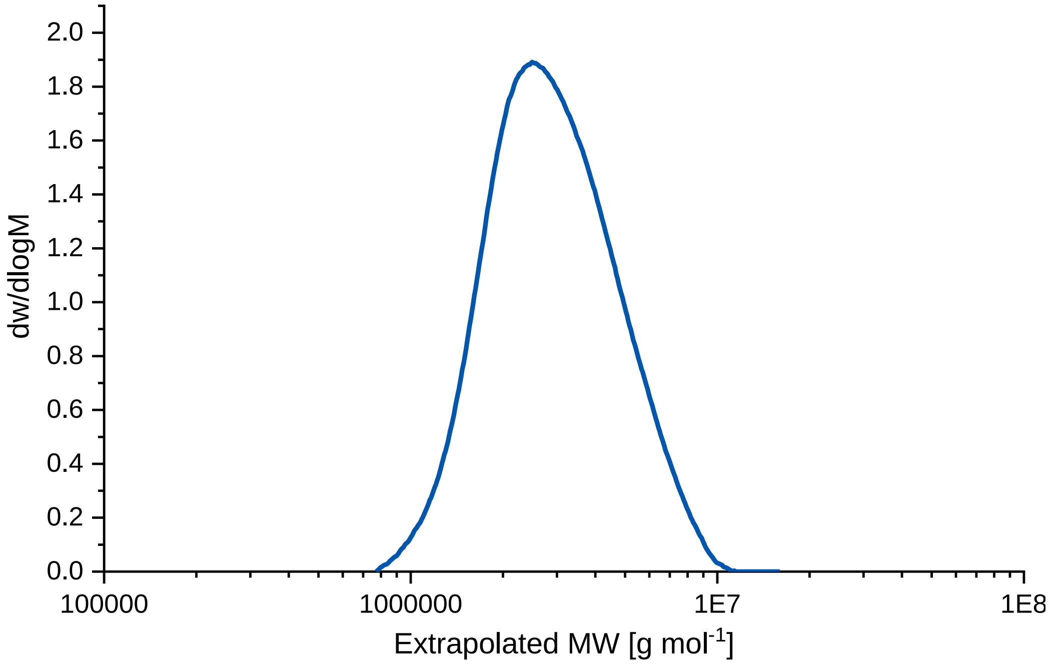

Supplementary Figure 47. GPC trace of polyethylene obtained in aqueous polymerization (Table 2, entry 6).

MW Averages

Mp: 3079988	Mn: 3083878	Mv: 3743773	Mw: 3952007
Mz: 4989235	Mz+1: 6093629	PD: 1.2815	

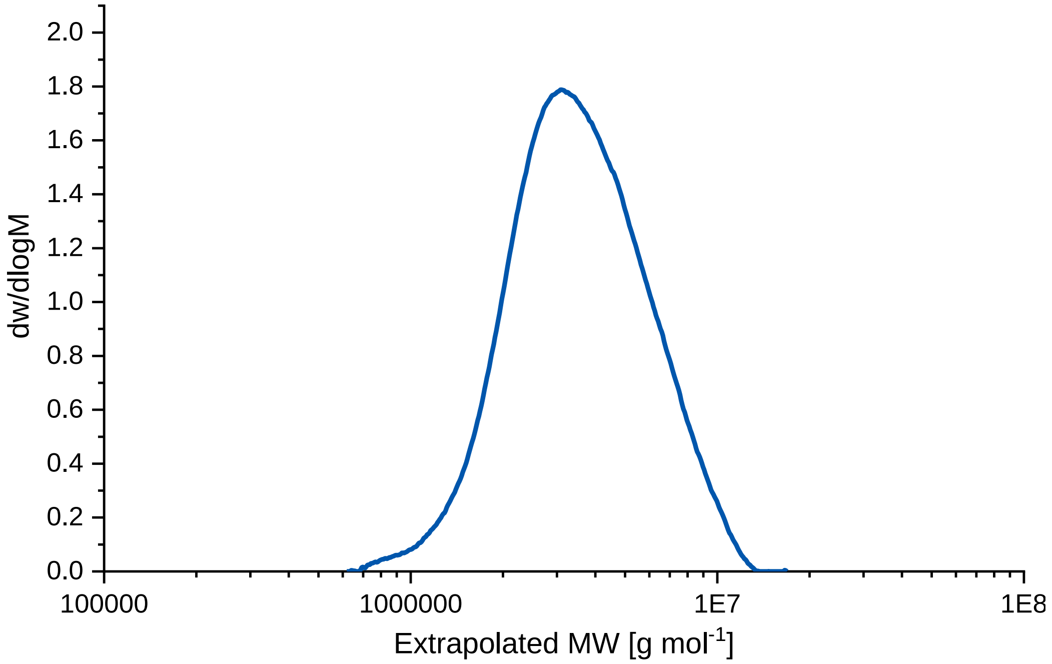

Supplementary Figure 48. GPC trace of polyethylene obtained in aqueous polymerization (Table 2, entry 7).

3.5 DSC traces of polyethylenes obtained in aqueous polymerization

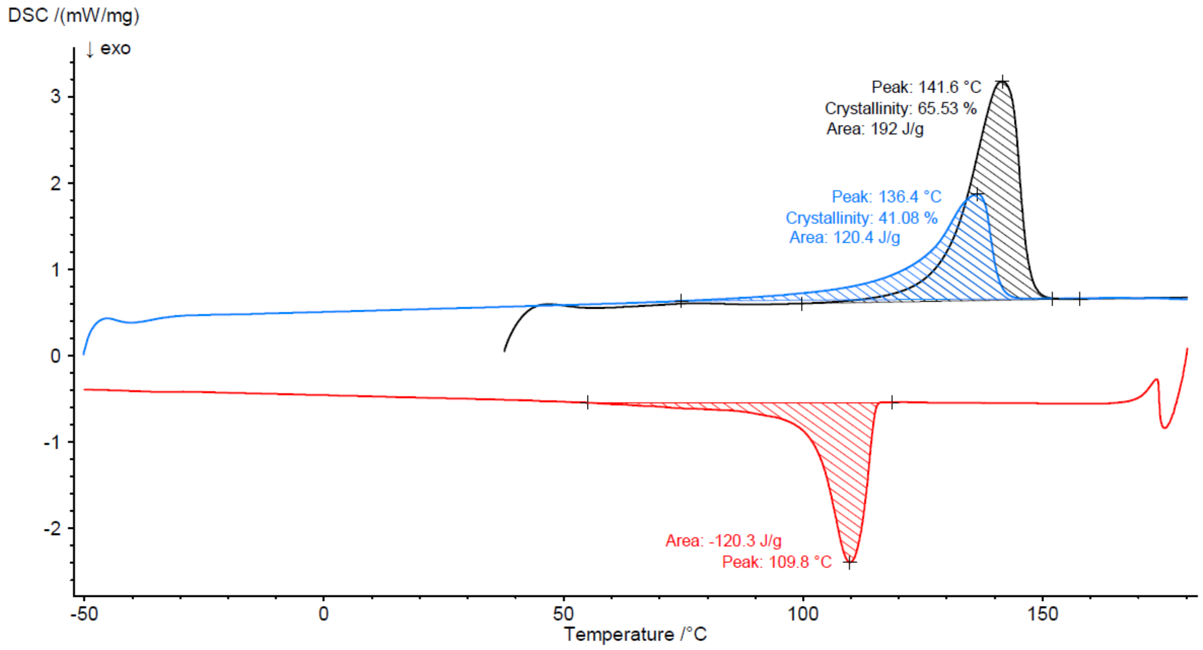

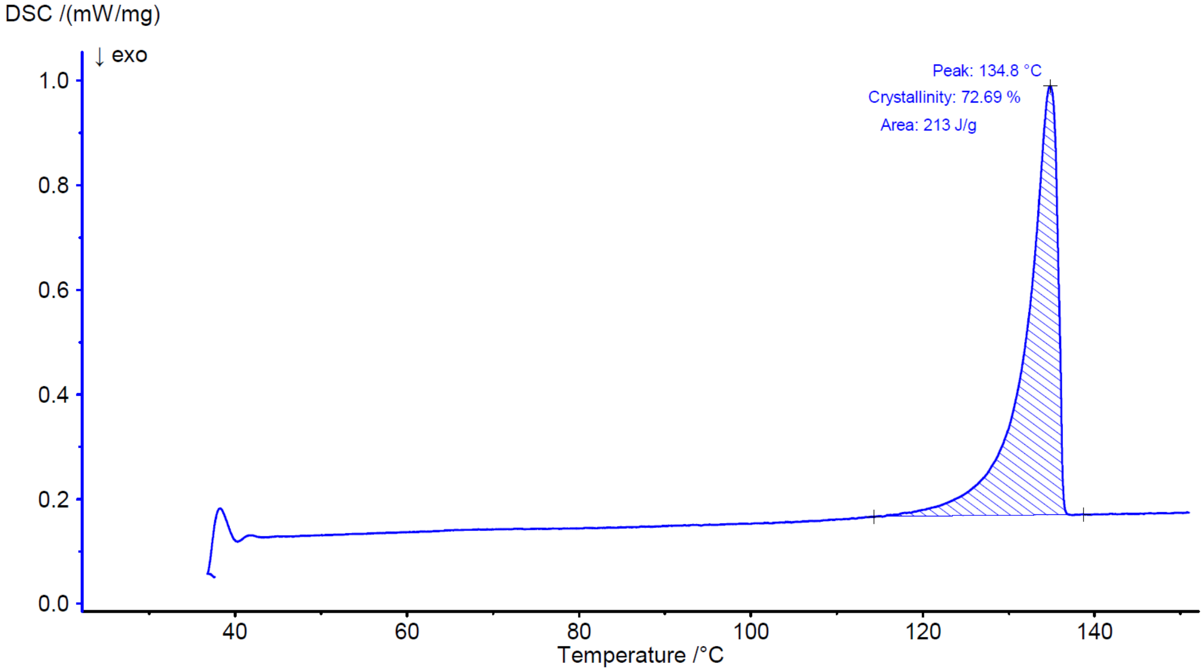

Supplementary Figure 49. DSC traces of polyethylene obtained by aqueous polymerization (Table 2, entry 1). Top: measured with 10 K min^{-1} heating rate (black curve: 1st heating, red curve: 1st cooling, blue curve: 2nd heating). Bottom: measured with 1 K min^{-1} (only first heating shown).

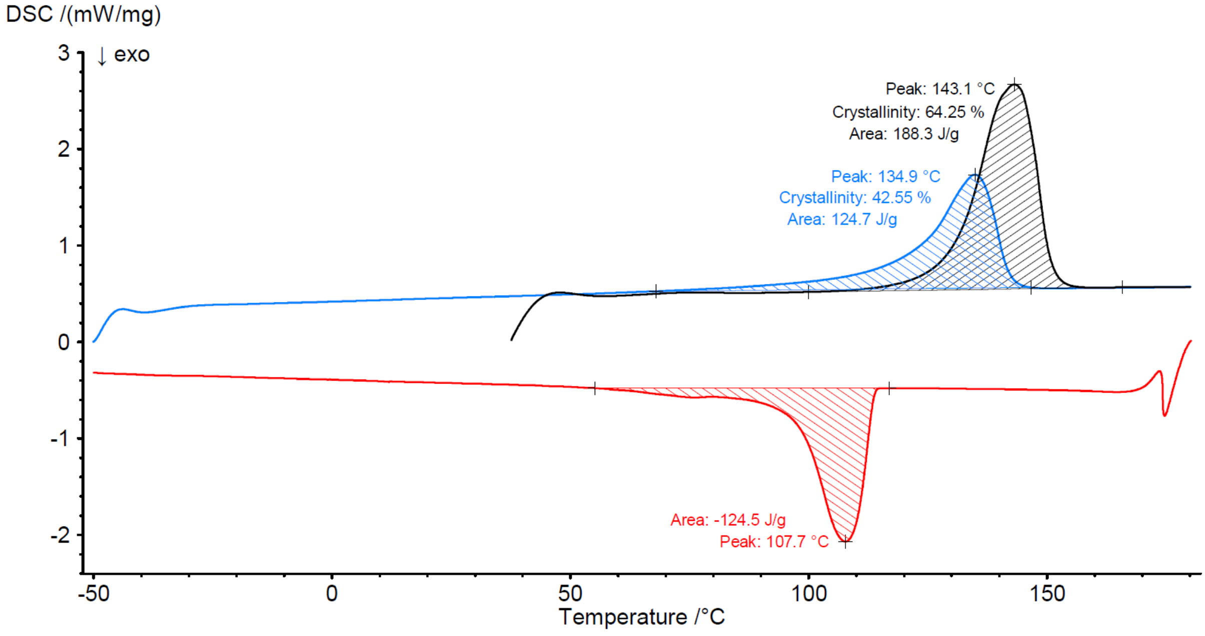

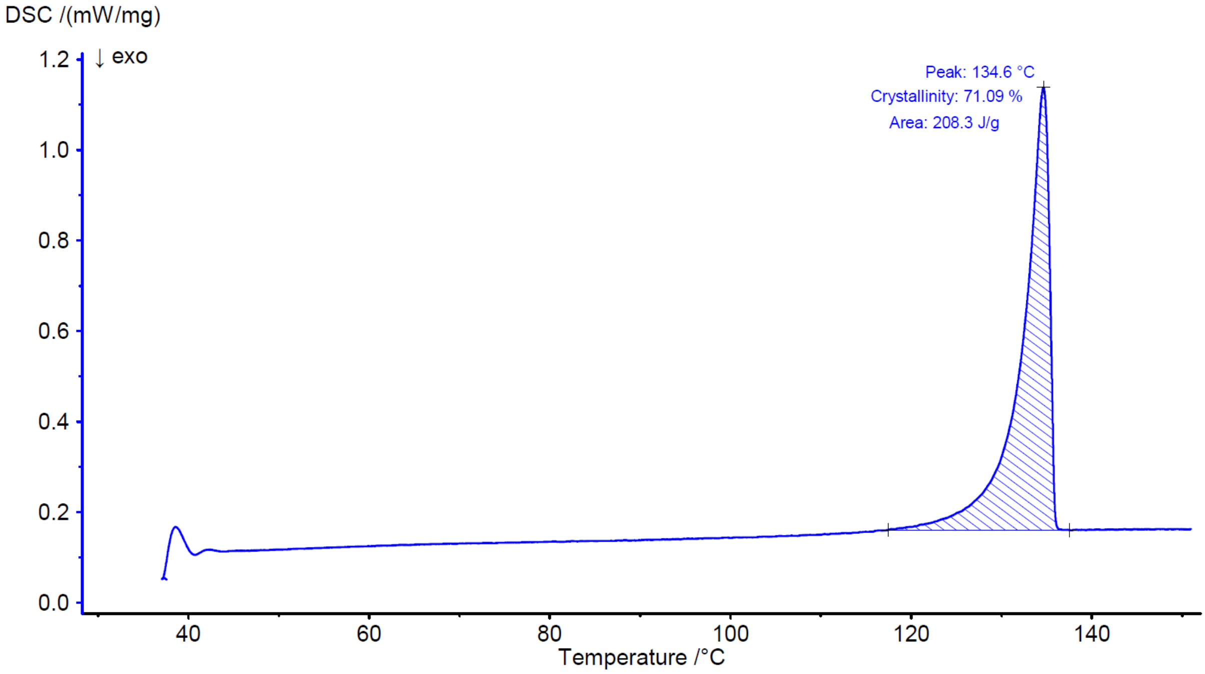

Supplementary Figure 50. DSC traces of polyethylene obtained by aqueous polymerization (Table 2, entry 2). Top: measured with 10 K min^{-1} heating rate (black curve: 1st heating, red curve: 1st cooling, blue curve: 2nd heating). Bottom: measured with 1 K min^{-1} (only first heating shown).

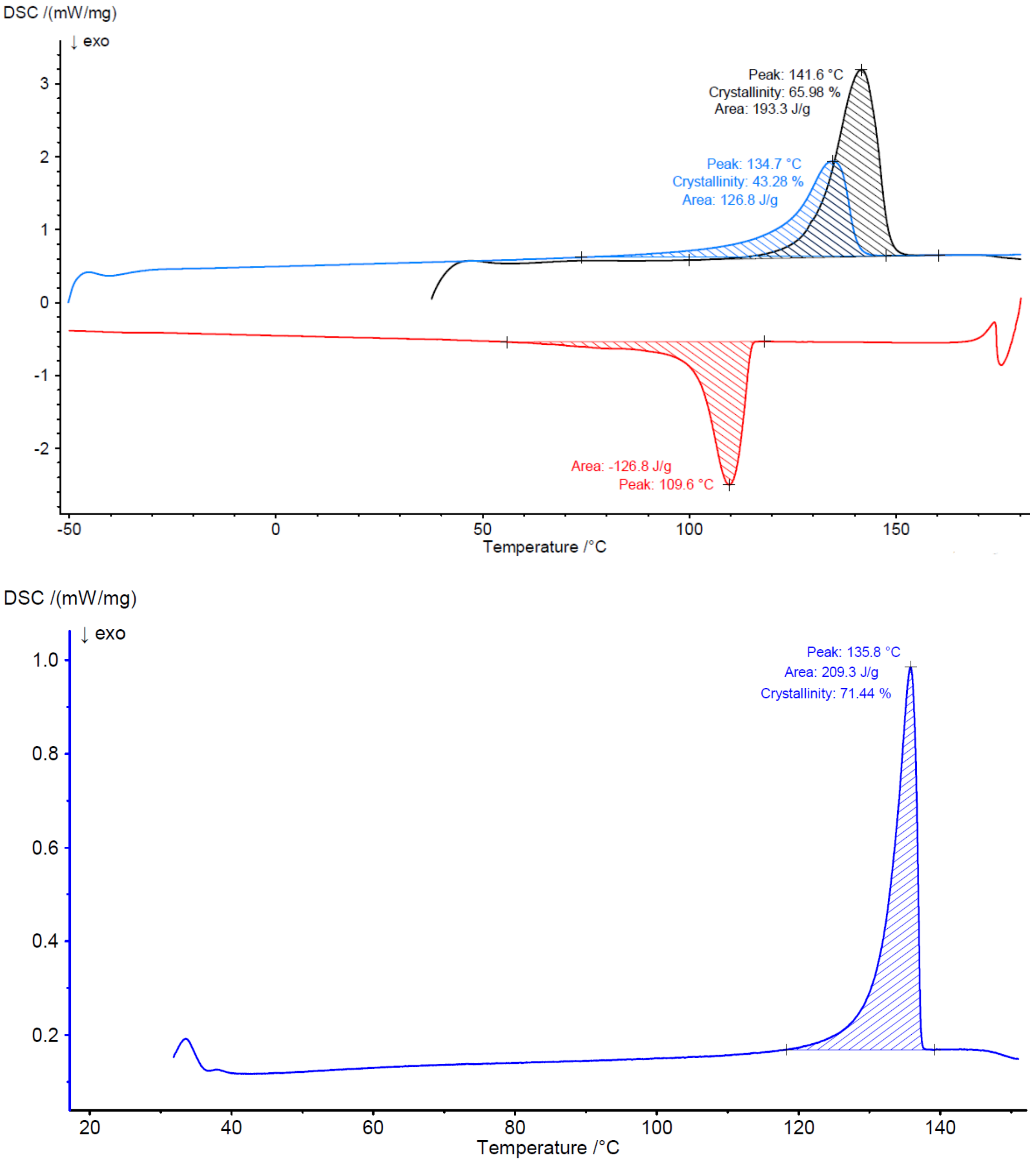

Supplementary Figure 51. DSC traces of polyethylene obtained by aqueous polymerization (Table 2, entry 3). Top: measured with 10 K min^{-1} heating rate (black curve: 1st heating, red curve: 1st cooling, blue curve: 2nd heating). Bottom: measured with 1 K min^{-1} (only first heating shown).

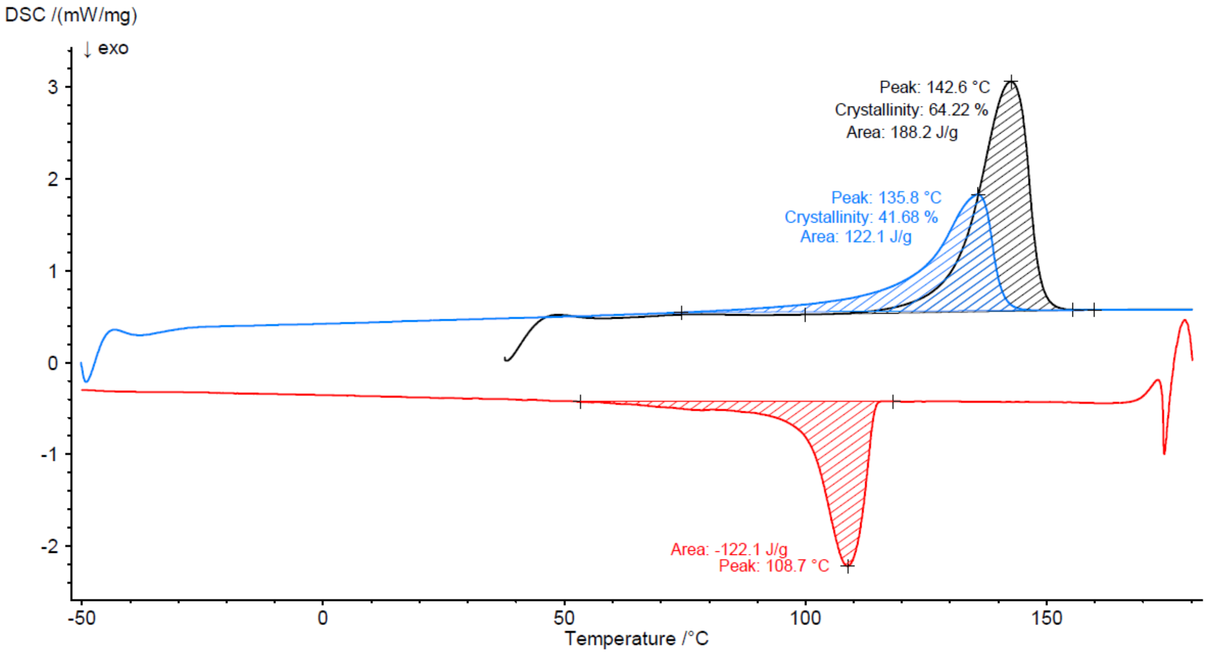

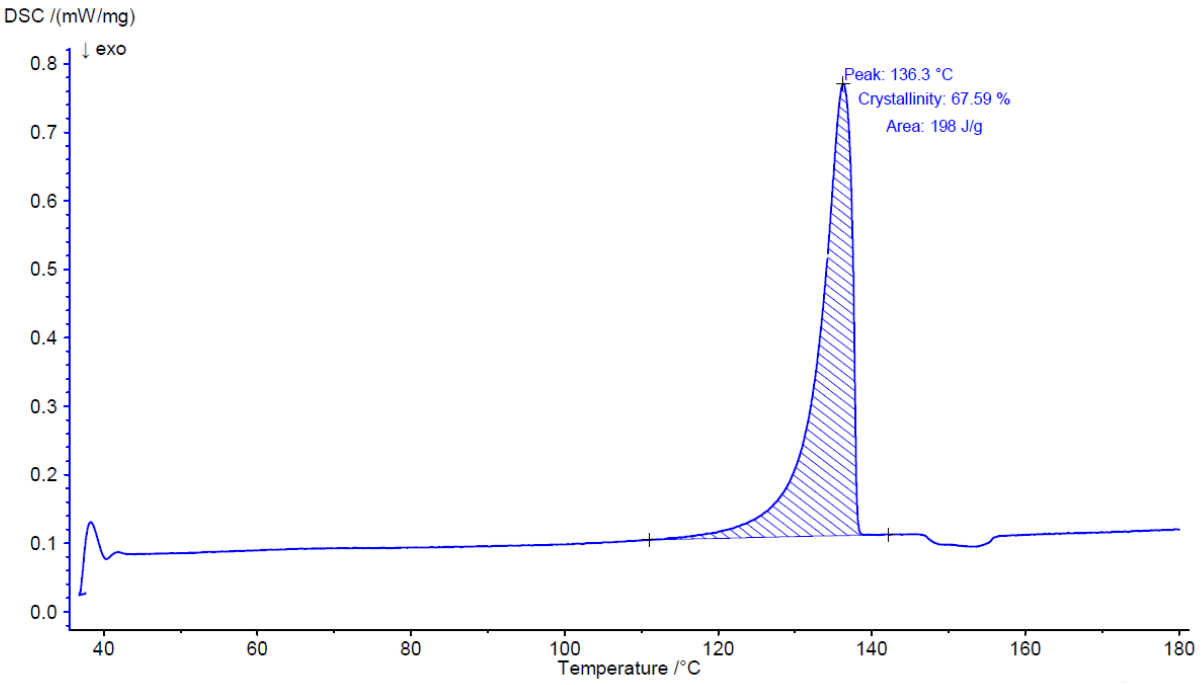

Supplementary Figure 52. DSC traces of polyethylene obtained by aqueous polymerization (Table 2, entry 4). Top: measured with 10 K min^{-1} heating rate (black curve: 1st heating, red curve: 1st cooling, blue curve: 2nd heating). Bottom: measured with 1 K min^{-1} (only first heating shown).

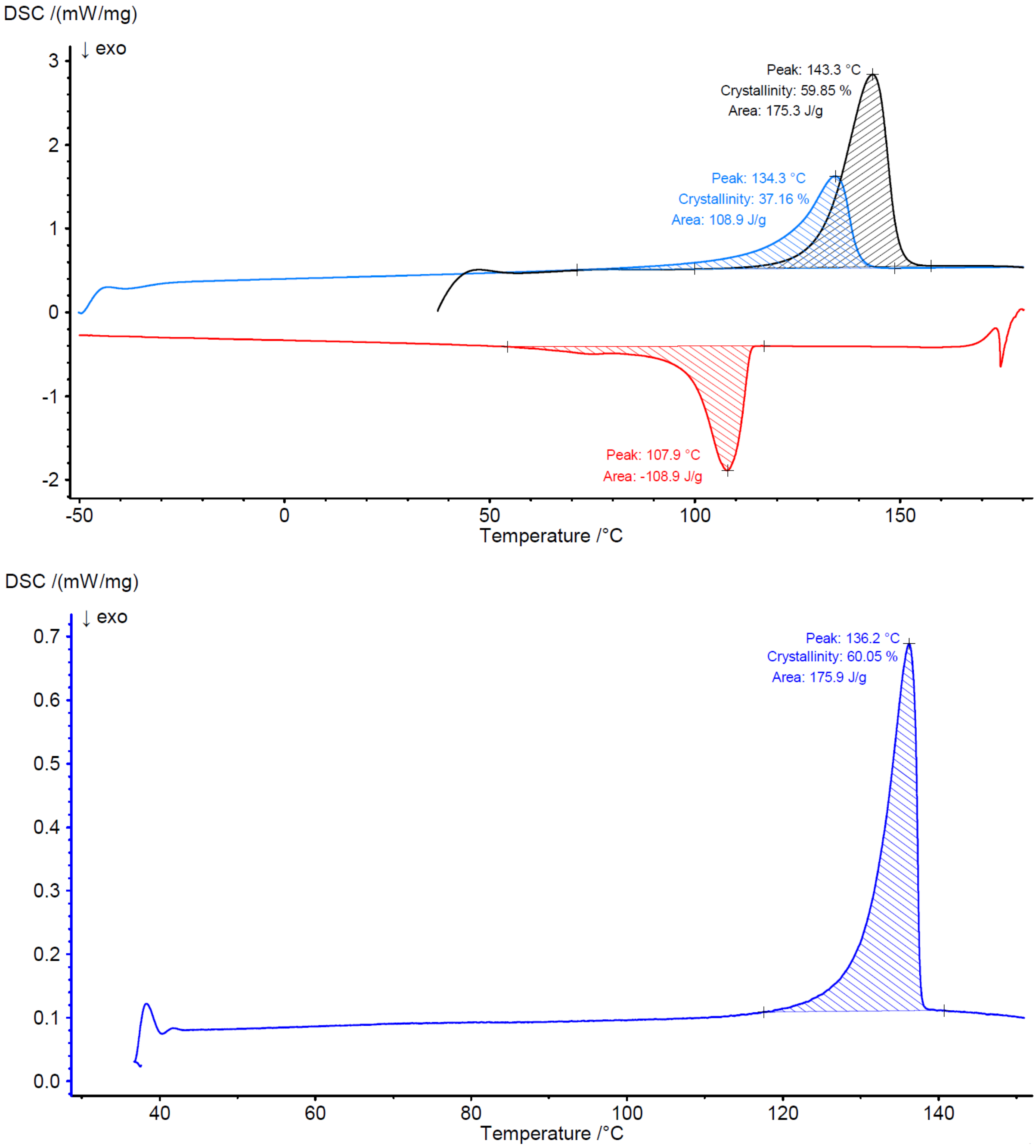

Supplementary Figure 53. DSC traces of polyethylene obtained by aqueous polymerization (Table 2, entry 5). Top: measured with 10 K min^{-1} heating rate (black curve: 1st heating, red curve: 1st cooling, blue curve: 2nd heating). Bottom: measured with 1 K min^{-1} (only first heating shown).

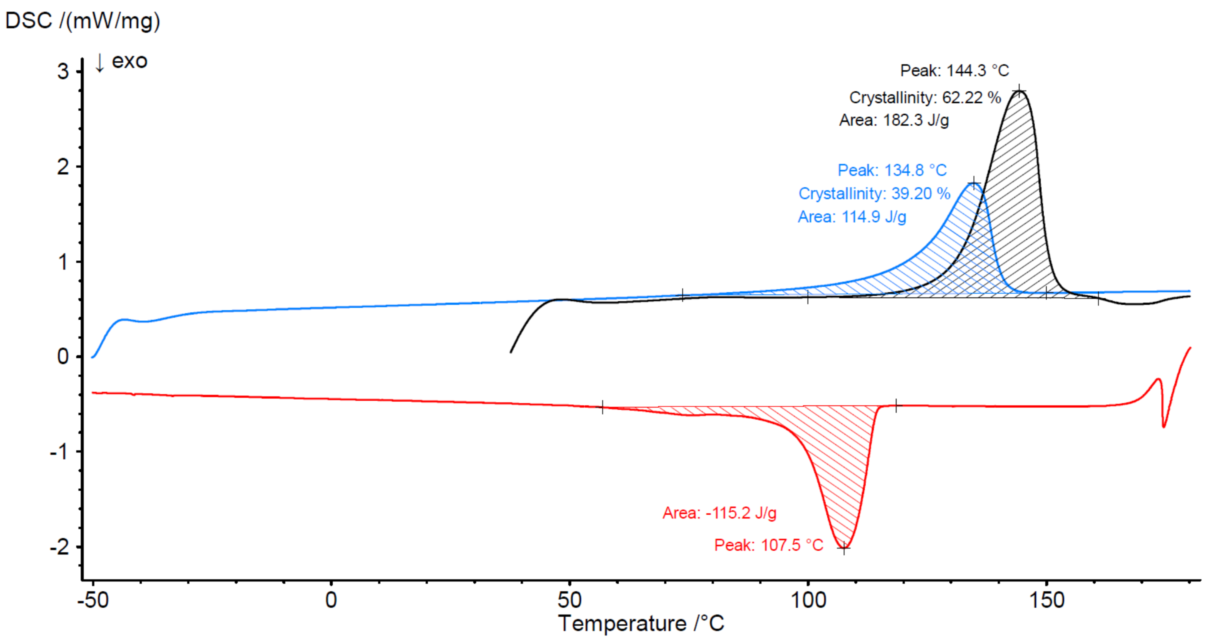

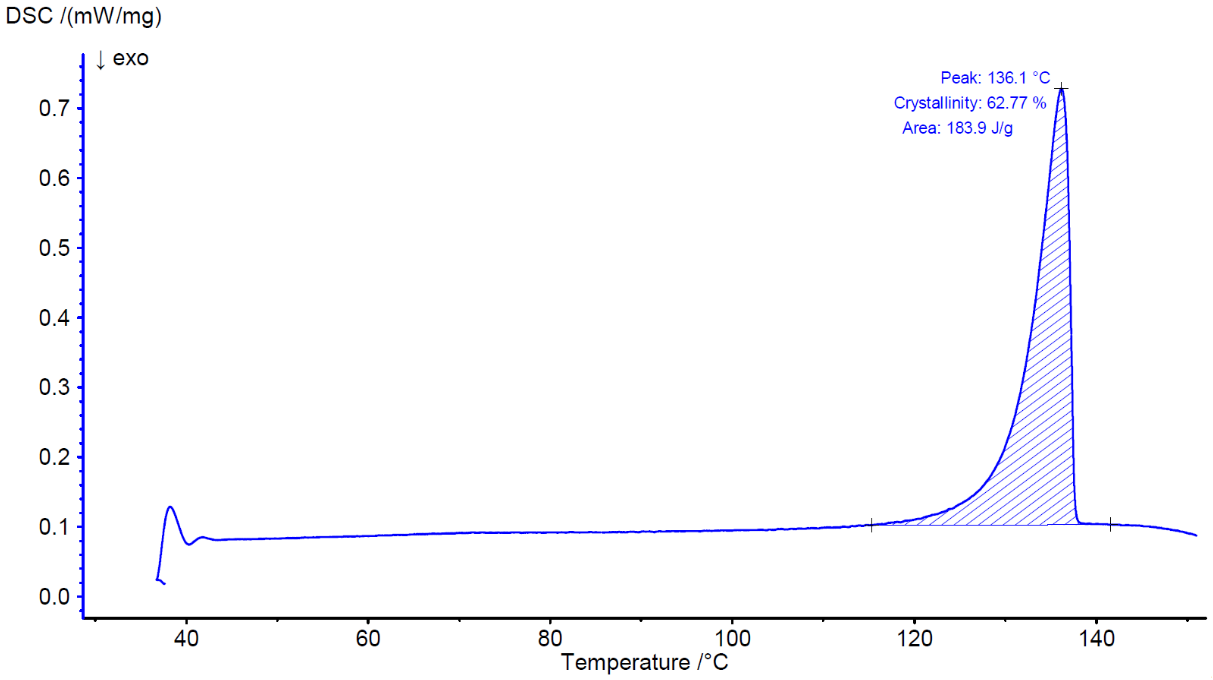

Supplementary Figure 54. DSC traces of polyethylene obtained by aqueous polymerization (Table 2, entry 6). Top: measured with 10 K min^{-1} heating rate (black curve: 1st heating, red curve: 1st cooling, blue curve: 2nd heating). Bottom: measured with 1 K min^{-1} (only first heating shown).

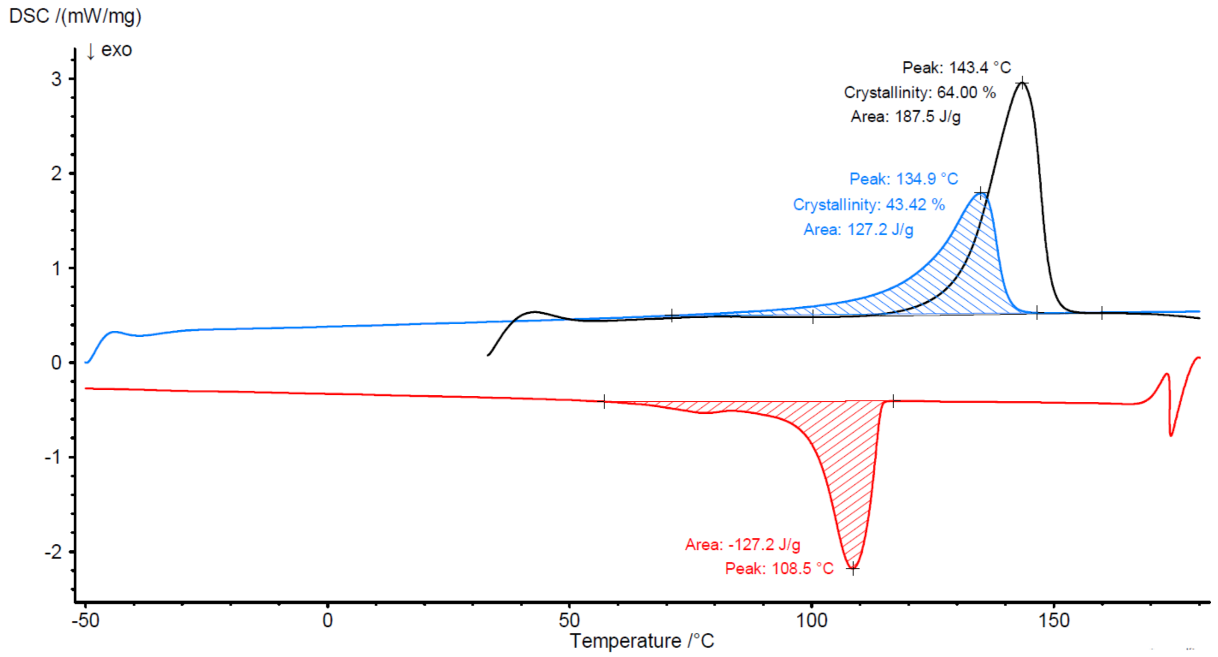

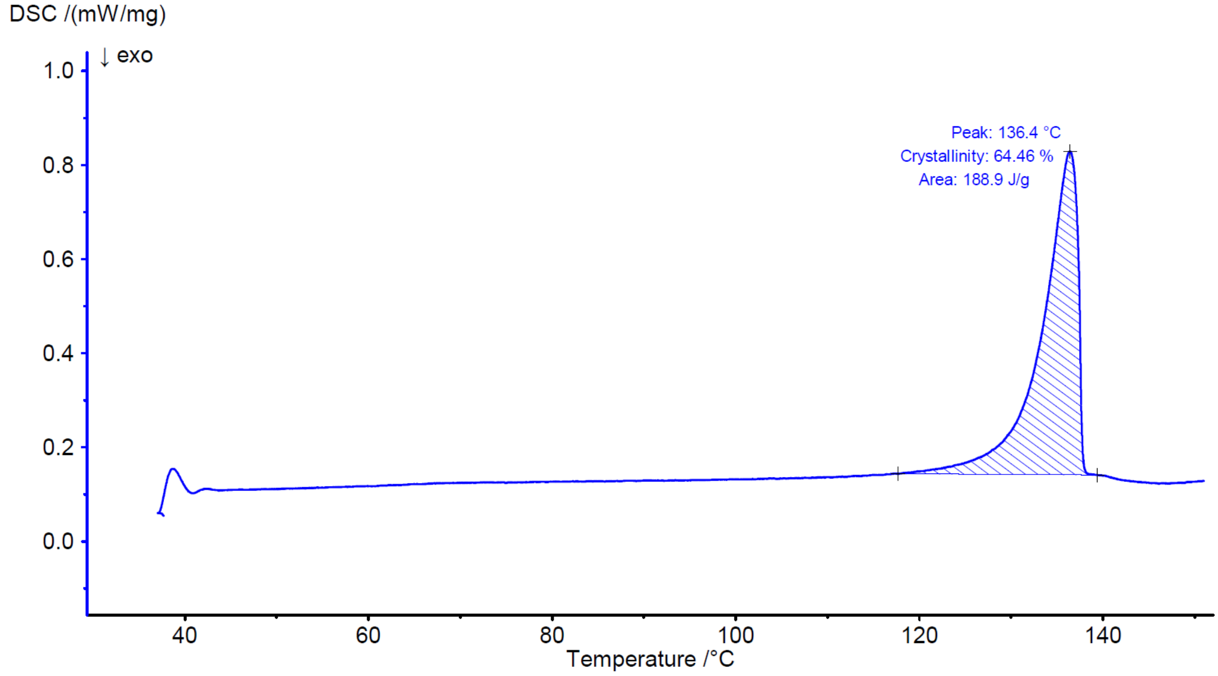

Supplementary Figure 55. DSC traces of polyethylene obtained by aqueous polymerization (Table 2, entry 7). Top: measured with 10 K min^{-1} heating rate (black curve: 1st heating, red curve: 1st cooling, blue curve: 2nd heating). Bottom: measured with 1 K min^{-1} (only first heating shown).

3.6 DLS data of nanocrystal dispersions

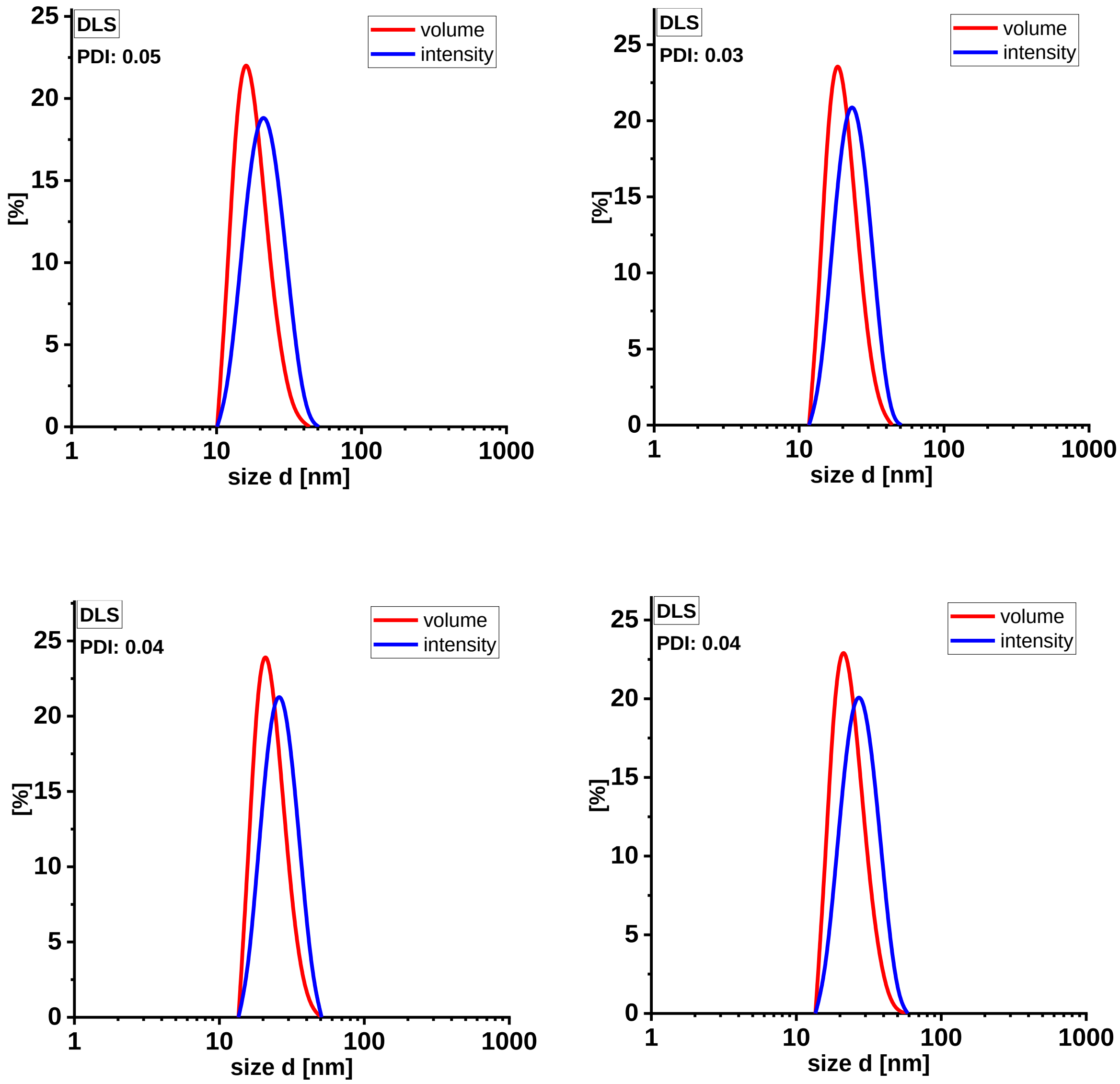

Supplementary Figure 56. DLS traces of polyethylene dispersions (Table 2). Volume- and intensity-based distributions shown. PDI according to Malvern Zetasizer software. Top, left: entry 1 | top, right: entry 2 | bottom, left: entry 3 | bottom, right: entry 4.

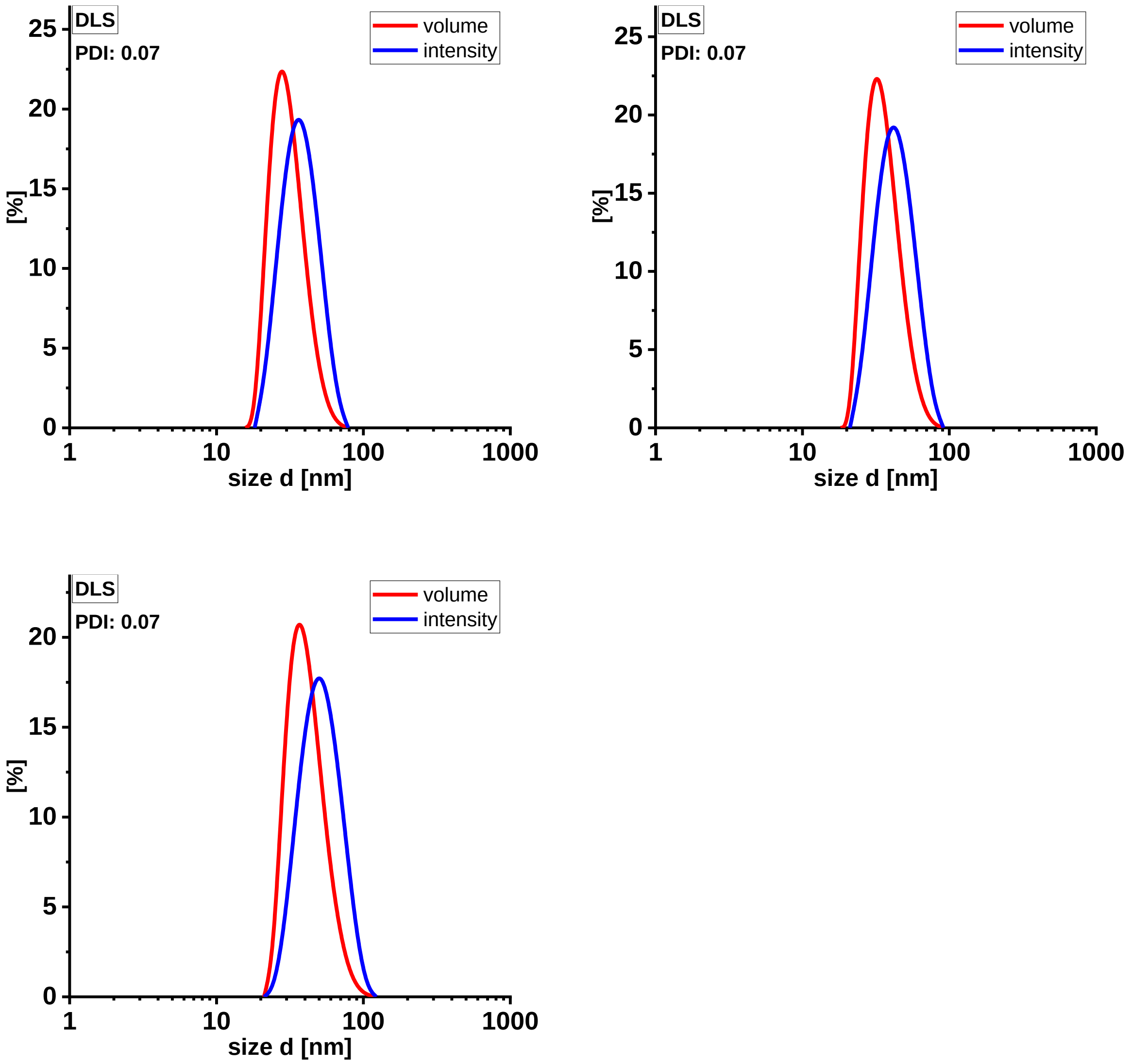

Supplementary Figure 57. DLS traces of polyethylene dispersions (Table 2). Volume- and intensity-based distributions shown. PDI according to Malvern Zetasizer software. Top, left: entry 5 | top, right: entry 6 | bottom: entry 7.

3.7 NMR spectra of synthesized polyethylenes

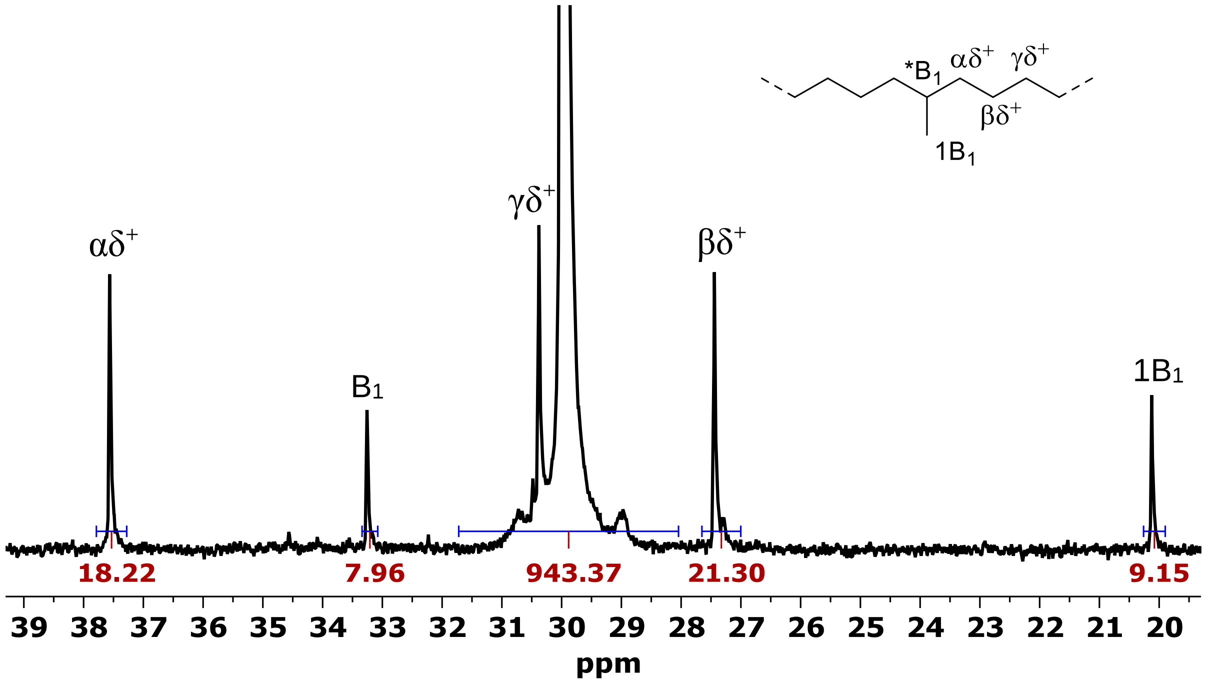

Supplementary Figure 58. ^{13}C NMR spectrum (151 MHz, $C_2D_2Cl_4$, 388 K) of polyethylene obtained from polymerization in toluene (Table 1, entry 1).

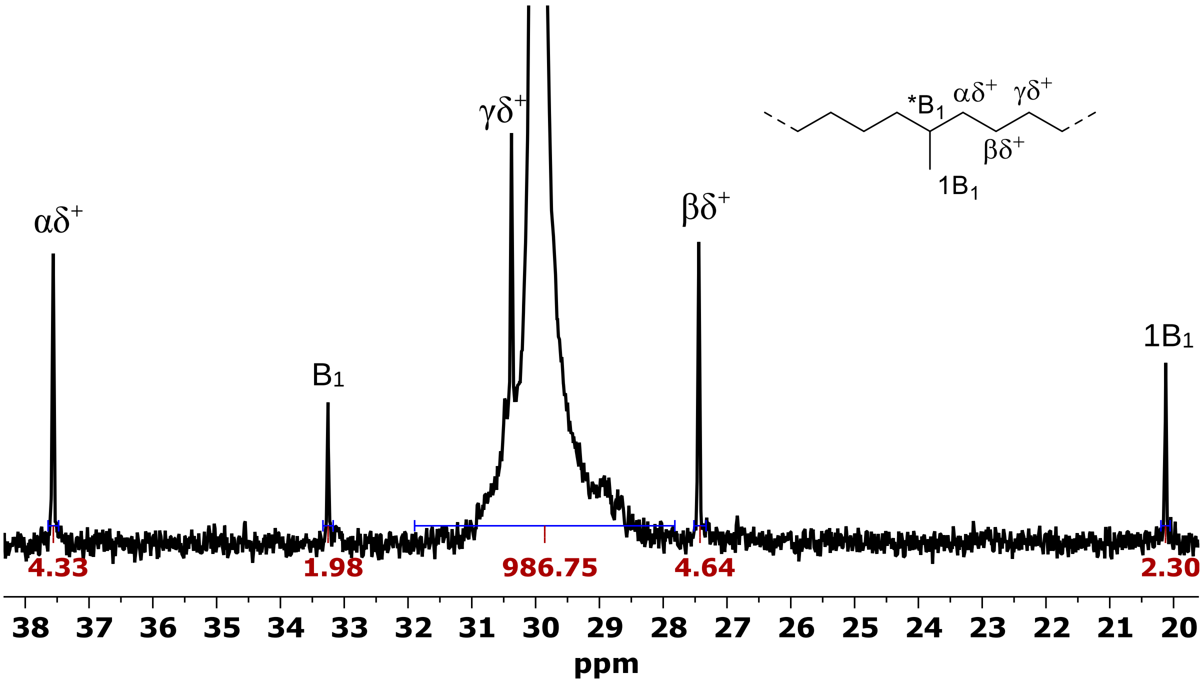

Supplementary Figure 59. ^{13}C NMR spectrum (151 MHz, $C_2D_2Cl_4$, 388 K) of polyethylene obtained from polymerization in toluene (Table 1, entry 5).

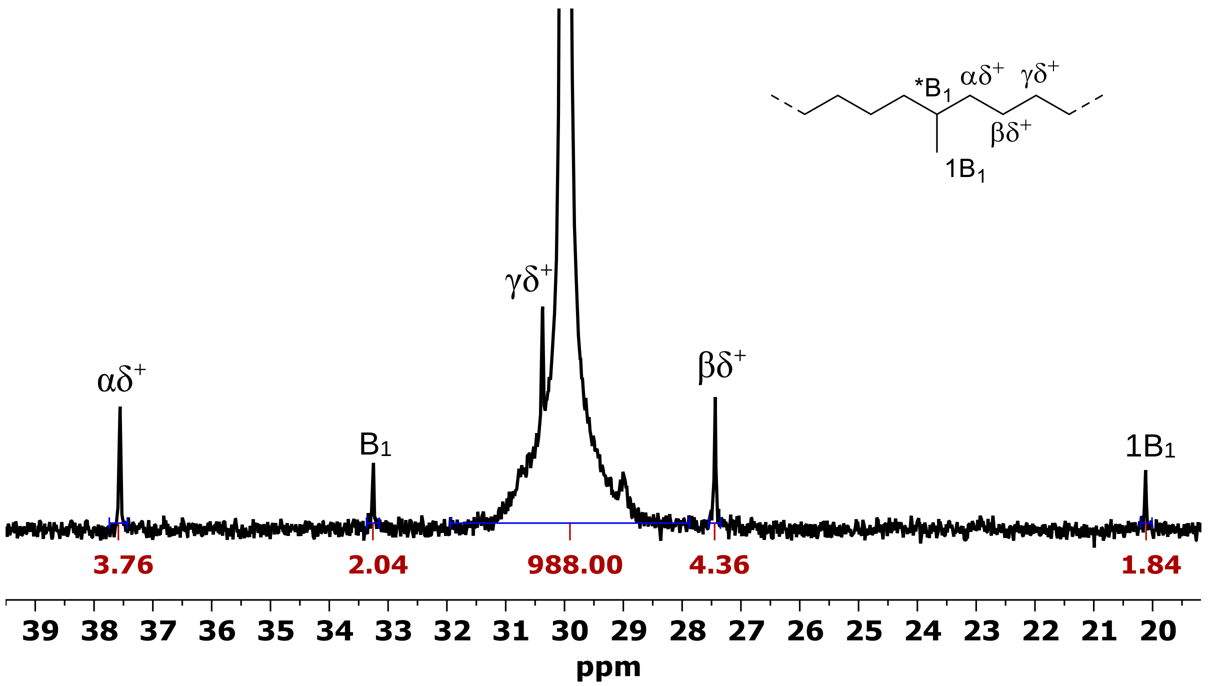

Supplementary Figure 60. ^{13}C NMR spectrum (151 MHz, $C_2D_2Cl_4$, 388 K) of polyethylene obtained from polymerization in toluene (Table 1, entry 8).

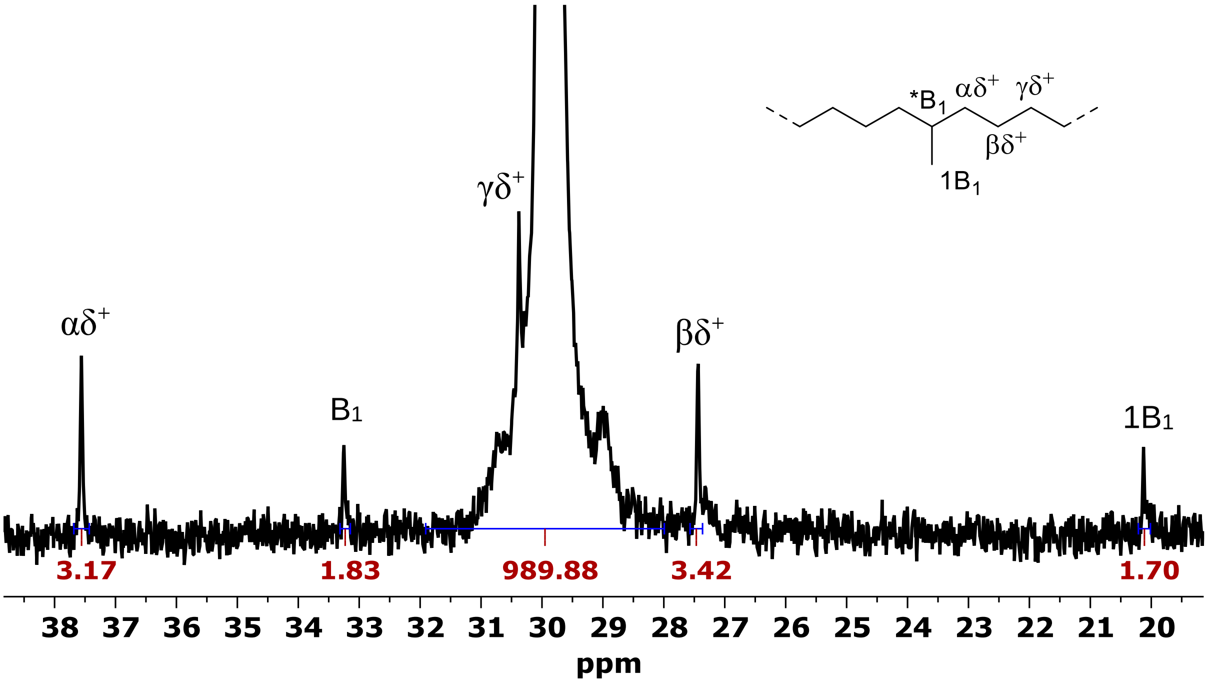

Supplementary Figure 61. ^{13}C NMR spectrum (151 MHz, $C_2D_2Cl_4$, 388 K) of polyethylene obtained from polymerization in toluene (Table 1, entry 12).

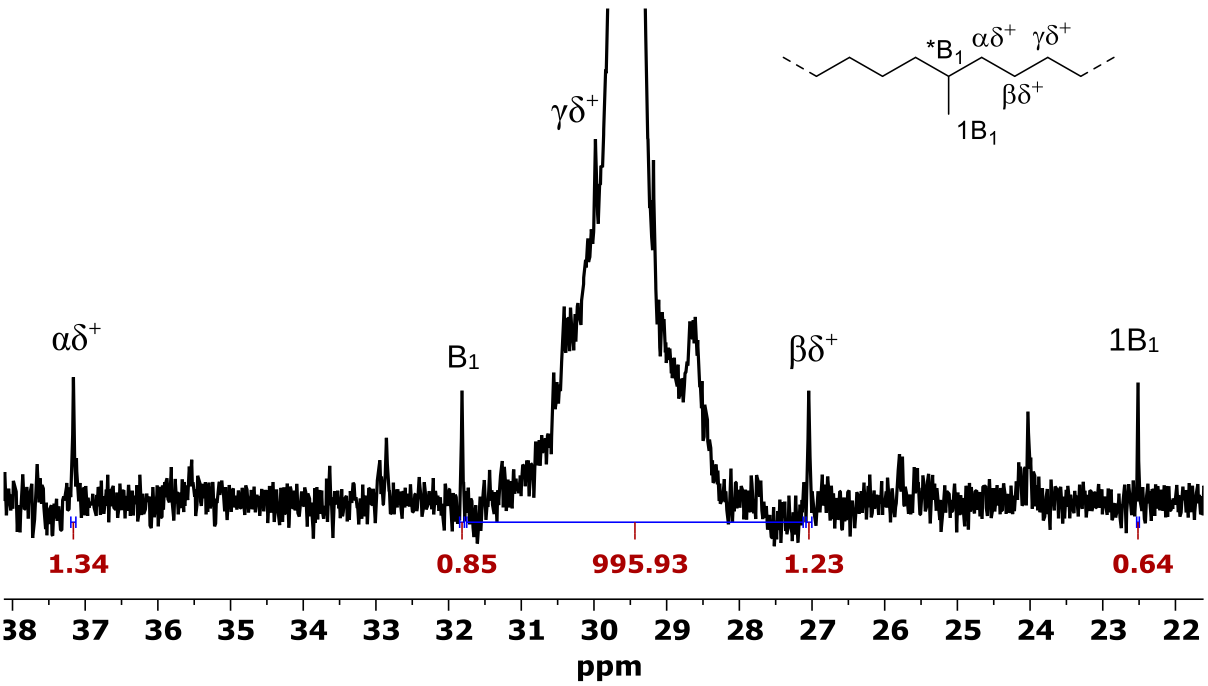

Supplementary Figure 62. ^{13}C NMR spectrum (151 MHz, $C_2D_2Cl_4$, 388 K) of polyethylene obtained from polymerization in aqueous surfactant solution (Table 2, entry 4).

4. Supplementary References

(1) I. Göttker-Schnetmann, P. Wehrmann, C. Röhr, S. Mecking. Substituent Effects in (κ^2-N,O)-Salicylaldiminato Nickel(II)−Methyl Pyridine Polymerization Catalysts: Terphenyls Controlling Polyethylene Microstructures. *Organometallics* **26**, 2348-2362 (2007).

(2) I. Göttker-Schnetmann, B. Korthals, S. Mecking. Water-soluble salicylaldiminato Ni(II)-methyl complexes: enhanced dissociative activation for ethylene polymerization with unprecedented nanoparticle formation. *J. Am. Chem. Soc.* **128**, 7708-7709 (2006).

(3) J. L. Herde, J. C. Lambert, C. V. Senoff, M. A. Cushing. Cyclooctene and 1,5-Cyclooctadiene Complexes of Iridium(I). *Inorg. Synth.* **5**, 18-20 (2007).

(4) S. K. Ghosh, A. S. Ojeda, J. Guerrero-Leal, N. Bhuvanesh, J. A. Gladysz. New media for classical coordination chemistry: phase transfer of Werner and related polycations into highly nonpolar fluorous solvents. *Inorg. Chem.* **52**, 9369-9378 (2013).

(5) T. Ishiyama, J. Takagi, K. Ishida, N. Miyaura, N. R. Anastasi, J. F. Hartwig. Mild Iridium-Catalyzed Borylation of Arenes. High Turnover Numbers, Room Temperature Reactions, and Isolation of a Potential Intermediate. *J. Am. Chem. Soc.* **124**, 390-391 (2002).

(6) S. S. Davis, A. Smith. "19 - The Influence of the Disperse Phase on the Stability of Oil-in-Water Emulsions" in *Theory and Practice of Emulsion Technology*, A. L. Smith, Ed. (Academic Press, 1976), 325-346.

(7) A. Godin, I. Göttker-Schnetmann, S. Mecking. Nanocrystal Formation in Aqueous Insertion Polymerization. *Macromolecules* **49**, 8825-8837 (2016).

(8) J. Schindelin, I. Arganda-Carreras, E. Frise, V. Kaynig, M. Longair, T. Pietzsch, S. Preibisch, C. Rueden, S. Saalfeld, B. Schmid, J.-Y. Tinevez, D. J. White, V. Hartenstein, K. Eliceiri, P. Tomancak, A. Cardona. Fiji: an open-source platform for biological-image analysis. *Nat. Methods* **9**, 676-682 (2012).

(9) C. T. Rueden, J. Schindelin, M. C. Hiner, B. E. DeZonia, A. E. Walter, E. T. Arena, K. W. Eliceiri. ImageJ2: ImageJ for the next generation of scientific image data. *BMC Bioinformatics* **2017,** *18* (1), 529.

(10) C. A. Schneider, W. S. Rasband, K. W. Eliceiri. NIH Image to ImageJ: 25 years of image analysis. *Nat. Methods* **9**, 671-675 (2012).

(11) K. S. Whiteley. "Polyethylene" in *Ullmann's Encyclopedia of Industrial Chemistry* (Wiley-VCH Verlag GmbH & Co. KGaA, Weinheim, 2005)
